# Supplementary material for: Comparative Connectomics Reveals How Partner Identity, Location, and Activity Specify Synaptic Connectivity in Drosophila
Source: Neuron. 2021 Jan 6;109(1):105–122.e7. doi: 10.1016/j.neuron.2020.10.004 (PMC7837116; doi:10.1016/j.neuron.2020.10.004)
Supplement: Document S1. Figures S1–S7, Tables S1–S3, and Data S1–S3 [file mmc1.pdf]

Neuron, Volume *109*

Supplemental Information

Comparative Connectomics Reveals How Partner Identity, Location, and Activity Specify Synaptic Connectivity in *Drosophila*

Javier Valdes-Aleman, Richard D. Fetter, Emily C. Sales, Emily L. Heckman, Lalanti Venkatasubramanian, Chris Q. Doe, Matthias Landgraf, Albert Cardona, and Marta Zlatic

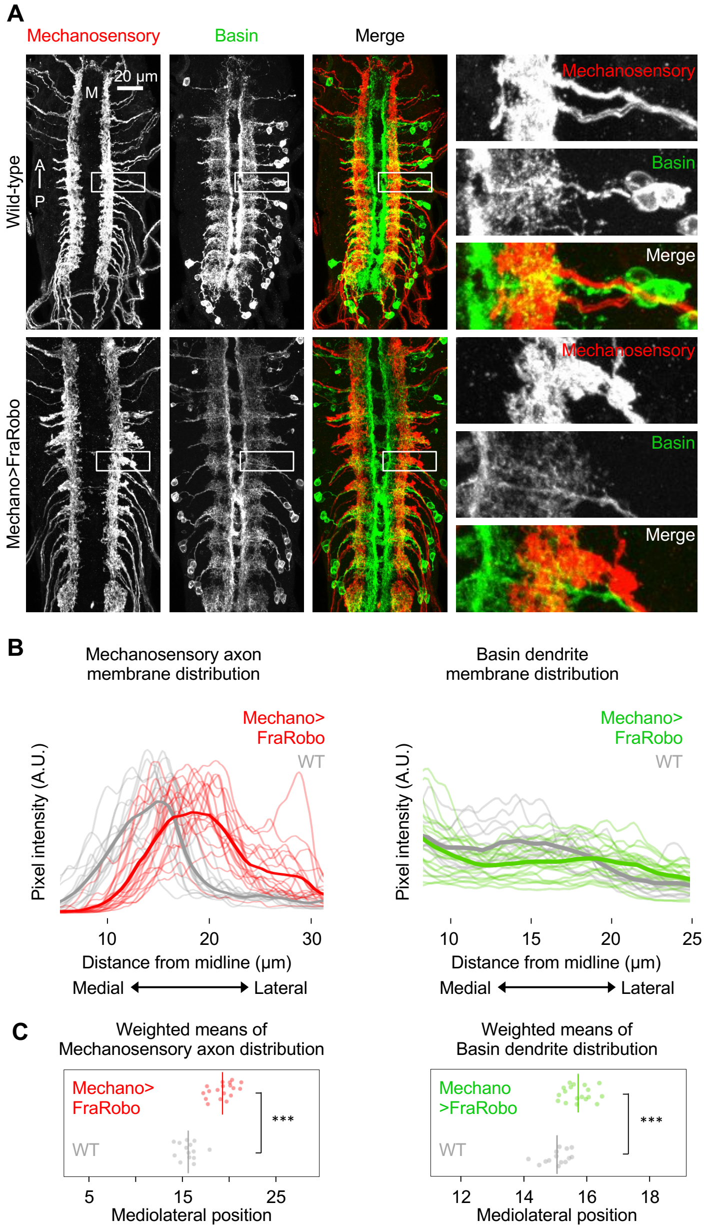

Figure S1. Light imaging of displacement of mechanosensory neuron axons and postsynaptic Basin dendrites. Related to Figure 3.

A) Representative Z-projections of confocal images of mechanosensory neurons and Basin interneurons in the nerve cord of 3rd instar larvae expressing FraRobo in mechanosensory neurons (mechano>FraRobo) and wild-type. White squares enlarged to the right. M, midline.

B) Pixel distribution plots for mechanosensory axons and Basin interneurons in a hemisegment. Left and right sides of multiple segments were compiled in a single hemisegment representation. Bold lines represent mean traces, faded lines represent individual hemisegments. n= 14 hemisegments for wild-type; n= 18 hemisegments for mechano>FraRobo.

C) Weighted mean of the distribution of mechanosensory axons and Basin dendrites. The mediolateral range considered for quantification is shown in B. n= 14 hemisegments for wild-type; n= 18 hemisegments for mechano>FraRobo. Data compared using an unpaired t-test. ***, P < 0.001.

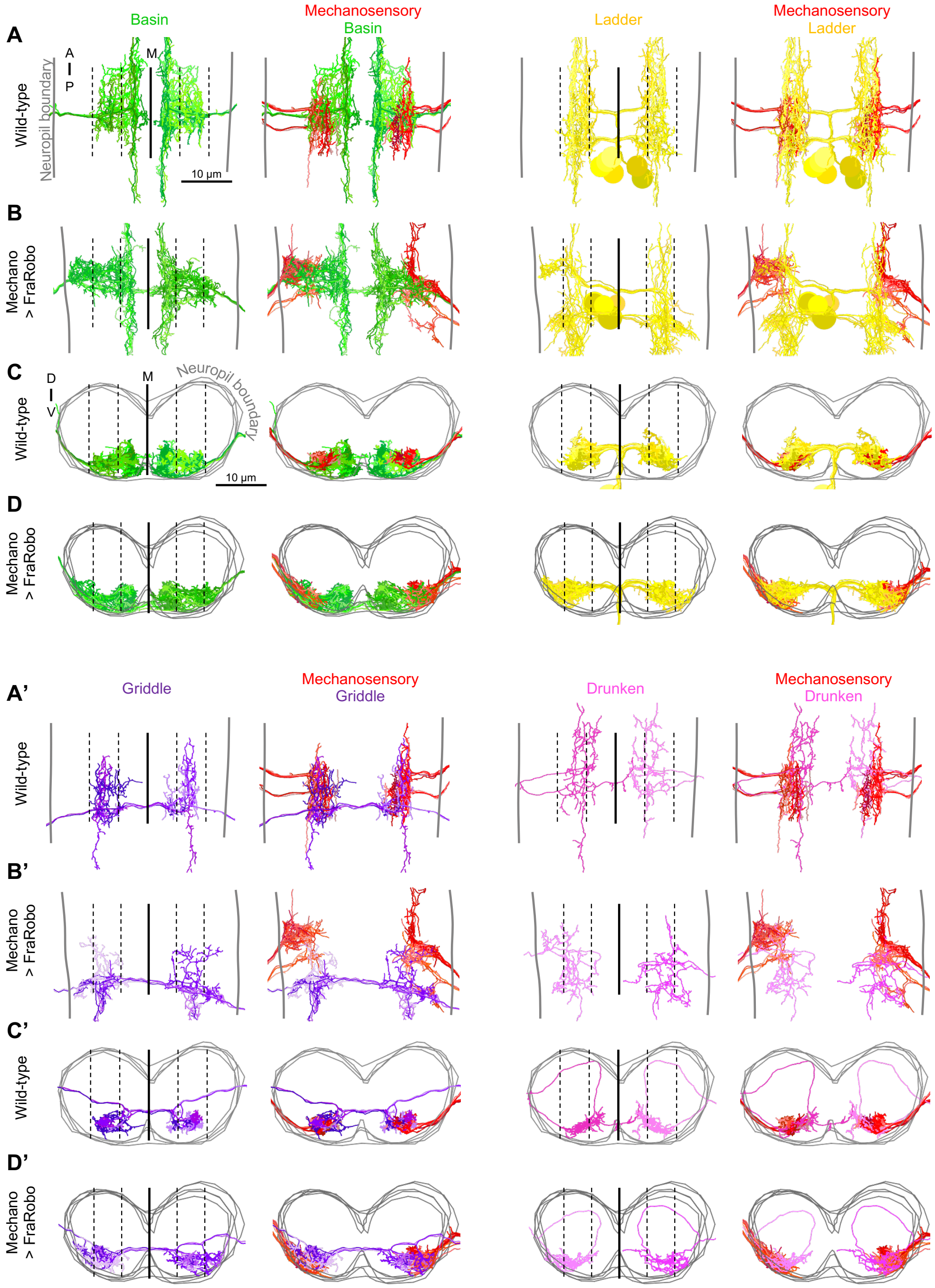

Figure S2. Full neuron (axon, dendrites and neuropil entry point) view of partner cells and their overlap with mechanosensory neurons. Related to Figures 3 and 4.

A-D') Dorsal (A-B') and cross section (C-D') views of the mechanosensory neurons and their preferred partners in wild-type (A, A', C and C') and the mechano>FraRobo (B, B', D and D') volumes. The neuropil boundary is represented by either a pair of gray vertical lines for dorsal views (A-B') or gray consecutive rings for cross section views (C-D'). Dashed lines split the maximum width of the neuropil in six equidistant sections, three on either side of the midline (M).

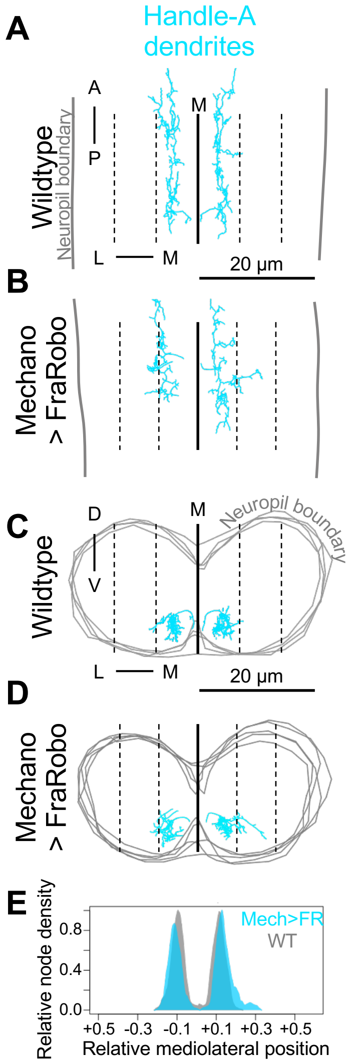

Figure S3. Displacement of mechanosensory axons does not affect the position of non-partner neurons. Related to Figures 3 and 4.

A-D) Dorsal (A-B) and cross section (C-D) views of the dendrites of Handle-A in wild-type (A and C) and the mechano>FraRobo (B and D) volumes. Handle-A does not receive direct input from the mechanosensory neurons. The mediolateral position of its dendrites is not affected by the lateral displacement of the mechanosensory axons.

E) Node density distribution of Handle-A in the mediolateral axis in wild-type (WT) and mechano>FraRobo (mech>FR).

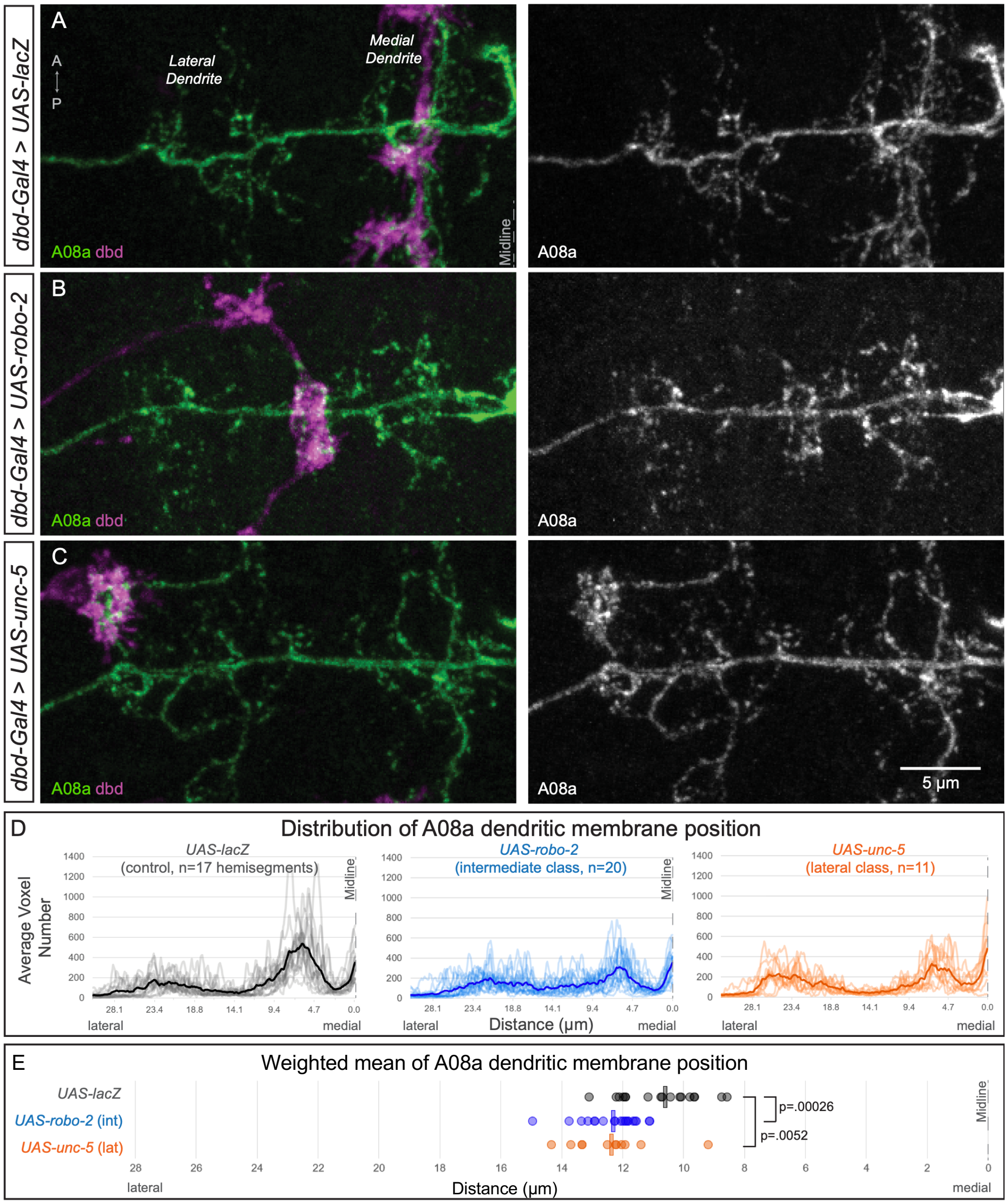

Figure S4. Displacement of dbd sensory neuron axons causes their postsynaptic partner dendrites to follow. Related to Figure 3.

A-C) Confocal maximum intensity projection of the dorsal view of dbd axon terminal (magenta) and the A08a dendritic domain (green) in one hemisegment of a 3rd instar larva. Merged channels shown to the left; A08a channel shown to the right.

A) In wild-type, dbd axon targets the A08a medial arbor. n=17 hemisegments from 11 animals.

B) dbd axons expressing Robo-2 are shifted laterally and often contact the A08a intermediate domain. n=20 hemisegments from 10 animals.

C) dbd expressing Unc-5 often contact the A08a lateral arbor. n=11 hemisegments from 10 animals.

D) Quantification of the distribution of A08a dendrite position in the context of medial, intermediate, and lateral dbd axons. Transparent lines represent individual hemisegments and each solid line represents the average for the cohort.

E) Weighted mean of the dendrite distributions shown in D. Each circle represents one hemisegment and bars represent the average weighted mean for each cohort. Average weighted mean: *UAS-lacZ*: 10.63 µm; *UAS-robo-2*, intermediate: 12.33 µm; *UAS-unc-5*, lateral: 12.38 µm. P-values were obtained using an unpaired t-test.

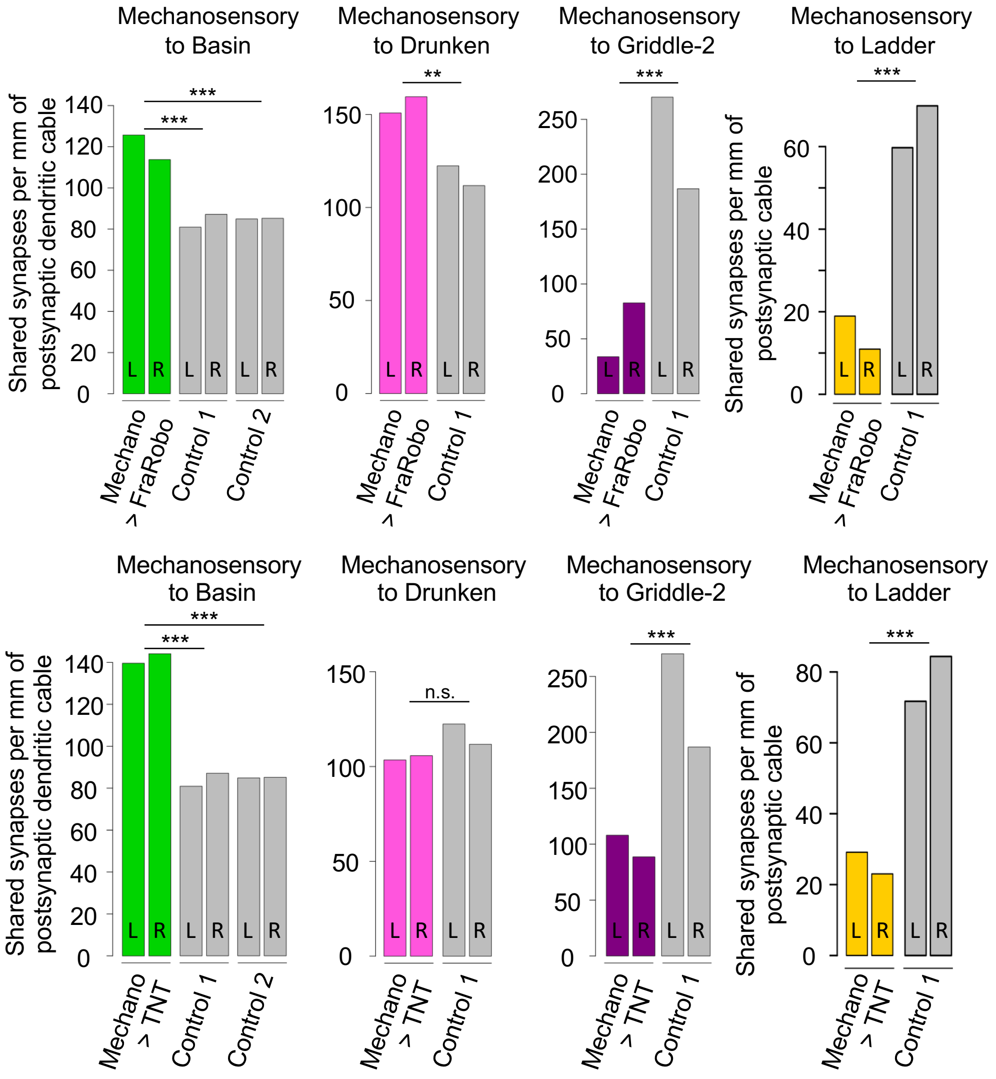

Figure S5. Connectivity relative to cable length between mechanosensory neurons and their preferred postsynaptic partners in the mechano>FraRobo and mechano>TNT EM volumes. Related to Figures 5 and 6.

Number of synapses from mechanosensory neurons onto postsynaptic partners per mm of cable length. Chi-square test. **, $P < 0.01$, and ***, $P < 0.001$.

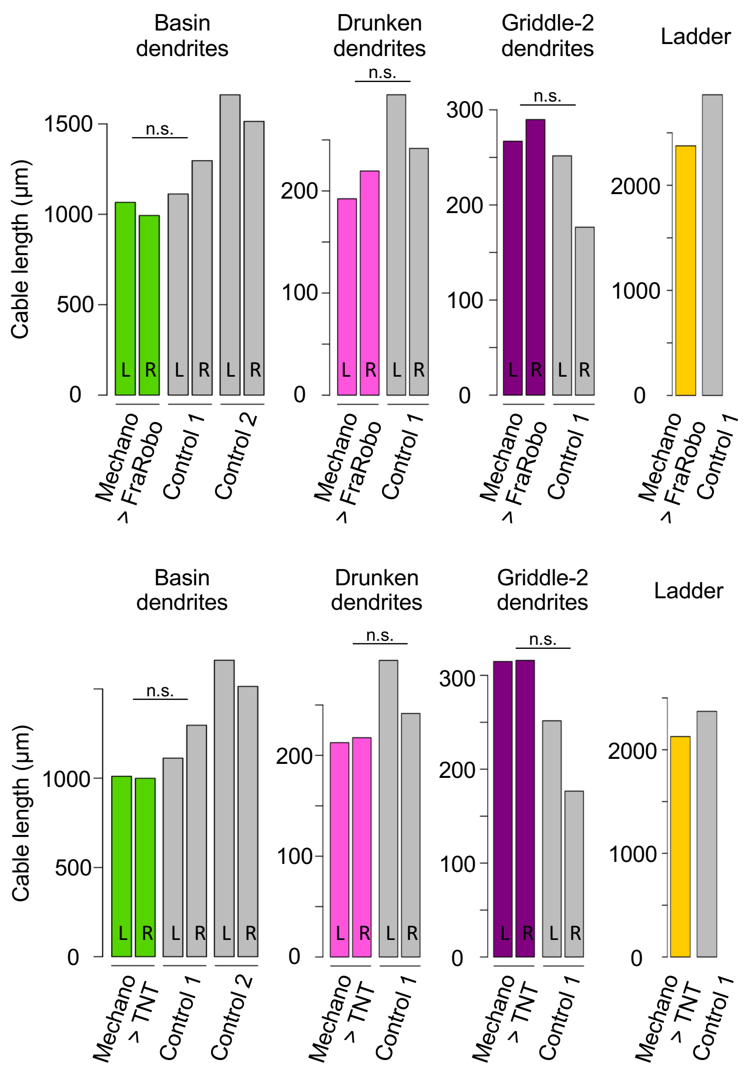

Figure S6. Cable length of reconstructed interneurons in the mechano>FraRobo and mechano>TNT EM volumes. Related to Figures 5, 6 and 7.

Total linearized distance of the reconstructed neurons of interest in the left (L) and right (R) hemisegments of the mechano>FraRobo, mechano>TNT, control-1 (1st instar), control-2 (older 1st instar) EM volumes. Axonal and dendritic distance shown for Ladder. There are no left and right homologous cells for ladder, as their cell bodies are medial with symmetrical bilateral projections. The quantification of the cable length for ladder in control-1 is adjusted based on the size and position of the corresponding control subvolume it is compared to (see Methods). Cable length compared with t-test, n=2 hemisegments.

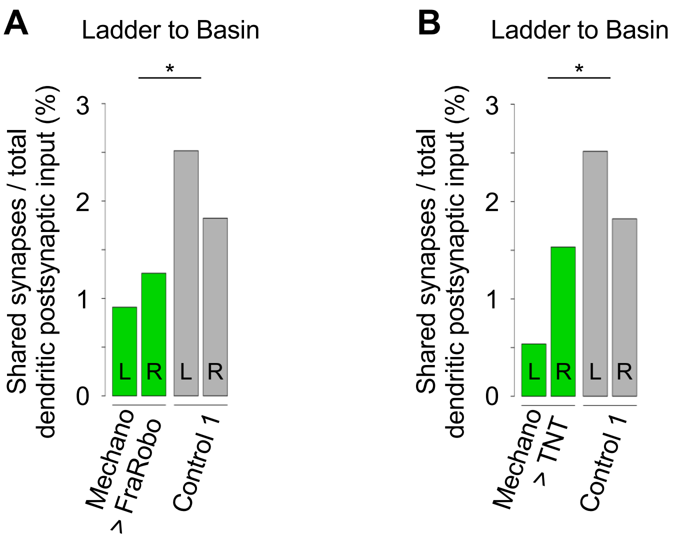

Figure S7. Connectivity between interneurons is decreased in the mechano>FraRobo and mechano>TNT EM volumes. Related to Figures 5, 6 and 7.

The fraction of input from inhibitory Ladders onto Basin interneurons is decreased when the mechanosensory neurons were shifted by the expression of FraRobo (A) or silenced by the expression of TNT (B). This reduction in connectivity between Ladder and Basin interneurons may be at the expense of the increase in connections between sensory neurons (mechanosensory and/or nociceptive) and Basin interneurons under the same conditions (Figures 5E, 6B and 7A). Chi-square test. *, $P < 0.05$.

Direction	mechano-FraRobo	mechano-TNT	WT-1	WT-2
Mechano_to_Basin_L	134	141	90	141
Mechano_to_Basin_R	113	144	113	129
Mechano_to_Noci_L	0	0	0	NA
Mechano_to_Noci_R	0	0	0	NA
Mechano_to_Drunken-1_L	29	22	36	NA
Mechano_to_Drunken-1_R	35	23	27	NA
Mechano_to_Griddle-2_L	9	34	68	NA
Mechano_to_Griddle-2_R	24	28	33	NA
Mechano_to_Ladder_L	45	62	171	NA
Mechano_to_Ladder_R	26	49	200	NA
Noci_to_Basin_L	NA	52	51	NA
Noci_to_Basin_R	NA	61	57	NA
Ladder_to_Basin_L	6	3	23	NA
Ladder_to_Basin_R	7	9	19	NA

Table S1. Connectivity data. Related to Figures 5E-H, 6B-E and 7A.

Synapse counts between mechanosensory or nociceptive neurons and their preferred partners. Connectivity from the mechano>FraRobo and mechano>TNT EM volumes generated for this study, and from the previously reported control-1 (WT-1) and control-2 (WT-2) EM volumes (Jovanic et al., 2016; Ohyama et al., 2015).

Group	Cable length	Presynaptic sites	Postsynaptic sites	Volume	Sub arbor	Side
Mechano	461567.848	430	225	WT-1	axon	L
Mechano	483976.708	437	203	WT-1	axon	R
Nociceptive	226464.851	148	38	WT-1	axon	L
Nociceptive	228380.665	162	47	WT-1	axon	R
Ladder	2370566.76	338	1333	WT-1 (to compare with TNT)	whole	Bi
Ladder	2862200.75	409	1556	WT-1 (to compare with FraRobo)	whole	Bi
Basin	1112276.31	38	914	WT-1	dendrites	L
Basin	1296695.63	32	1042	WT-1	dendrites	R
Griddle-2	251606.121	41	179	WT-1	dendrites	L
Griddle-2	176651.487	38	110	WT-1	dendrites	R
Drunken-1	294221.904	26	206	WT-1	dendrites	L
Drunken-1	241624.37	29	161	WT-1	dendrites	R
Mechano	738345.826	226	133	mechano-FraRobo	axon	L
Mechano	699198.101	202	122	mechano-FraRobo	axon	R
Nociceptive	282349.661	105	54	mechano-FraRobo	axon	L
Nociceptive	287000.292	98	41	mechano-FraRobo	axon	R
Ladder	2375634.7	323	1000	mechano-FraRobo	whole	Bi
Basin	1066024.36	17	659	mechano-FraRobo	dendrites	L
Basin	993480.482	19	556	mechano-FraRobo	dendrites	R
Griddle-2	266985.898	25	135	mechano-FraRobo	dendrites	L
Griddle-2	289732.528	23	174	mechano-FraRobo	dendrites	R
Drunken-1	192320.879	11	136	mechano-FraRobo	dendrites	L
Drunken-1	219498.444	14	131	mechano-FraRobo	dendrites	R
Mechano	485055.1	216	87	mechano-TNT	axon	L
Mechano	361425.439	217	137	mechano-TNT	axon	R
Nociceptive	201983.643	133	59	mechano-TNT	axon	L
Nociceptive	208142.3	106	68	mechano-TNT	axon	R
Basin	1010526.71	12	558	mechano-TNT	dendrites	L
Basin	999269.61	16	587	mechano-TNT	dendrites	R
Ladder	2127555.75	336	901	mechano-TNT	whole	Bi
Griddle-2	314722.78	23	187	mechano-TNT	dendrites	L
Griddle-2	315893.148	22	168	mechano-TNT	dendrites	R
Drunken-1	212620.198	12	123	mechano-TNT	dendrites	L
Drunken-1	217554.555	11	111	mechano-TNT	dendrites	R
Mechano	466430.487	366	201	WT-2	axon	L
Mechano	418329.247	341	200	WT-2	axon	R
Basin	1660816.4	35	1185	WT-2	dendrites	L
Basin	1513899.48	37	1072	WT-2	dendrites	R

Table S2. EM-reconstructed neuron details. Related to Figures 5E-H, 6B-E and 7A.

Cable length (nm), presynaptic and postsynaptic sites of mechanosensory, nociceptive, Basin, Ladder, Griddle-2, and Drunken-1 neurons in mechano>FraRobo, mechano>TNT, control-1 (WT-1), or control-2 (WT-2) EM volumes.

Figure	Details	Genotype
2, Video S1	Live imaging of mechanosensory and Basin neurons	*w; R72F11-LexAp65 in JK22C, 13XLexAop2-IVS-myr::GFP in su(Hw)attP5, mhc[1]; iav-GAL4, UAS-IVS-myr::tdTomato in attP2*
3, 4, 5B-C, 5E-H, S2, S3, S5, S6, S7	Control	*[iso] Canton S G1 × w^{1118} [iso] 5905*
	Experimental	*w;; iav-GAL4/UAS-FraRobo*
5I-I’	Control	*w, LexAop2-Syn21-opGCaMP6s in su(Hw)attP8, 10XUAS-Syn21-Chrimson88-tdT-3.1 in attP18/+; R72F11-LexAp65 in JK22C/+; iav-GAL4, UAS-IVS-myr::tdTomato in attP2, UAS-FraRobo/UAS-FraRobo*
	Experimental	*w, LexAop2-Syn21-opGCaMP6s in su(Hw)attP8, 10XUAS-Syn21-Chrimson88-tdT-3.1 in attP18/+; R72F11-LexAp65 in JK22C/+; iav-GAL4, UAS-IVS-myr::tdTomato in attP2/+*
5J-J’	Control	*w;; iav-GAL4, UAS-IVS-myr::tdTomato in attP2*
	Experimental	*w;; iav-GAL4, UAS-IVS-myr::tdTomato in attP2, UAS-FraRobo*
6B-E, 7A, S5, S6, S7	Control	*[iso] Canton S G1 × w^{1118} [iso] 5905*
	Experimental	*w; UAS-TNT-E/+; iav-GAL4/+*
6G-G’	Control	*w; R61D08-LexAp65 in JK22C/+; 20xUAS-IVS-GCaMP6s 15.641 in attP2, 13XLexAop2-CsChrimson-tdTomato in VK00005/R72F11-GAL4 in attP2*
	Experimental	*w; R61D08-LexAp65 in JK22C/13XLexAop2-IVS-Syn21-Shibire-ts1-p10 in su(Hw)attP5; 20xUAS-IVS-GCaMP6s 15.641 in attP2, 13XLexAop2-CsChrimson-tdTomato in VK00005/R72F11-GAL4 in attP2*
6I-J’	Control	*;; attP2/UAS-Shibire-ts1*
	Experimental	*;; R61D08-GAL4 in attP2/UAS-Shibire-ts1*
7B-B’, D	Control	*w, QUAS-syn21-CsChrimson tdTomato_tr p10 in attP18/+; R61D08-LexAp65 in JK22C/+; R72F11-GAL4 in attP2, ppk-QF2/20xUAS-IVS-GCaMP6s 15.641 in attP2*
	Experimental	*w, QUAS-syn21-CsChrimson tdTomato_tr p10 in attP18/+; R61D08-LexAp65 in JK22C/pSW922[260b] (LexAop-TNT); R72F11-GAL4 in attP2, ppk-QF2/20xUAS-IVS-GCaMP6s 15.641 in attP2*
7D’, F	Control	*w; attP2/13XLexAop2-CsChrimson-tdTomato in attP40; ppk-LexA in attP2, 20XUAS-TTS-Shibire-ts1-p10 in VK00005/+*
	Experimental	*w; R61D08-GAL4 in attP2/13XLexAop2-CsChrimson-tdTomato in attP40; ppk-LexA in attP2, 20XUAS-TTS-Shibire-ts1-p10 in VK00005/+*
7E	Control	*w; UAS-TNT-E/ppk-LexA in attP40; pJFRC97-20XUAS-IVS-GCamp3-p10 in attP2, pJFRC26-13XLexAop2-IVS-dTrpA1-WPRE in VK00005/+*
	Experimental	*w; iav-GAL4, UAS-TNT-E/ppk-LexA in attP40; pJFRC97-20XUAS-IVS-GCaMP3-p10 in attP2, pJFRC26-13XLexAop2-IVS-dTrpA1-WPRE in VK00005/+*
7E’	Control	*w; ppk-LexA in attP40/+; attP2/LexAop-TrpA1 in VK00005, UAS-Kir 2.1*
	Experimental	*w; ppk-LexA in attP40/+; R61D08-GAL4 in attP2/LexAop-TrpA in VK00005, UAS-Kir 2.1*
S1	Control	*w; R72F11-LexAp65 in JK22C, 13XLexAop2-IVS-myr::GFP in su(Hw)attP5 ; iav-GAL4, UAS-IVS-myr::tdTomato in attP2*
	Experimental	*w; R72F11-LexAp65 in JK22C, 13XLexAop2-IVS-myr::GFP in su(Hw)attP5 ; iav-GAL4, UAS-IVS-myr::tdTomato in attP2, UAS-FraRobo*
S4	Control	*w, 10xUAS-IVS-myr::smGdP-HA in attP18, 13xLexAop2-IVS-myr::smGdP-V5 in su(Hw)attP8/+; R26F05-LexA, UAS-bruchpilot (short)-mstraw/UAS-LacZ; 165-GAL4/+*
	Expression of Robo2 in dbd neurons	*w, 10xUAS-IVS-myr::smGdP-HA in attP18, 13xLexAop2-IVS-myr::smGdP-V5 in su(Hw)attP8/+; R26F05-LexA, UAS-bruchpilot (short)-mstraw/UAS-robo-2::HA; 165-GAL4/+*
	Expression of Unc-5 in dbd neurons	*w, 10xUAS-IVS-myr::smGdP-HA in attP18, 13xLexAop2-IVS-myr::smGdP-V5 in su(Hw)attP8/+; R26F05-LexA, UAS-bruchpilot (short)-mstraw/UAS-unc-5::HA; 165-GAL4/+*

Table S3. Fly line genotypes. Related to STAR Methods.

Data S1. Neuronal atlas of mechanosensory neurons and preferred partners in the mechano>FraRobo EM volume. Related to Figures 3-5.

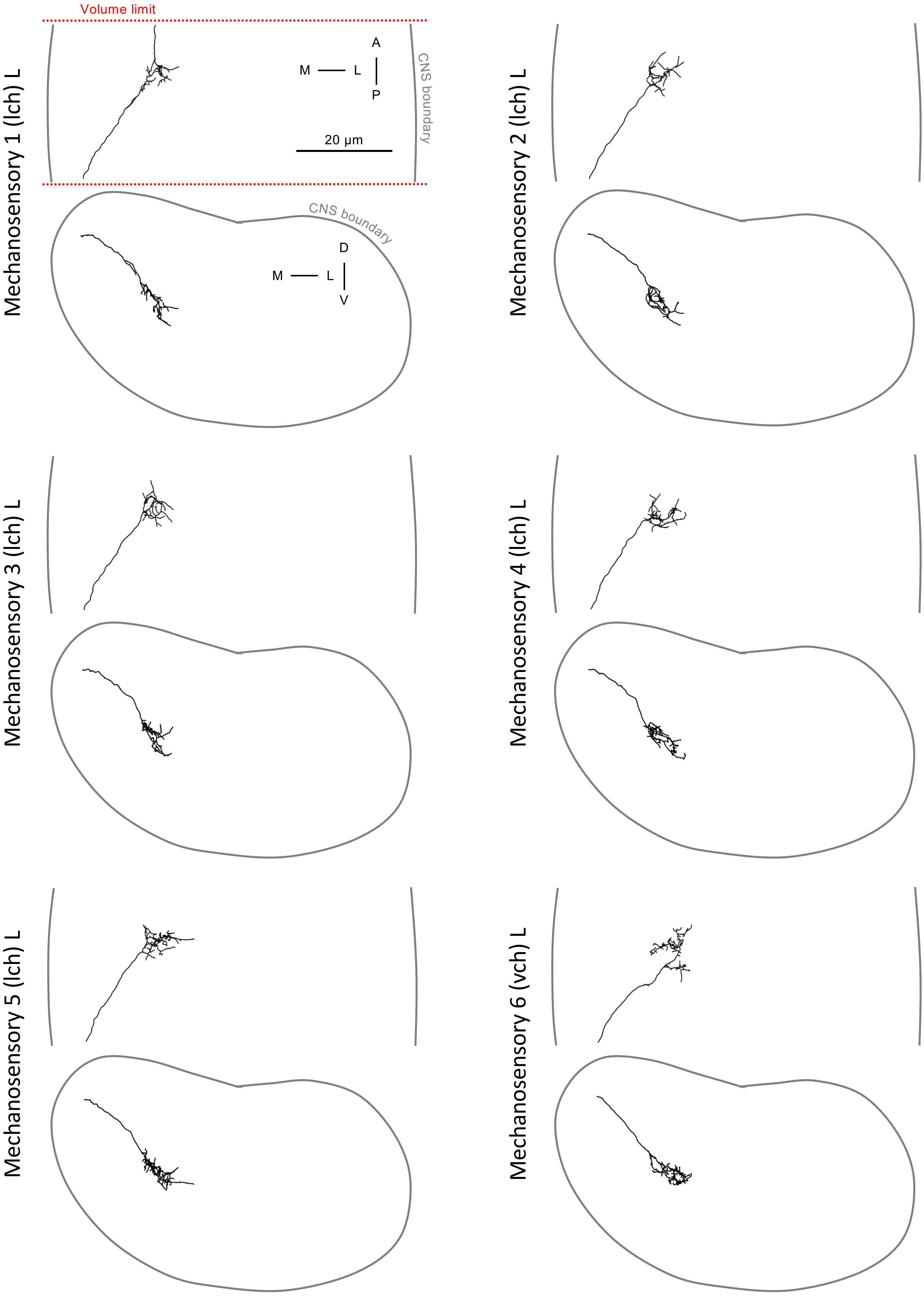

Mechanosensory 11 (lch) R

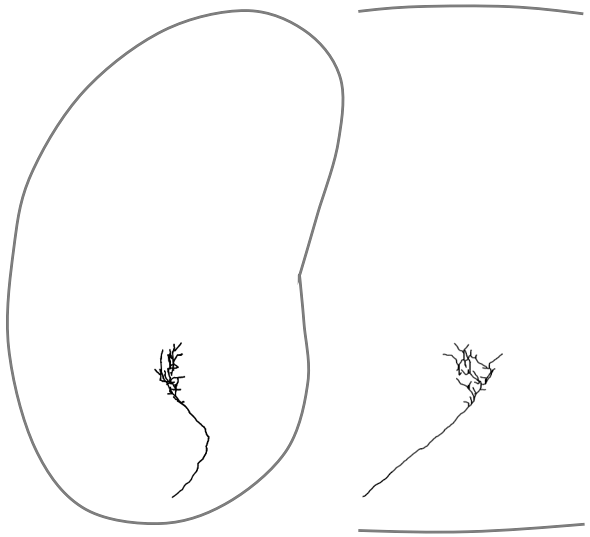

Mechanosensory 9 (lch) R

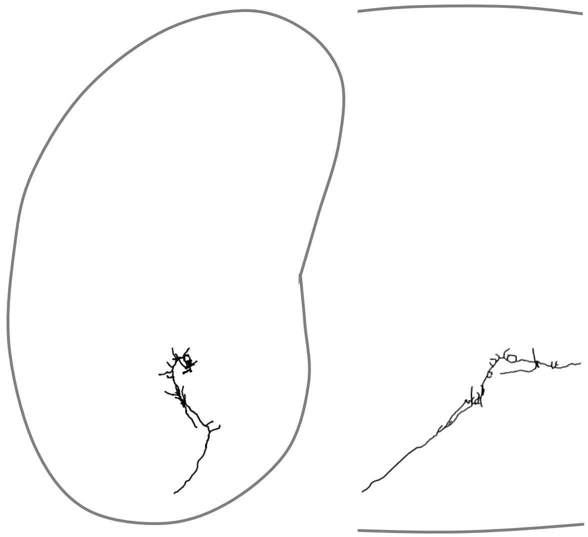

Mechanosensory 7 (vch) L

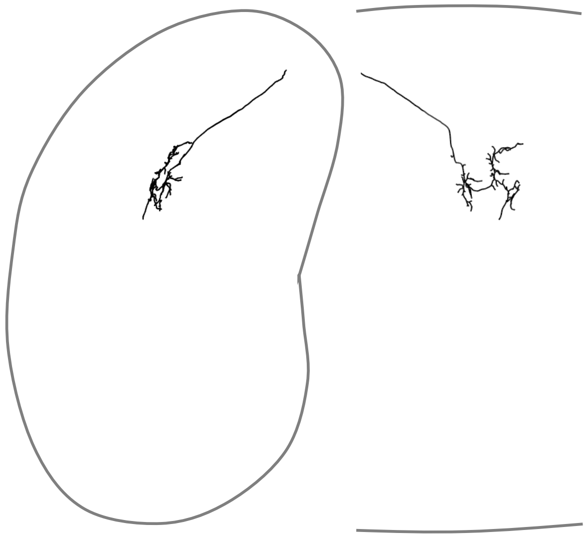

Mechanosensory 12 (lch) R

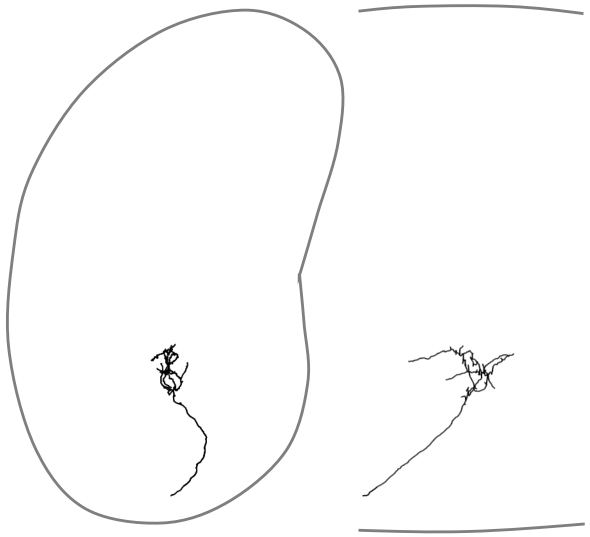

Mechanosensory 10 (lch) R

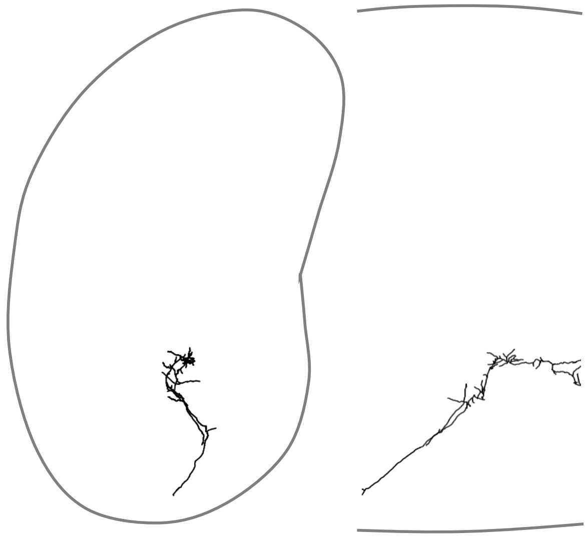

Mechanosensory 8 (vch) L

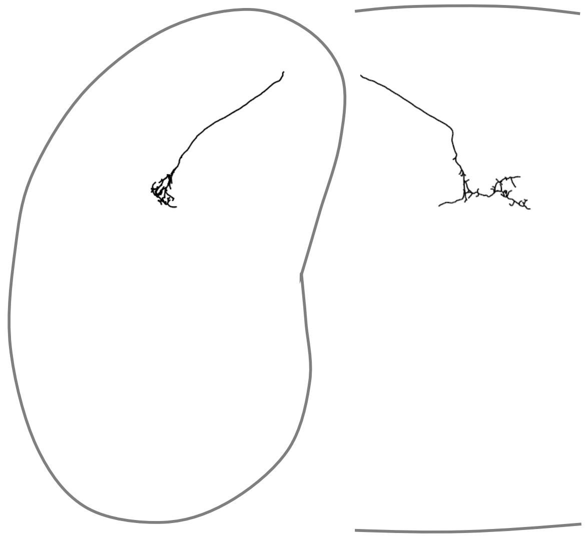

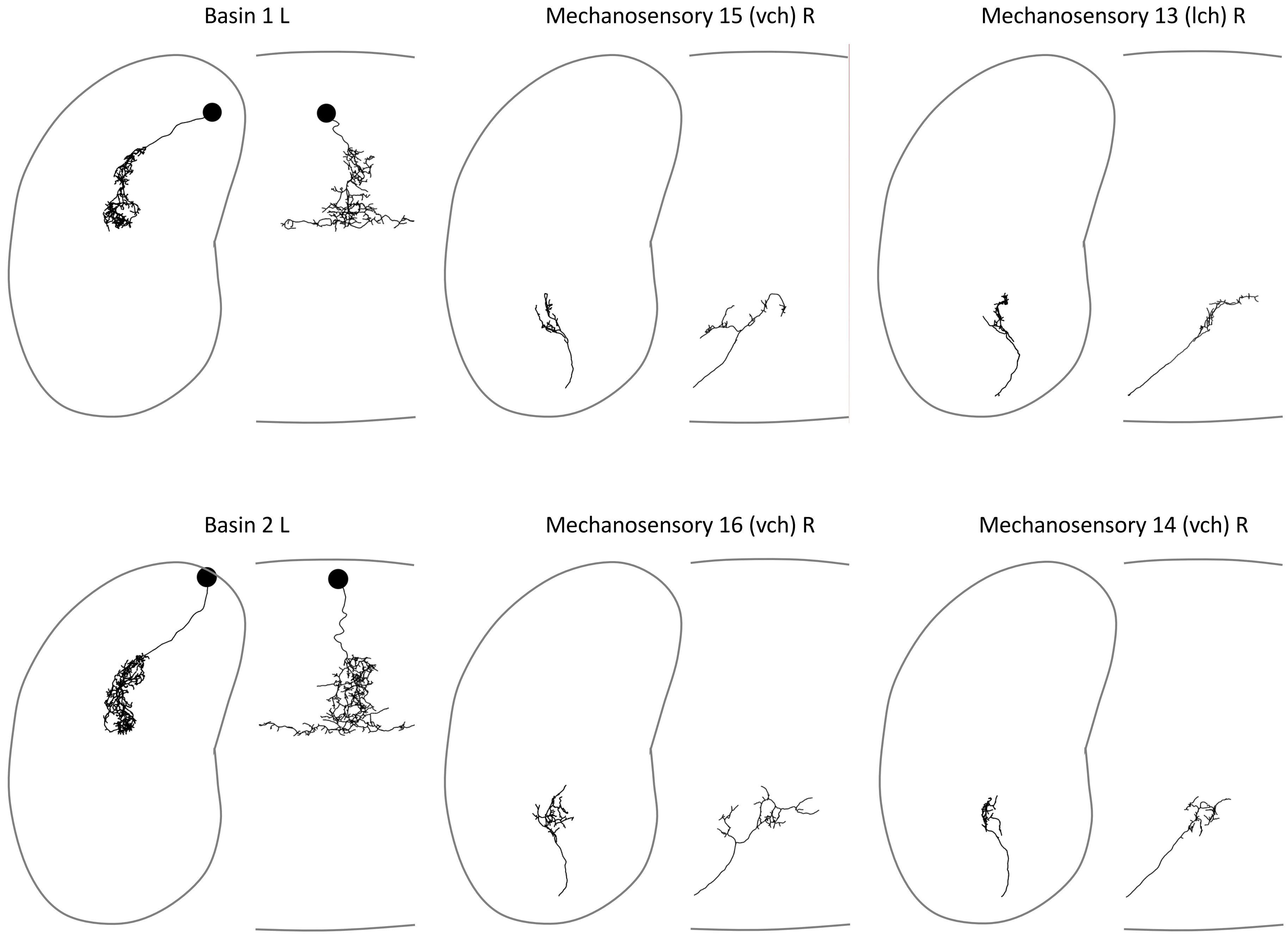
Basin 1 L
Mechanosensory 15 (vch) R
Mechanosensory 13 (lch) R
Basin 2 L
Mechanosensory 16 (vch) R
Mechanosensory 14 (vch) R

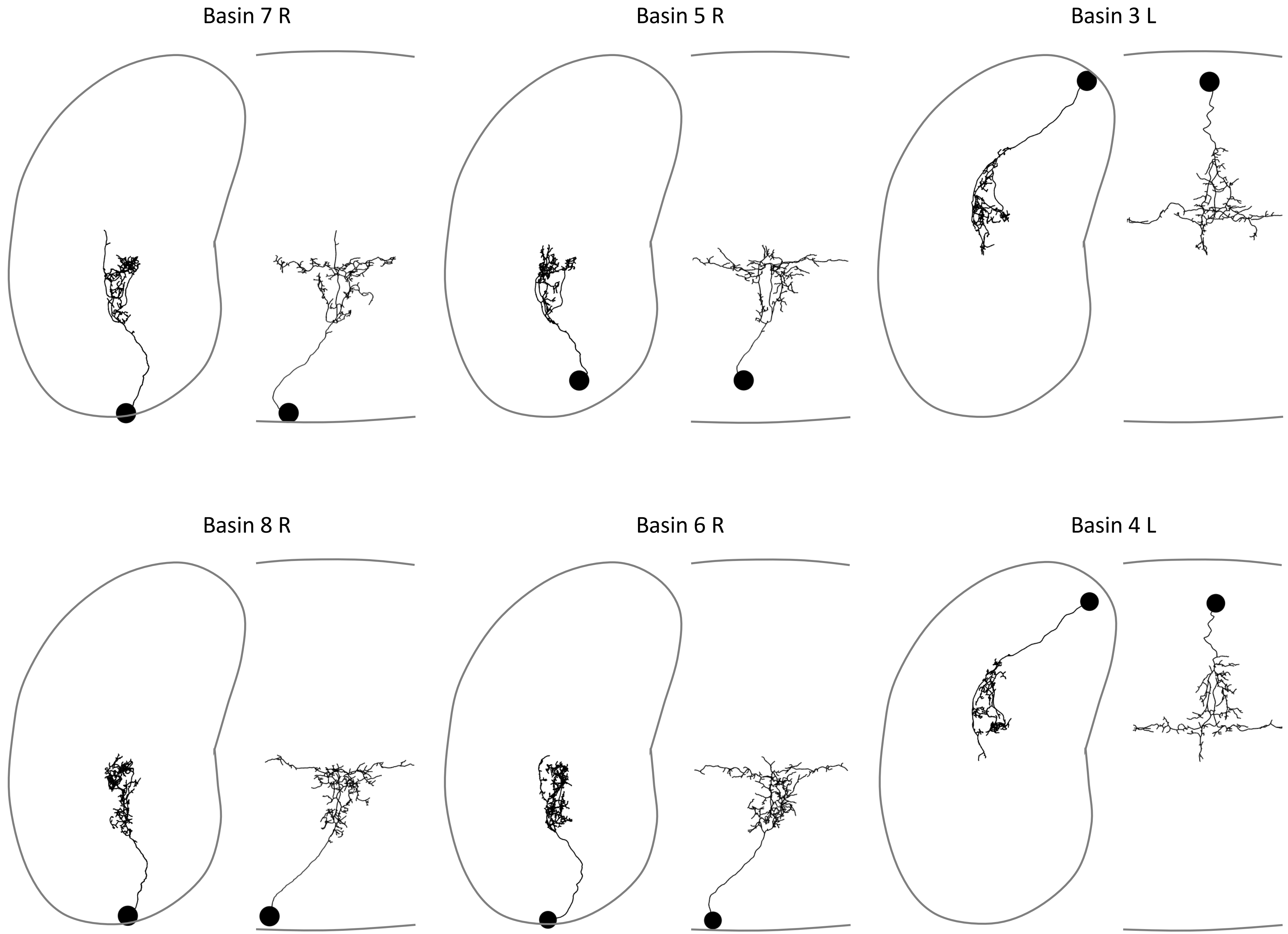

Basin 7 R
Basin 5 R
Basin 3 L
Basin 8 R
Basin 6 R
Basin 4 L

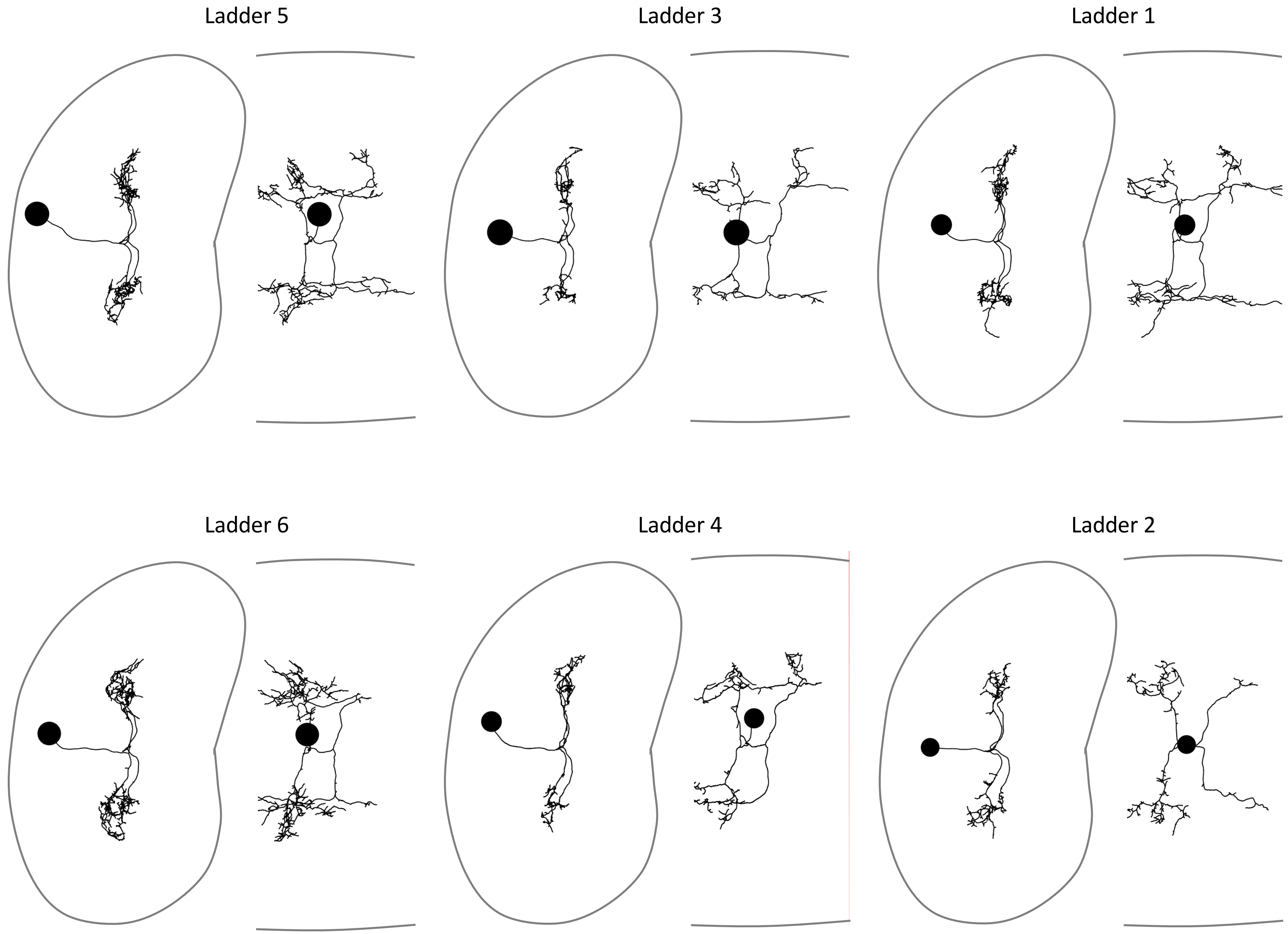
Ladder 5
Ladder 3
Ladder 1
Ladder 6
Ladder 4
Ladder 2

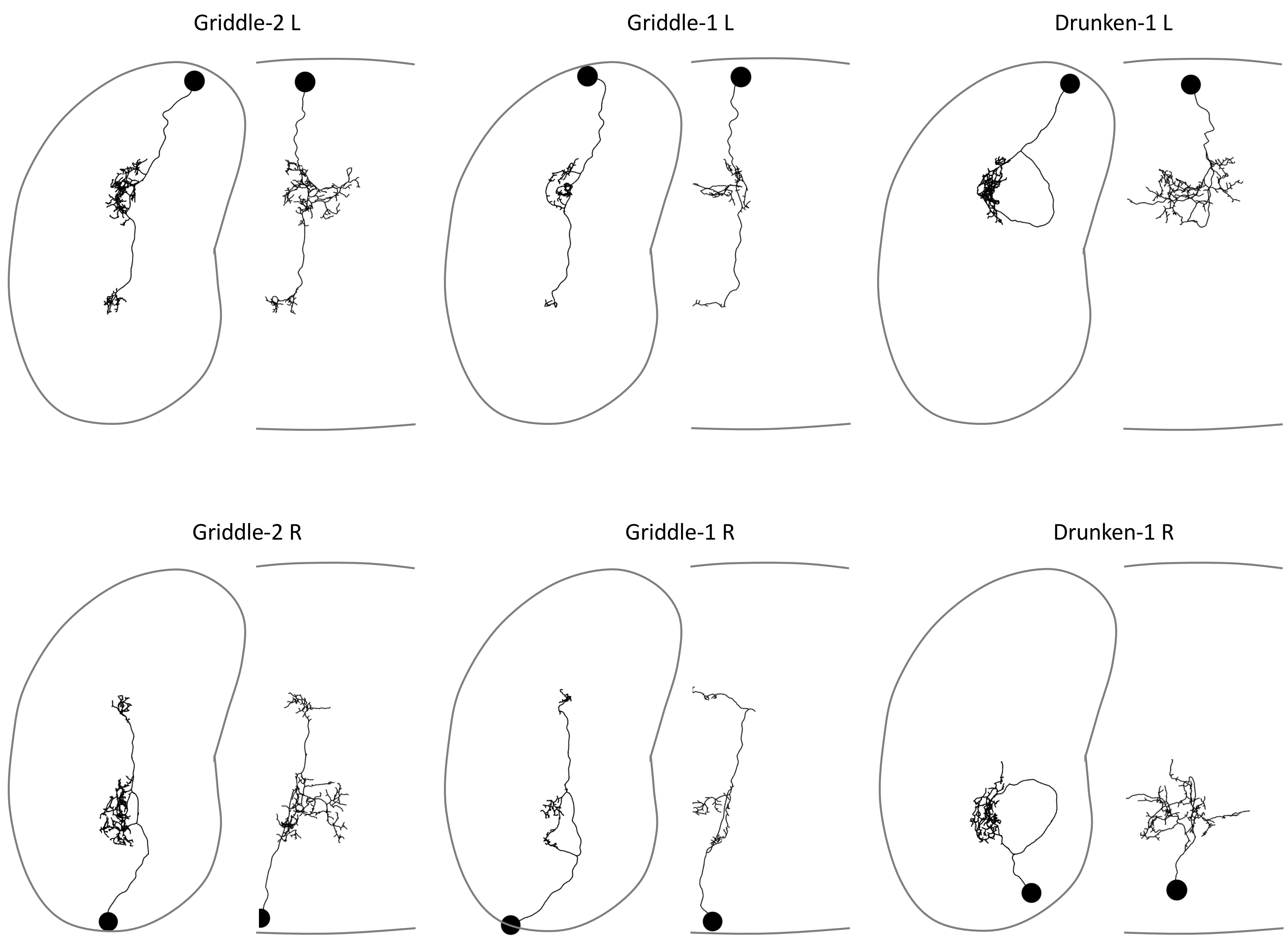
Griddle-2 L
Griddle-1 L
Drunken-1 L
Griddle-2 R
Griddle-1 R
Drunken-1 R

Data S2. Neuronal atlas of “other local” mechanosensory partners in the mechano>FraRobo and control EM volumes. Related to Figure 5B-C.

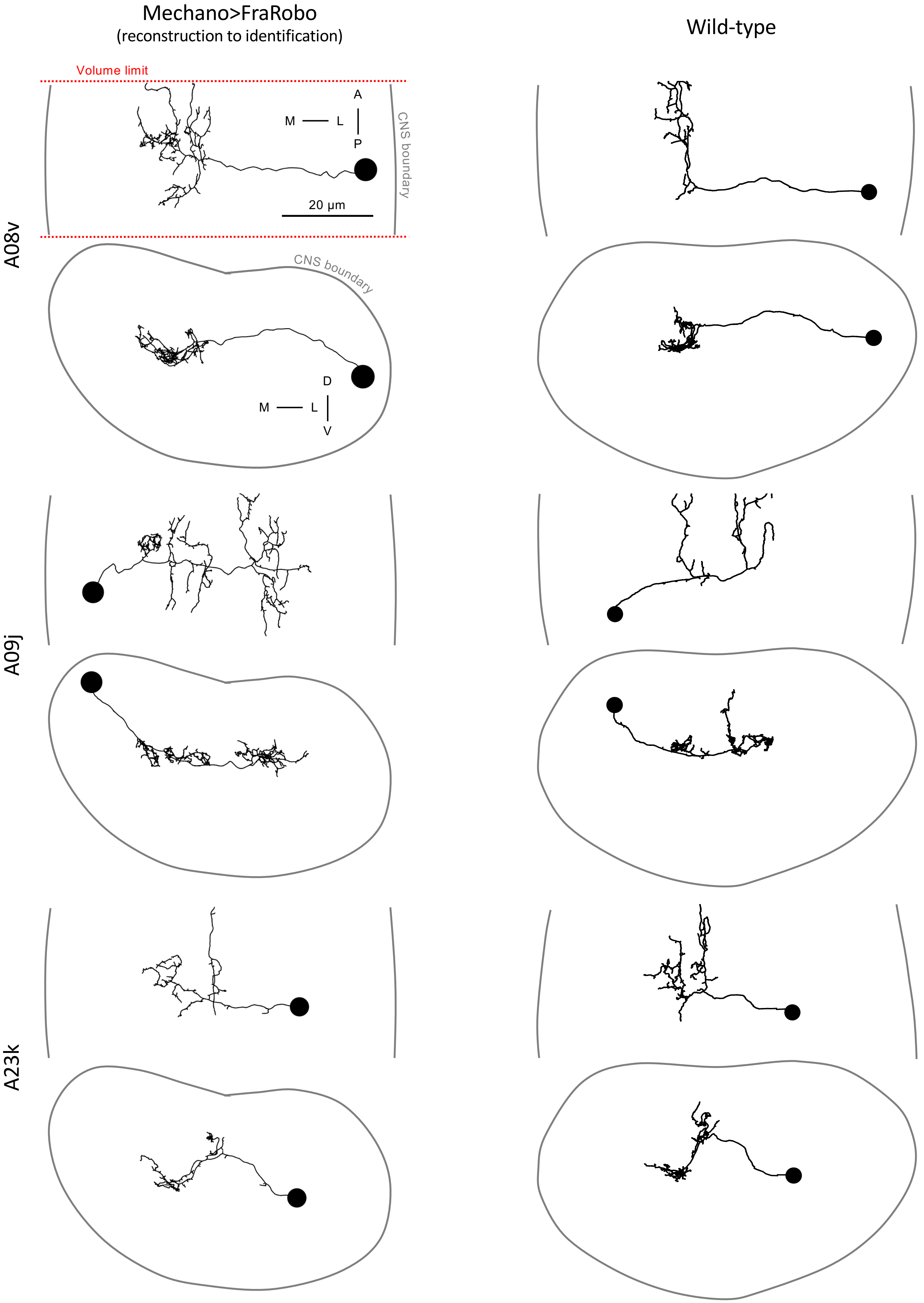

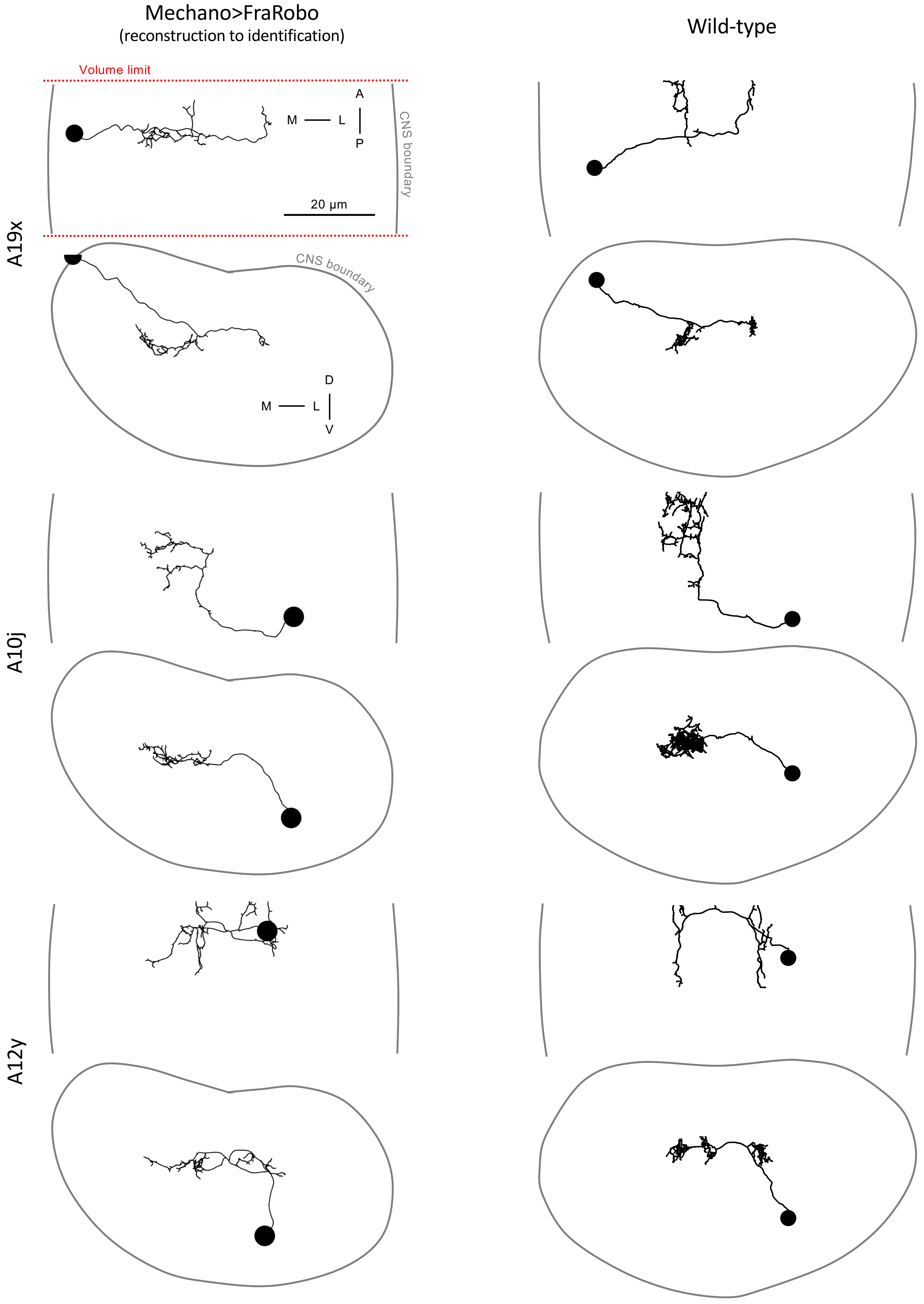

Mechano>FraRobo
(reconstruction to identification)
Wild-type
Volume limit
A
M
L
P
CNS boundary
20 μm
A19x
CNS boundary
D
M
L
V
A10j
A12y

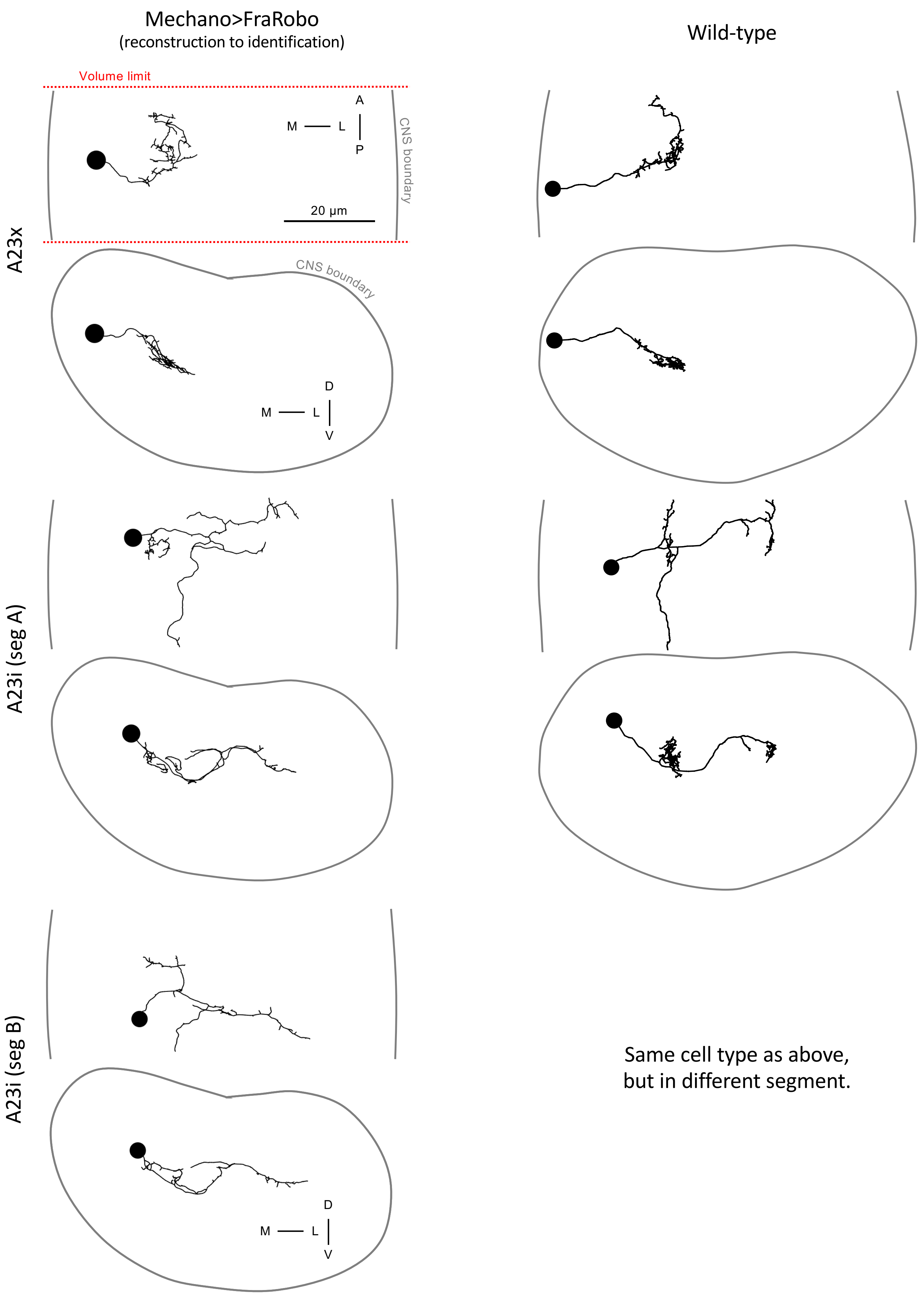
Mechano>FraRobo
(reconstruction to identification)
Wild-type
Volume limit
A
M
L
P
CNS boundary
20 µm
A23x
CNS boundary
D
M
L
V
A23i (seg A)
A23i (seg B)
D
M
L
V
Same cell type as above,
but in different segment.

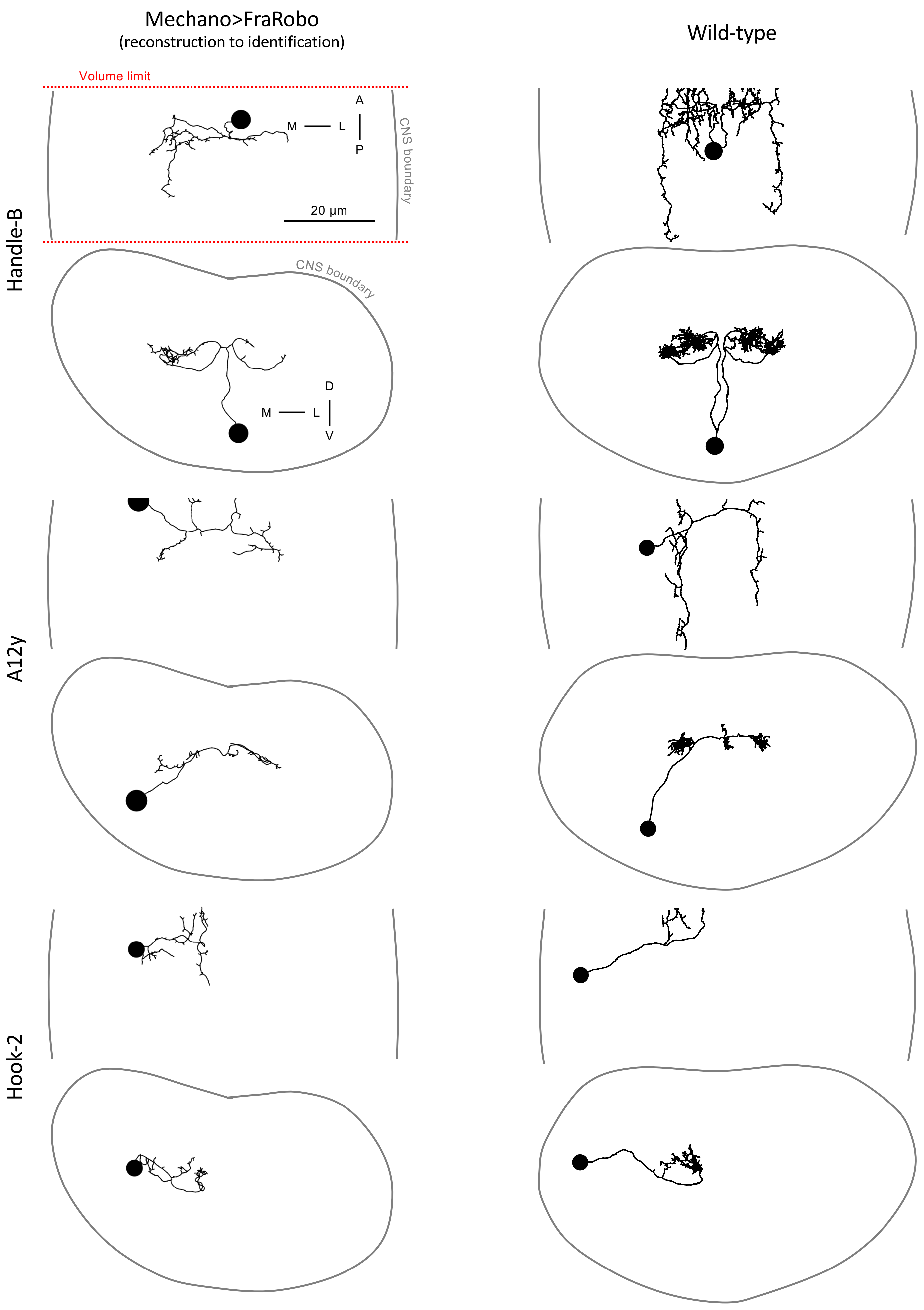

Mechano>FraRobo
(reconstruction to identification)
Wild-type
Volume limit
A
M
L
P
CNS boundary
20 µm
Handle-B
CNS boundary
D
M
L
V
A12y
Hook-2

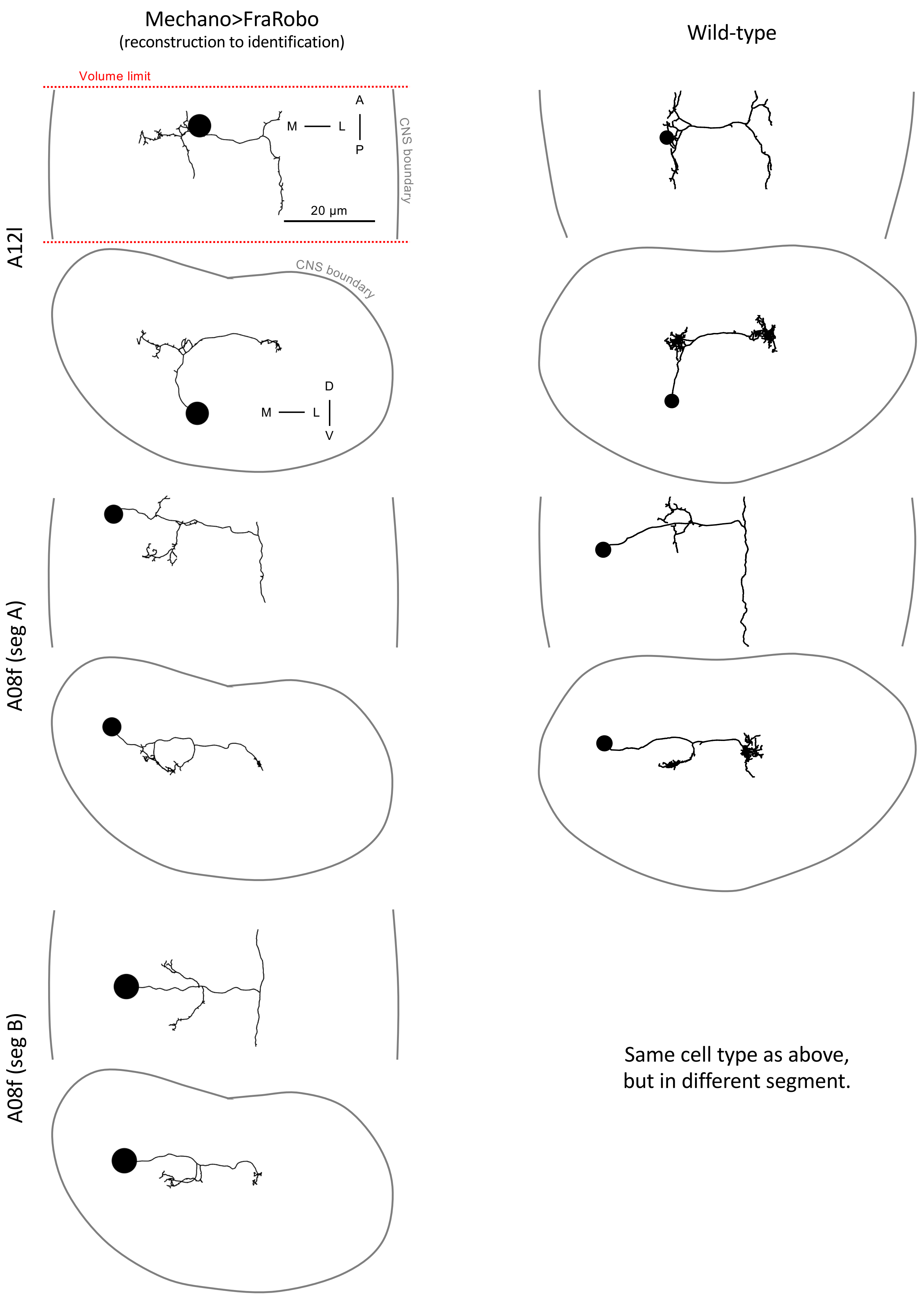

Mechano>FraRobo
(reconstruction to identification)
Wild-type
Volume limit
A
M
L
P
20 µm
CNS boundary
A12l
CNS boundary
D
M
L
V
A08f (seg A)
A08f (seg B)
Same cell type as above,
but in different segment.

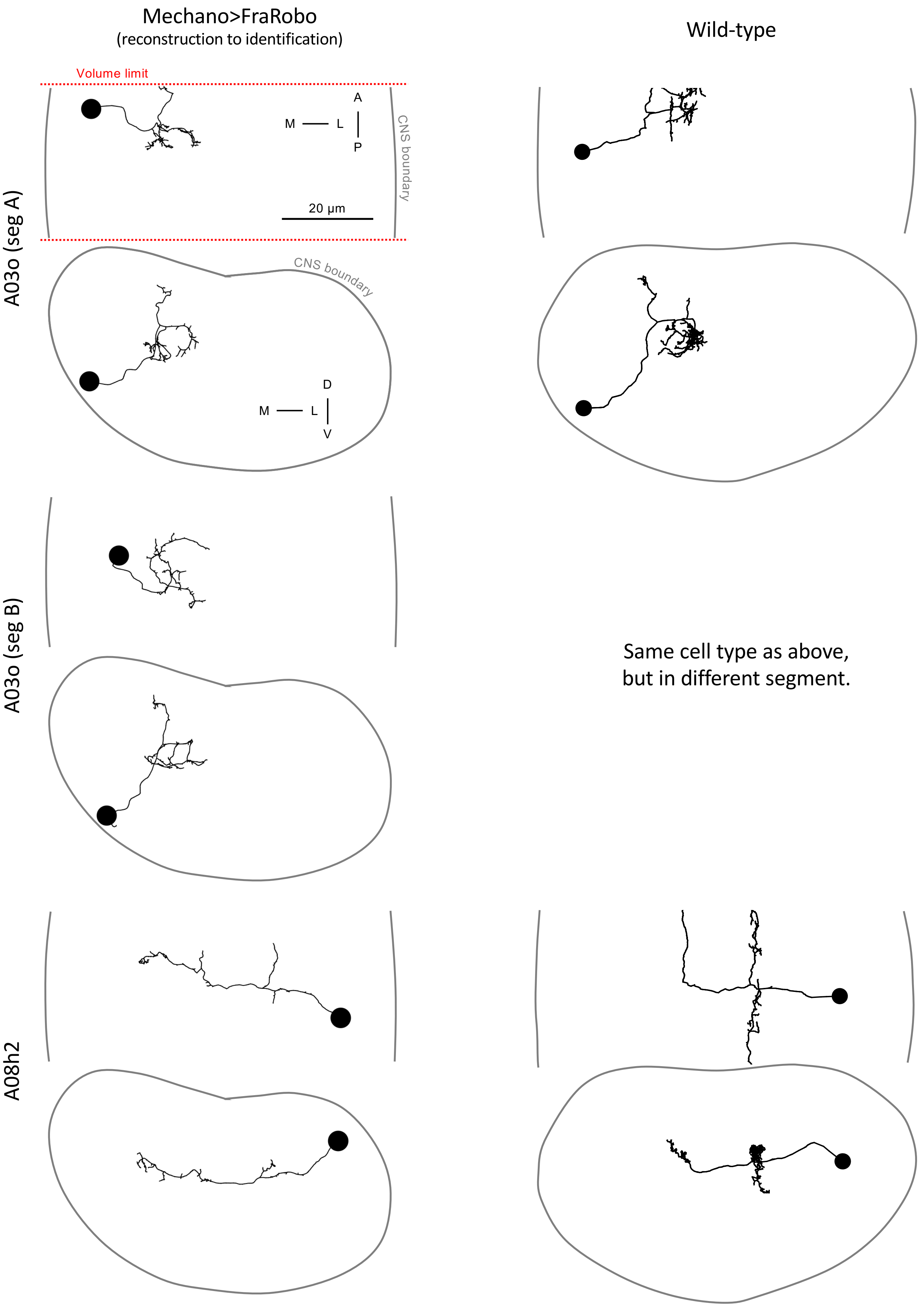
Mechano>FraRobo
(reconstruction to identification)
Wild-type
Volume limit
A
M
L
P
CNS boundary
20 µm
A03o (seg A)
CNS boundary
D
M
L
V
A03o (seg B)
Same cell type as above,
but in different segment.
A08h2

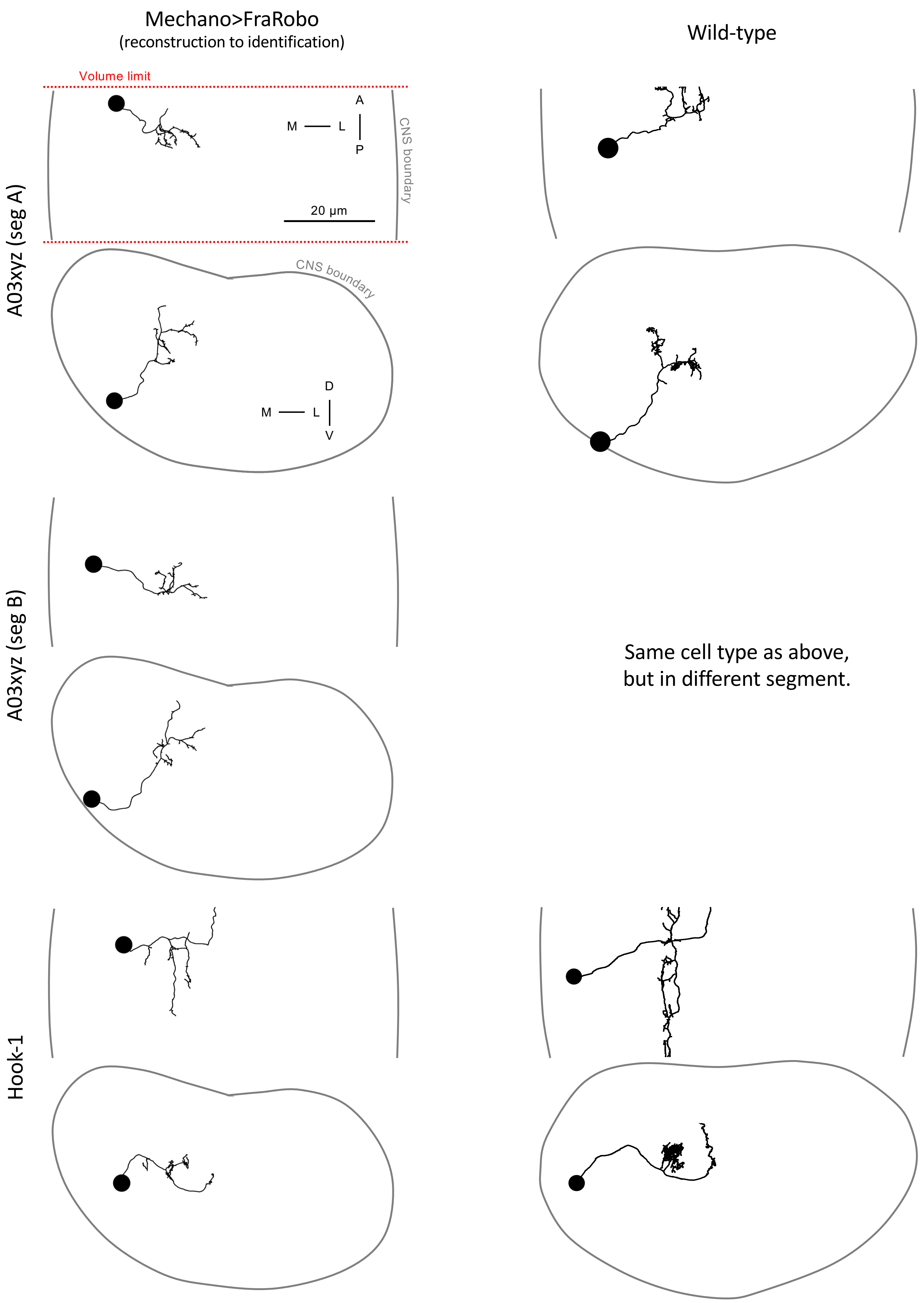
Mechano>FraRobo
(reconstruction to identification)
Wild-type
Volume limit
A
M
L
P
CNS boundary
20 µm
CNS boundary
D
M
L
V
A03xyz (seg A)
A03xyz (seg B)
Same cell type as above, but in different segment.
Hook-1

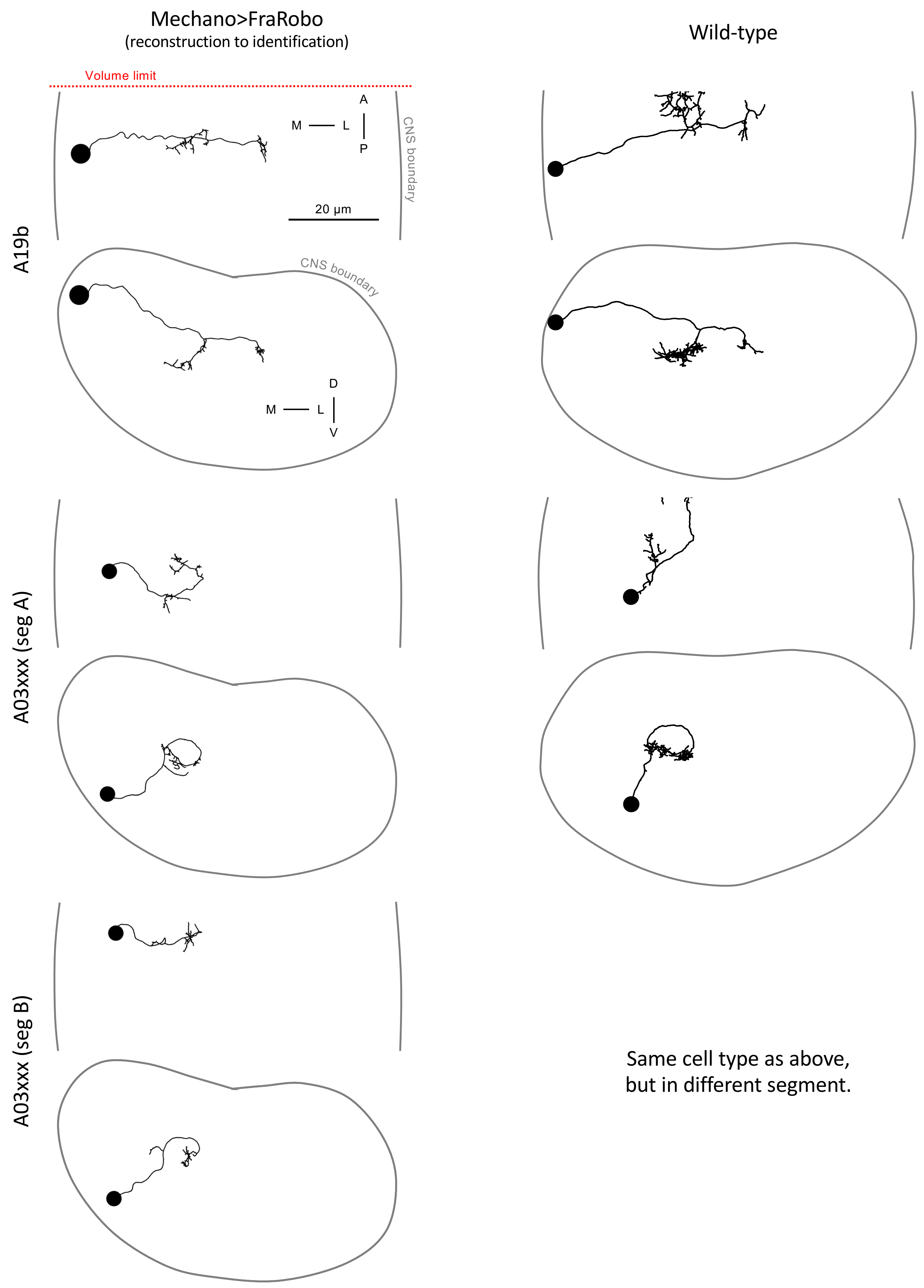

Mechano>FraRobo
(reconstruction to identification)
Wild-type
Volume limit
A
M
L
P
CNS boundary
20 µm
A19b
CNS boundary
D
M
L
V
A03xxx (seg A)
A03xxx (seg B)
Same cell type as above,
but in different segment.

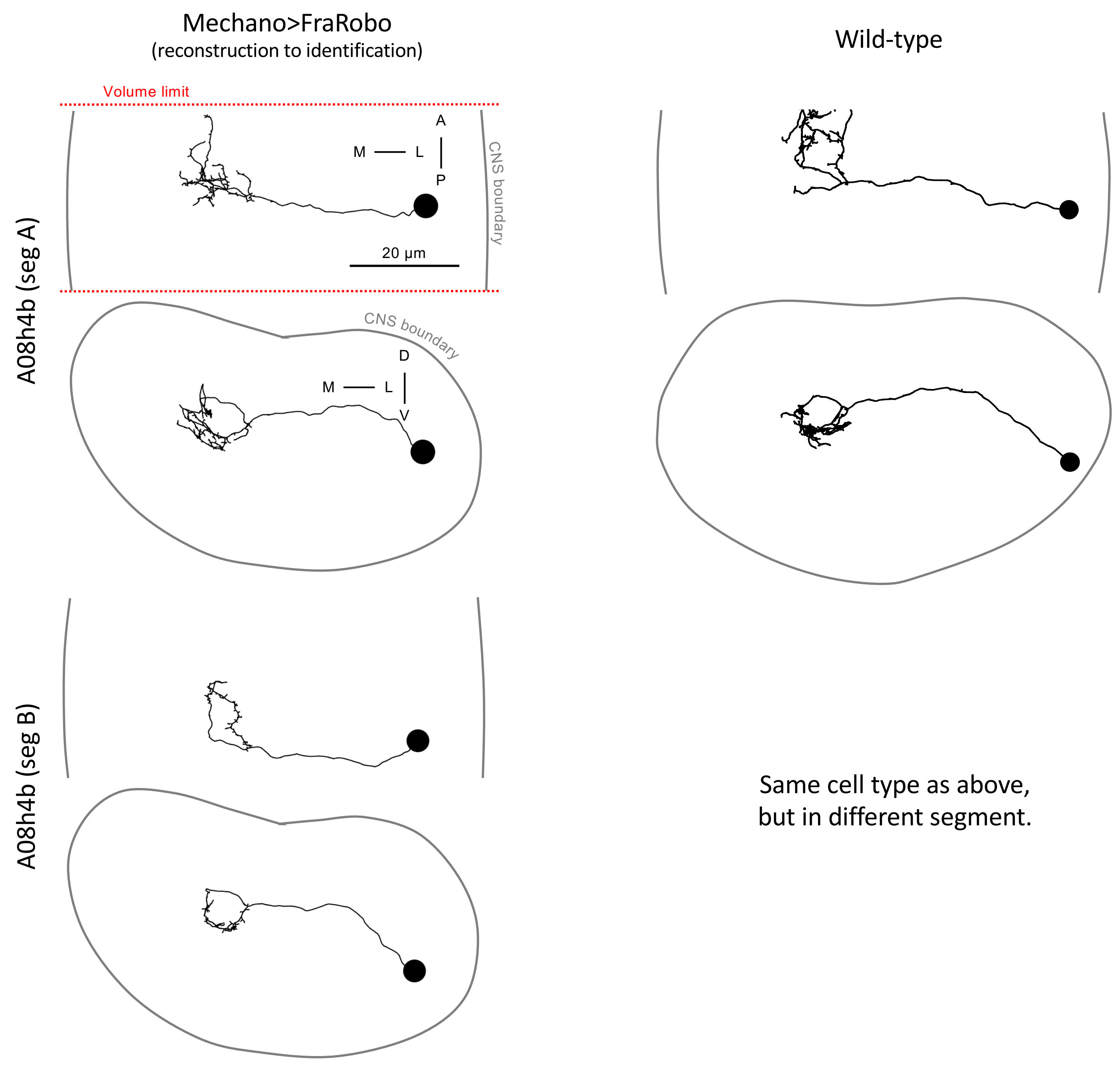

Mechano>FraRobo
(reconstruction to identification)
Wild-type
Volume limit
A
M
L
P
CNS boundary
20 µm
A08h4b (seg A)
CNS boundary
D
M
L
V
A08h4b (seg B)
Same cell type as above,
but in different segment.

Data S3. Neuronal atlas of mechanosensory neurons and preferred partners in the mechano>TNT EM volume. Related to Figures 6 and 7.

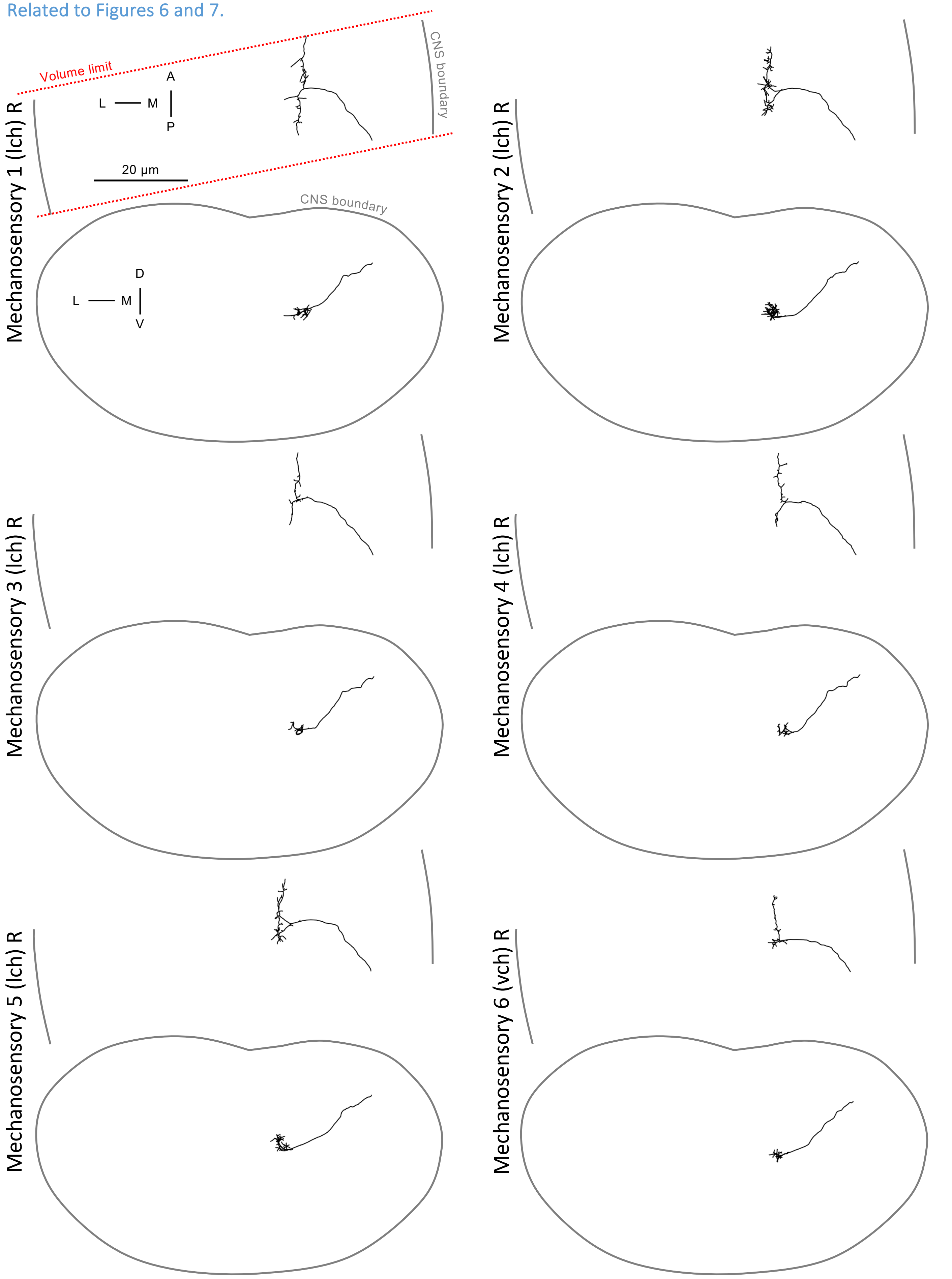

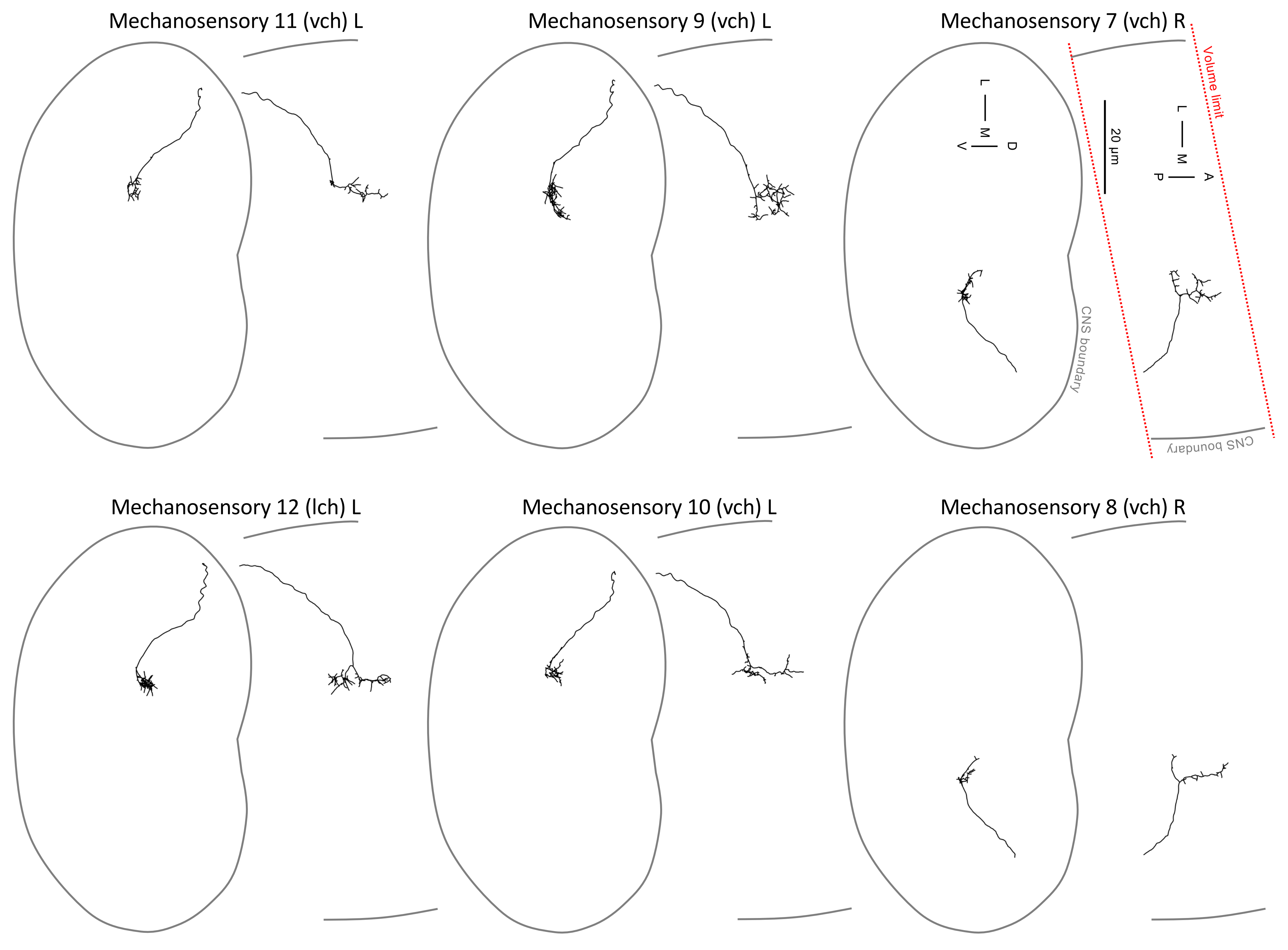

Mechanosensory 11 (vch) L
Mechanosensory 9 (vch) L
Mechanosensory 7 (vch) R
Volume limit
CNS boundary
CNS boundary
20 µm
L
M
A
P
L
M
D
V
Mechanosensory 12 (lch) L
Mechanosensory 10 (vch) L
Mechanosensory 8 (vch) R

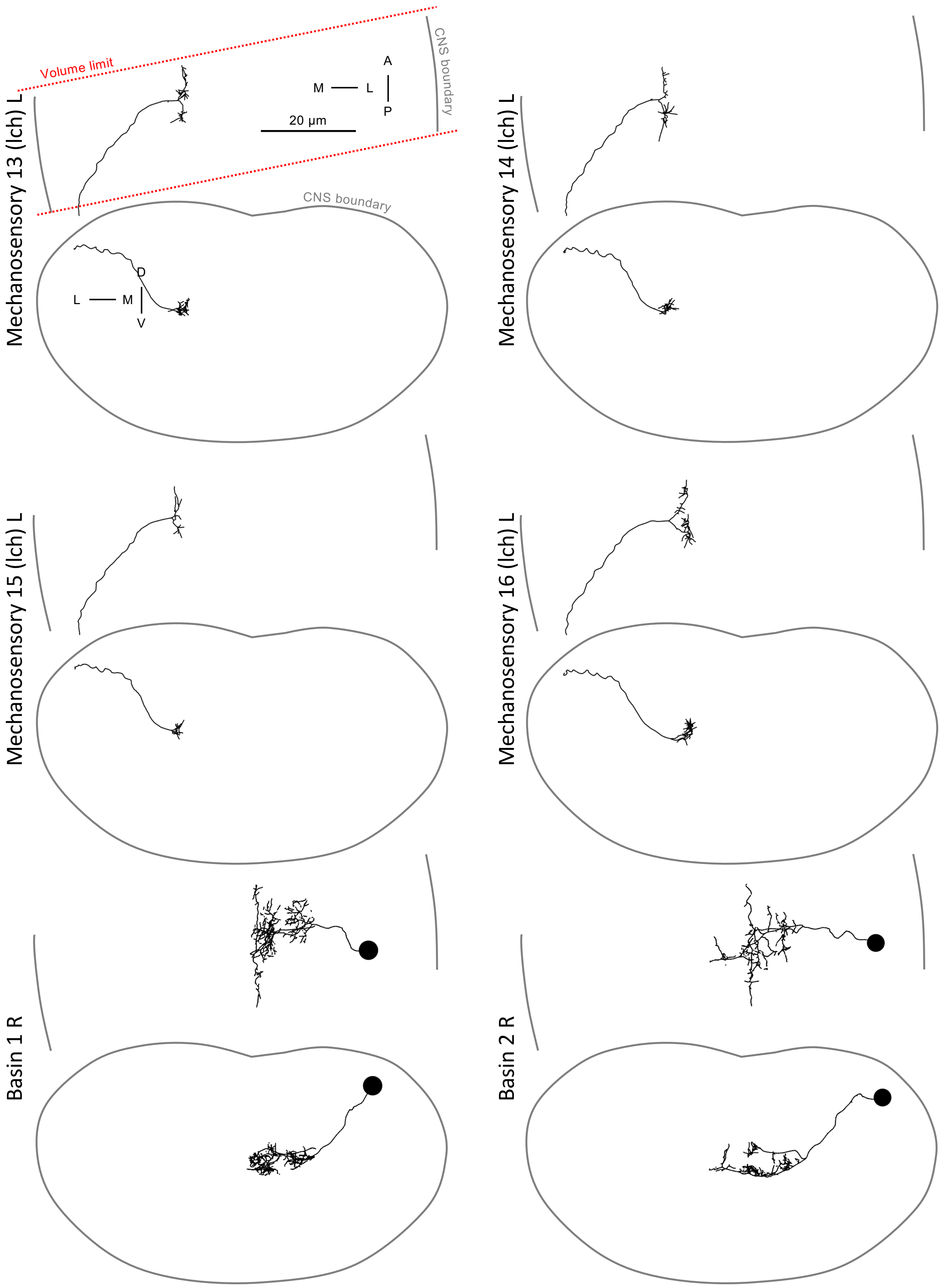
Mechanosensory 13 (lch) L
Volume limit
CNS boundary
A
M
L
P
20 µm
CNS boundary
D
L
M
V
Mechanosensory 14 (lch) L
Mechanosensory 15 (lch) L
Mechanosensory 16 (lch) L
Basin 1 R
Basin 2 R

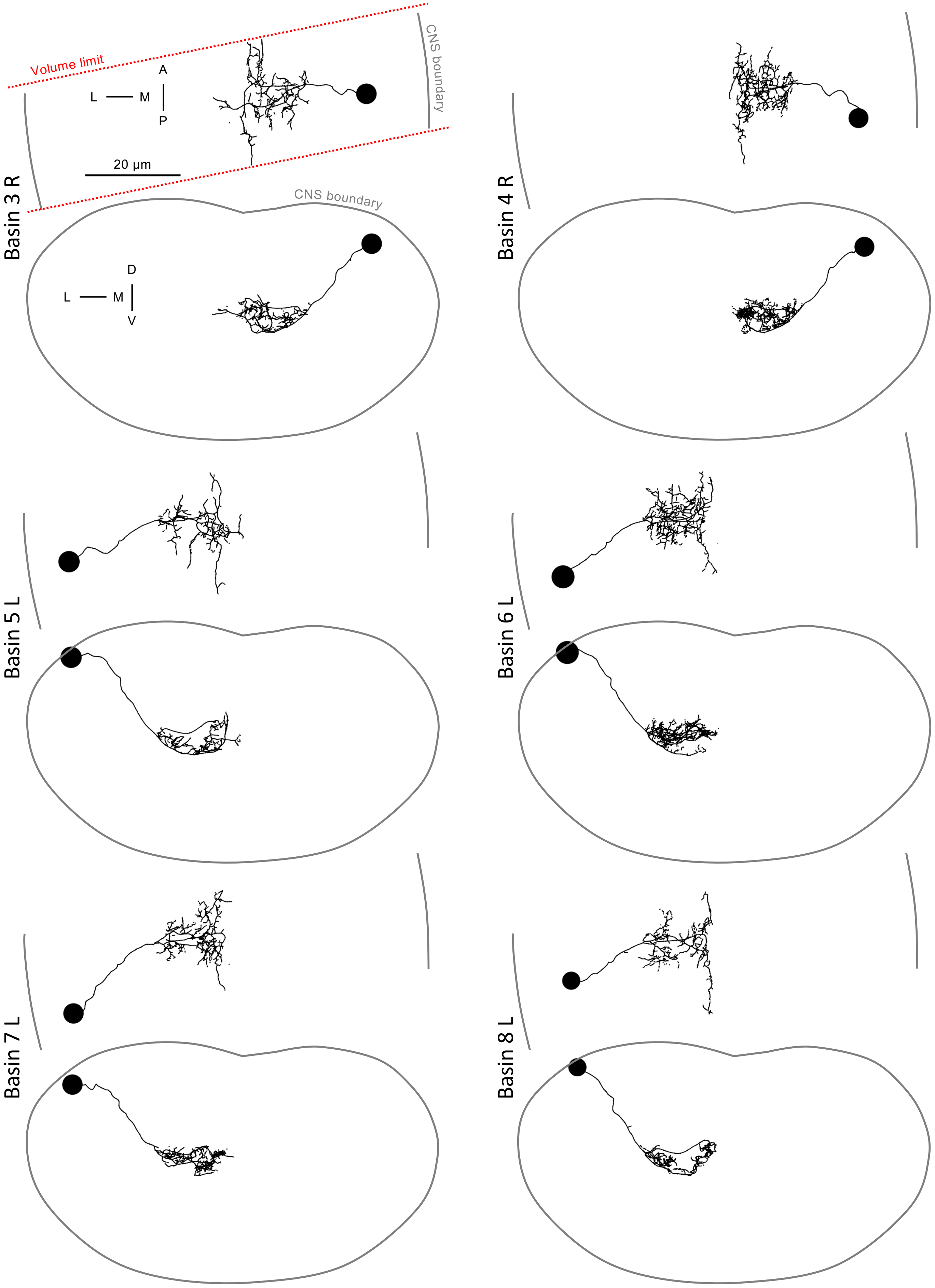
Volume limit
CNS boundary
A
L
M
P
20 µm
CNS boundary
D
L
M
V
Basin 3 R
Basin 4 R
Basin 5 L
Basin 6 L
Basin 7 L
Basin 8 L

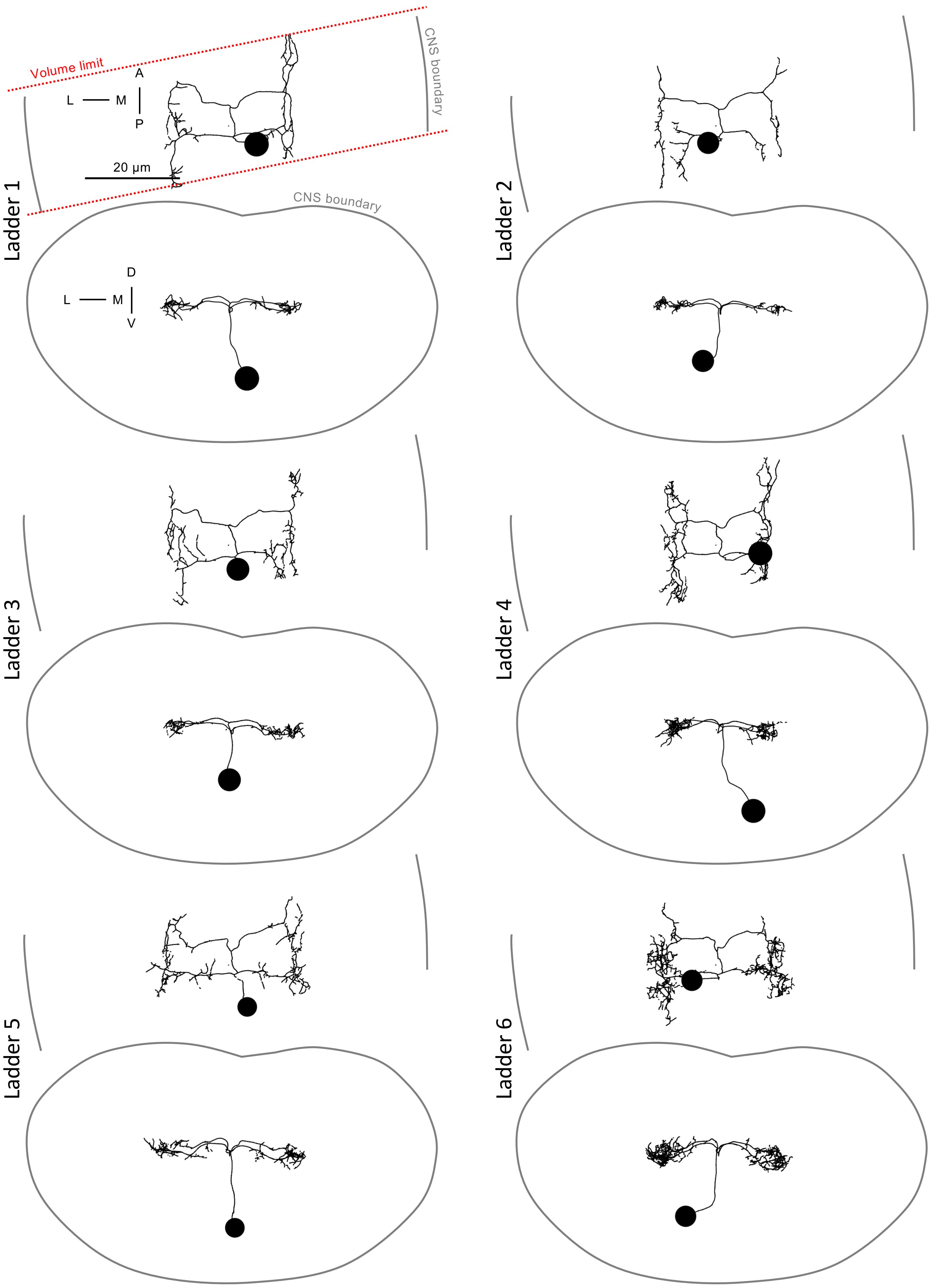

Volume limit
A
L
M
P
20 µm
CNS boundary
CNS boundary
D
L
M
V
Ladder 1
Ladder 2
Ladder 3
Ladder 4
Ladder 5
Ladder 6

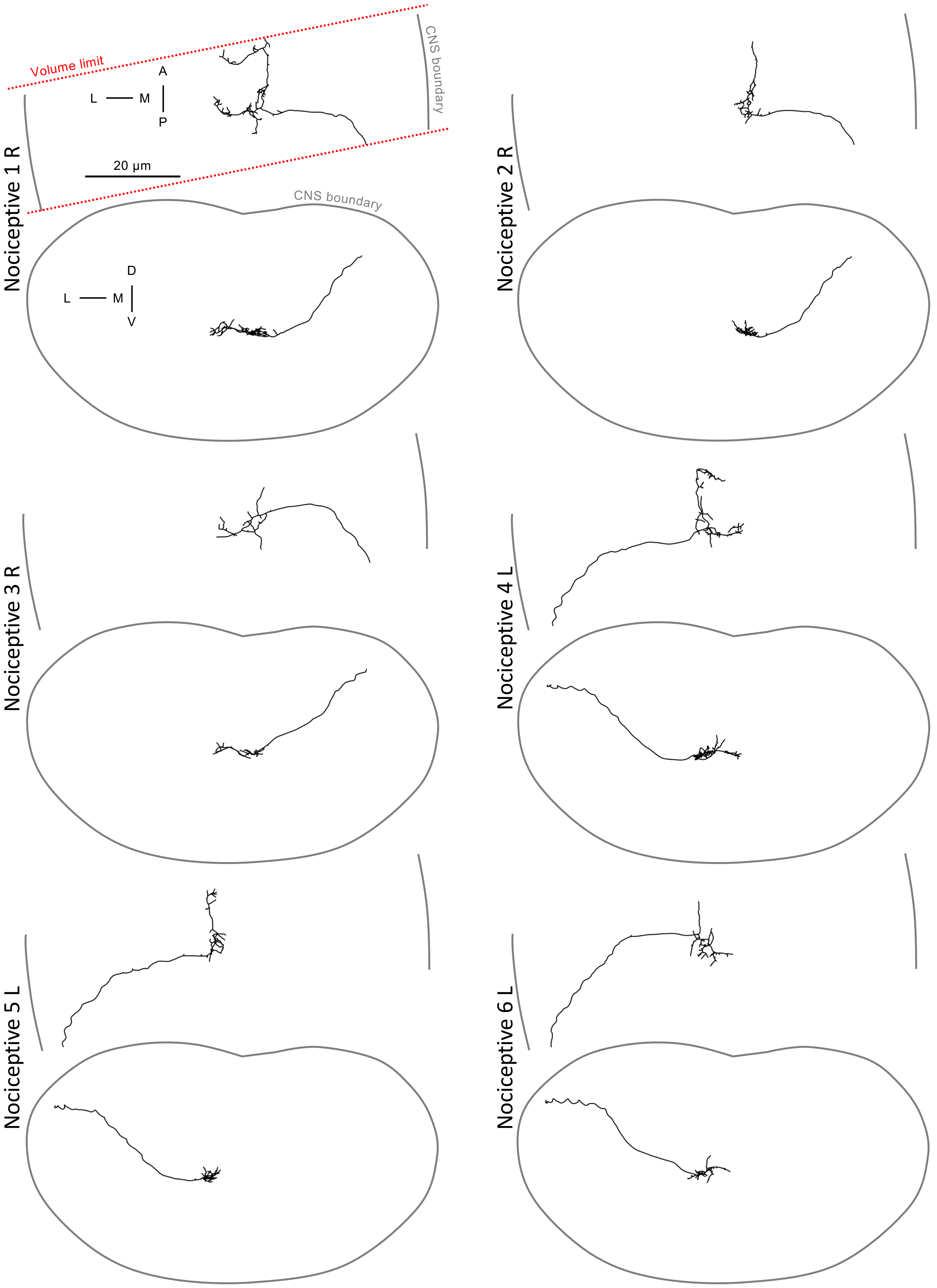

Volume limit
CNS boundary
CNS boundary
A
L
M
P
20 µm
D
L
M
V
Nociceptive 1 R
Nociceptive 2 R
Nociceptive 3 R
Nociceptive 4 L
Nociceptive 5 L
Nociceptive 6 L

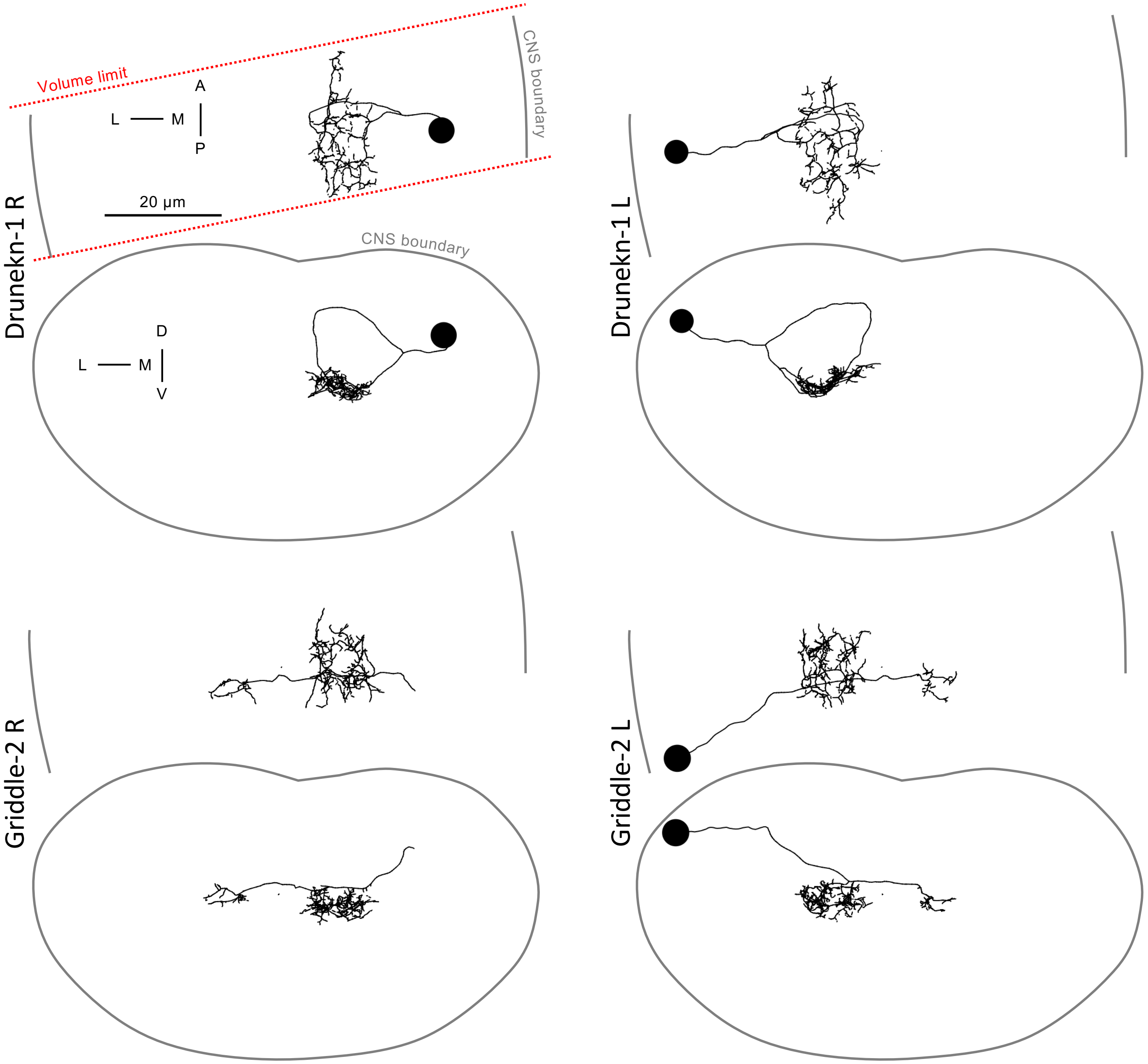

Volume limit
CNS boundary
A
L
M
P
20 µm
Drunekn-1 R
CNS boundary
D
L
M
V
Drunekn-1 L
Griddle-2 R
Griddle-2 L
