# Supplementary material for: Comparative Connectomics Reveals How Partner Identity, Location, and Activity Specify Synaptic Connectivity in Drosophila
Source: Neuron. 2021 Jan 6;109(1):105–122.e7. doi: 10.1016/j.neuron.2020.10.004 (PMC7837116; doi:10.1016/j.neuron.2020.10.004)
Supplement: Document S2. Article plus Supplemental Information [file mmc3.pdf]

Neuron

Article

Comparative Connectomics Reveals How Partner Identity, Location, and Activity Specify Synaptic Connectivity in *Drosophila*

Highlights

- Synaptic partners manage to find and connect with each other in aberrant locations
- Partner recognition alone does not ensure proper circuit formation and function
- Appropriate location and partner identity are required for proper synapse numbers
- Developmental activity affects balance of excitation and inhibition in the circuit

Authors

Javier Valdes-Aleman, Richard D. Fetter, Emily C. Sales, ..., Matthias Landgraf, Albert Cardona, Marta Zlatic

Correspondence

mzlatic@mrc-lmb.cam.ac.uk

In Brief

Valdes-Aleman et al. show that neurons manage to find and connect to their partners even at abnormal locations or when silenced. However, inappropriate numbers of connections are established, disrupting the balance of excitation and inhibition and generating deficient behavior. Neuron location, identity, and developmental activity work together to control circuit assembly.

Valdes-Aleman et al., 2021, Neuron *109*, 105–122
January 6, 2021 Crown Copyright © 2020 Published by Elsevier Inc.
https://doi.org/10.1016/j.neuron.2020.10.004

CellPress

Neuron

CellPress
OPEN ACCESS

Article

Comparative Connectomics Reveals How Partner Identity, Location, and Activity Specify Synaptic Connectivity in *Drosophila*

Javier Valdes-Aleman,[1,2] Richard D. Fetter,[1,6] Emily C. Sales,[3] Emily L. Heckman,[3] Lalanti Venkatasubramanian,[2] Chris Q. Doe,[3] Matthias Landgraf,[2] Albert Cardona,[1,4,5] and Marta Zlatic[1,2,5,7,*]
[1]Janelia Research Campus, Howard Hughes Medical Institute, 19700 Helix Drive, Ashburn, VA 20147, USA
[2]Department of Zoology, University of Cambridge, Downing Street, Cambridge CB2 3EJ, UK
[3]Institute of Neuroscience, Howard Hughes Medical Institute, University of Oregon, Eugene, OR 97403, USA
[4]Department of Physiology, Development and Neuroscience, University of Cambridge, Downing Street, Cambridge CB2 3EG, UK
[5]MRC Laboratory of Molecular Biology, Cambridge Biomedical Campus, Francis Crick Avenue, Cambridge CB2 0QH, UK
[6]Present address: Department of Biology, Howard Hughes Medical Institute, Stanford University, Stanford, CA 94305, USA
[7]Lead Contact
*Correspondence: mzlatic@mrc-lmb.cam.ac.uk
https://doi.org/10.1016/j.neuron.2020.10.004

SUMMARY

The mechanisms by which synaptic partners recognize each other and establish appropriate numbers of connections during embryonic development to form functional neural circuits are poorly understood. We combined electron microscopy reconstruction, functional imaging of neural activity, and behavioral experiments to elucidate the roles of (1) partner identity, (2) location, and (3) activity in circuit assembly in the embryonic nerve cord of *Drosophila*. We found that postsynaptic partners are able to find and connect to their presynaptic partners even when these have been shifted to ectopic locations or silenced. However, orderly positioning of axon terminals by positional cues and synaptic activity is required for appropriate numbers of connections between specific partners, for appropriate balance between excitatory and inhibitory connections, and for appropriate functional connectivity and behavior. Our study reveals with unprecedented resolution the fine connectivity effects of multiple factors that work together to control the assembly of neural circuits.

INTRODUCTION

Our nervous system is organized into circuits with specifically matched and tuned cell-to-cell connections essential for proper function. During development, neurons navigate through the nervous system to reach their target location (Araújo and Tear, 2003; Dickson, 2002; Kolodkin and Tessier-Lavigne, 2011; Tessier-Lavigne and Goodman, 1996; Yogev and Shen, 2014). Surrounded by numerous cells along their trajectories and in their target areas, developing neurons ignore most cells and connect only to specific partners (Eichler et al., 2017; Gerhard et al., 2017; Helmstaedter et al., 2013; Jovanic et al., 2016; Lee et al., 2016; Ohyama et al., 2015; Schneider-Mizell et al., 2016; Takemura et al., 2013, 2015, 2017; White et al., 1986; Zheng et al., 2018).

The absolute numbers of synapses between specific partners can vary across individuals, hemispheres, or repeated network modules in the same individual (Bartol et al., 2015; Eichler et al., 2017; Gerhard et al., 2017; Goodman, 1978; Hamood and Marder, 2015; Jovanic et al., 2016; Lu et al., 2009; Ohyama et al., 2015; Ryan et al., 2016; Schneider-Mizell et al., 2016; Takemura et al., 2015; Tobin et al., 2017; Ward et al., 1975). However, recent electron microscopy (EM) reconstructions in multiple *Drosophila* larvae suggest that, at least in some circuits, the relative numbers of synapses between partners are precisely regulated (Eichler et al., 2017; Gerhard et al., 2017; Jovanic et al., 2016; Ohyama et al., 2015; Takemura et al., 2017). Thus, the fraction of inputs a neuron receives from a specific partner, relative to its total number of inputs, is remarkably conserved across individuals (Jovanic et al., 2016; Ohyama et al., 2015; Schneider-Mizell et al., 2016; Zarin et al., 2019), across larval stages (Gerhard et al., 2017), and even between larva and adult (Eichler et al., 2017; Takemura et al., 2017). For example, the fraction of input varied by an average factor (fold change; i.e., the ratio of two fractions) of 1.07 ± 0.22 between different first instar larvae (n = 13 homologous connections) and 1.09 ± 0.20 from first to third instar (n = 12 homologous connections; Gerhard et al., 2017). Similarly, the average input a mushroom body output neuron receives from a modulatory neuron in the larva and adult is 3.4% and 3.3%, respectively (Eichler et al., 2017; Takemura et al., 2017). These examples of conserved fractions of synaptic input across individuals and life stages raise several key questions: (1) How important are the precise numbers of

Crown Copyright © 2020 Published by Elsevier Inc.
This is an open access article under the CC BY license (http://creativecommons.org/licenses/by/4.0/).

connections between neurons for normal behavior? (2) How are the precise numbers of connections between partners specified? and (3) How is the appropriate balance between excitatory and inhibitory connections in the circuit achieved?

The chemoaffinity hypothesis proposes that pre- and postsynaptic partners express specific matching combinations of cell-surface molecules that enable them to seek out and recognize each other during development (Langley, 1895; Sperry, 1963). However, relatively few examples of partner-recognition molecules have been identified (Hong and Luo, 2014; Hong et al., 2012; Krishnaswamy et al., 2015; Sanes and Yamagata, 2009; Ward et al., 2015; Xu et al., 2018), so it is unclear whether their use is a general principle or if they are used only in some systems. It is also unknown if these partner-recognition mechanisms specify precise numbers of synapses between partners, or only instruct two neurons to form synapses, but not how many.

Alternative hypotheses propose that neurons seek out specific locations in the nervous system, rather than specific partners, indiscriminately connecting to whichever neurons are present there (Peters and Feldman, 1976; Rees et al., 2017). Consistent with this, neurons have been shown to use non-partner-derived positional cues, such as third-party guidepost cells (Shen and Bargmann, 2003; Shen et al., 2004) or gradients of repellents, to select their termination and synaptogenesis area independently of their partners (Couton et al., 2015; Fukuhara et al., 2013; Mauss et al., 2009; Sürmeli et al., 2011; Zlatic et al., 2003, 2009). Additionally, activity-dependent mechanisms are thought to refine connections through Hebbian and/or homeostatic plasticity mechanisms (Giachello and Baines, 2015; Kaneko et al., 2017; Marder, 2011; Schulz and Lane, 2017; Sheng et al., 2018; Sugie et al., 2018; Tien and Kerschensteiner, 2018; Tripodi et al., 2008; Turrigiano, 2017; Yuan et al., 2011). Neurons that fire together preferentially wire together in many areas of the vertebrate nervous system through positive feedback (Abbott and Nelson, 2000; Brown et al., 2009; Malenka and Bear, 2004). At the same time, homeostatic mechanisms restore activity toward a specific set point through negative feedback, imposing competition and preventing runaway excitation or complete silencing of the circuit (Burrone and Murthy, 2003; Kilman et al., 2002; Maffei and Turrigiano, 2008; Marder, 2011; Marder and Goaillard, 2006; Rutherford et al., 1998; Tripodi et al., 2008; Turrigiano, 2017; Turrigiano and Nelson, 2004). However, the extent to which activity modulates numbers versus the strength of existing synapses is still an open question.

These questions have been difficult to address because they require manipulating candidate factors that could influence connectivity, visualizing synapses between uniquely identified partners, and relating observed structural changes to effects on functional connectivity and behavior. We therefore used the tractable *Drosophila* larva as a model system with the following advantages: (1) excellent genetic tools for selective manipulation of uniquely identified neurons (Jenett et al., 2012; Pfeiffer et al., 2008, 2010; Venken et al., 2011); (2) a compact nervous system amenable to rapid imaging with synaptic resolution (Gerhard et al., 2017; Helmstaedter et al., 2011; Jovanic et al., 2016; Ohyama et al., 2015); and (3) a rich behavioral repertoire with well-established quantitative assays (Gomez-Marin et al., 2011; Hwang et al., 2007; Kane et al., 2013; Klein et al., 2015; Louis and de Polavieja, 2017; Luo et al., 2010; Ohyama et al., 2013; Robertson et al., 2013; Vogelstein et al., 2014; Xiang et al., 2010; Zhang et al., 2013).

Recently, comprehensive synaptic-resolution connectivity maps of the circuitry downstream of the mechanosensory Chordotonal (hereafter "mechanosensory") neurons and nociceptive multidendritic class IV (hereafter "nociceptive") neurons in an abdominal segment of a first instar larva (Figures 1A–1C) have been generated (Jovanic et al., 2016; Ohyama et al., 2015). Portions of this circuit were also reconstructed in two different abdominal segments (A1 and A3) of two different first instar individuals (Jovanic et al., 2016; Ohyama et al., 2015) and at two different life stages: first (A1) and third instar (A3; Gerhard et al., 2017; Ohyama et al., 2015).

Here, we selectively altered the location or activity of the mechanosensory neurons and generated new EM volumes of the manipulated samples to investigate the effects on connectivity. We complemented these anatomical studies with functional connectivity and behavioral assays. Our study reveals that proper location, partner identity, and activity are all required to achieve appropriate connectivity and behavior.

RESULTS

Postsynaptic Partners Perform Extensive Exploration during Development

In the embryonic *Drosophila* ventral nerve cord (VNC), somatosensory axons use positional cues to select where to terminate, branch, and establish synaptic connections, independently of their partners (Zlatic et al., 2003, 2009). Dendrites actively explore during circuit formation in some systems (Mumm et al., 2006; Niell et al., 2004), but this has not been investigated in the *Drosophila* somatosensory circuit. Furthermore, whether partner dendrites use the same positional cues to independently terminate in the same area as their presynaptic somatosensory axons or whether they seek out specific presynaptic axons is unknown. To determine the extent of axonal and dendritic exploration, we performed live imaging in the intact embryo to follow the development of mechanosensory neurons and postsynaptic Basin interneurons (Figure 2A; Video S1).

The earliest Basin morphology detected ($\sim$13 h after egg laying [AEL]) consists of a bare primary branch projecting from the cell body toward the midline (Figure 2B). Short-lived dendritic filopodia grow from the middle of the primary branch, while axonal filopodia grow from the growth cone at the medial end. By the end of development, dendritic filopodia had explored most of the mediolateral and anteroposterior axes of their hemisegment.

The mechanosensory axonal growth cones were first detected already at the anteroposterior tract they normally occupy in the VNC (Figure 2B). These immature axons proceed to extend exploratory filopodia, as they project anteriorly and posteriorly. Interestingly, the mechanosensory axons target the correct anteroposterior tract even before Basin dendritic filopodia initiate exploration, supporting the idea that this axonal targeting is independent of postsynaptic partners (Zlatic et al., 2003, 2009).

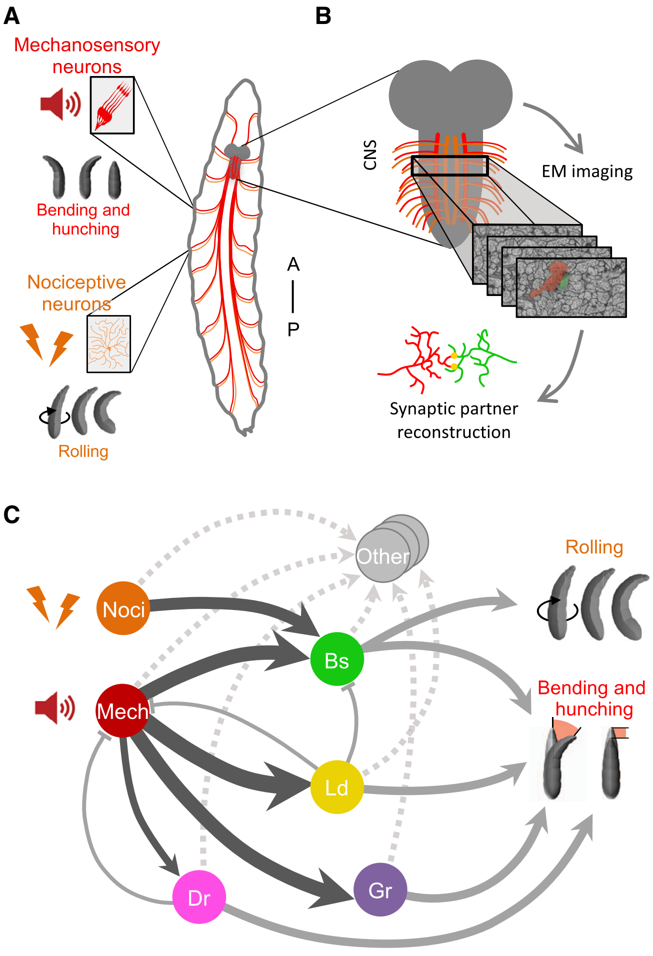

Figure 1. A Mechanosensory Circuit in *Drosophila* Larva Revealed by Electron Microscopy Reconstruction

(A) Schematic of the mechanosensory (Me) Chordotonal neurons and the nociceptive multidendritic class IV neurons, projecting their axons from the periphery to the VNC. Insets illustrate their morphology in the body wall. Vibration activates the Me neurons and elicits bending and hunching. Noxious stimulus activates the nociceptive neurons and elicits a rolling escape response.

(B) EM imaging and reconstruction reveal fine morphology and synaptic connectivity.

(C) Synaptic connectivity diagram of preferred local interneuron partners of the Me neurons (Jovanic et al., 2016; Ohyama et al., 2015). Strong activation of the excitatory multisensory Basin (Bs) triggers rolling; weak activation triggers bending and hunching. Inhibitory Drunken (Dr), Griddle (Gr), and Ladder (Ld) interneurons trigger bending and hunching though disinhibition. "Other," less strongly connected (dashed arrows) interneurons. Circles, neuron type. Dark arrows, connections that are analyzed in this study. Thickness of solid arrows, connectivity strength. Only connections greater than 1% of postsynaptic input in each hemisegment are shown.

Axonal and dendritic filopodia covered a cumulative exploratory area (mediolateral and anteroposterior axes) during development larger than the final area occupied by their mature arbors (Figures 2C–2E). This means that many transient filopodia covered a space not represented in the final morphology (Niell et al., 2004). However, the relative exploration area of Basin dendrites is notably larger than that of mechanosensory axons (Figure 2E) or Basin axons. This suggests that dendritic exploration coverage might be broad in nature and not tightly constrained by positional or partner-derived cues. Postsynaptic dendrites might also play a more active role in the search for appropriate partners (Mumm et al., 2006). We therefore hypothesized that mechanosensory axon terminals might provide the instructive signal to stabilize exploratory filopodia from their postsynaptic partner dendrites.

Postsynaptic Dendrites Follow Their Displaced Presynaptic Partner Axons

In order to test whether mechanosensory neurons can provide sufficient instructive cues to their postsynaptic partners to form synapses irrespective of their location, we genetically displaced the mechanosensory axons and asked whether their connections with postsynaptic partners remained intact (Figures 3A–3K). We sought to shift the mechanosensory terminals to the lateral edge of the neuropil, outside their normal termination domain and that of their postsynaptic partners, but still within reach of postsynaptic exploratory filopodia. A more drastic displacement could potentially make the presynaptic axons physically inaccessible to their partners' dendrites. We induced the displacement by overexpressing the chimeric receptor FraRobo (Bashaw and Goodman, 1999) exclusively in the mechanosensory neurons (Figure 3A). FraRobo consists of the ectodomain of Frazzled and the intracellular domain of Roundabout (Robo). Frazzled binds to Netrin, a positional cue secreted by midline glia, and promotes attraction to it (Kolodziej et al., 1996; Mitchell et al., 1996). Robo binds to Slit, also secreted by midline glia, and triggers repulsion from it (Brose et al., 1999; Dickson and Gilestro, 2006; Kidd et al., 1998, 1999; Simpson et al., 2000). Therefore, FraRobo combines properties from both receptors, binding to Netrin (like Frazzled) and mediating repulsion (like Robo) rather than attraction. The expression of FraRobo increased the sensitivity of the mechanosensory axons to Netrin, shifting them laterally, away from the midline (Figure 3B).

To investigate the effect of such lateral shift on synaptic connectivity, we imaged with synaptic resolution, using EM, 1.5 abdominal segments (encompassing entire A1) of a first instar larva with FraRobo expression in the mechanosensory neurons (Figure 3C). We then reconstructed the mechanosensory neurons and their preferred downstream partners in this volume (Figures 3D–3H; Data S1; Tables S1 and S2). This confirmed that the mechanosensory axons expressing FraRobo were indeed shifted closer to the lateral boundary of the neuropil. Individual axons were affected with different magnitudes, causing some to shift more than others. We then analyzed the effect of this lateral shift on morphology and connectivity of the postsynaptic partners.

Previous reconstructions revealed that mechanosensory neurons reproducibly make numerically strong connections with homologous neurons on the left and right hemisegments of the same individual, across different segments of the same individual, and across different individuals (Jovanic et al., 2016; Ohyama et al., 2015). Similar to other areas of the nervous system, if a neuron connects to mechanosensory neurons with at least 1, 5, 10, or 15 synapses in one hemisegment, the

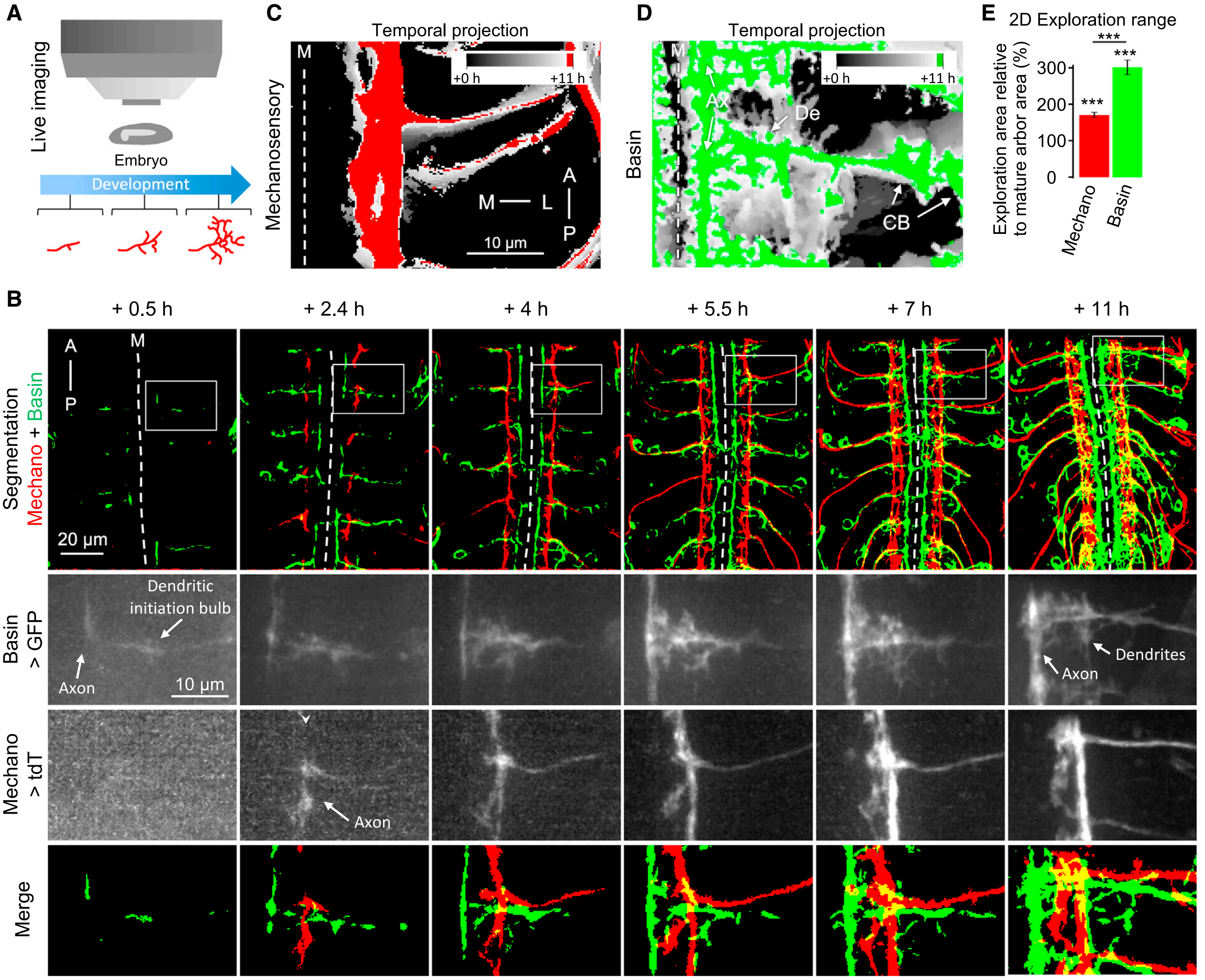

Figure 2. Postsynaptic Dendrites Have Significantly Broader Exploration Range Than Presynaptic Axons

(A) Me and Bs neurons were imaged in developing live embryos.

(B) Time lapse of Me (red) and Bs (green) neurons. Images are confocal Z-projections of a representative embryonic VNC. Time points are relative to the start of the imaging session (13 h AEL, time point + 0 h), when GFP in Bss is first detected. First row, entire field of view; subsequent rows, subregions marked with white square. Dashed line, midline (M).

(C and D) Temporal projection of Me axons (C) and Bs neurons (D) in one hemisegment.

(C) Cumulative Me exploration area (white) slightly exceeds the mature axon area (red).

(D) Bs dendritic filopodia explore most of their hemisegment, eventually adopting a final morphology (green) much more compact than the cumulative exploration area (white). Bs axons explore much less compared with their dendrites. CB, cell body; De, dendrites; Ax, axon.

(E) Bs dendrites and Me axons covered a wider cumulative area (mediolateral and anteroposterior axes) during developmental exploration than the area occupied by their respective mature arbor (stars above each bar; one-sample t test with default value of 100%). However, the relative exploration range of Bs dendrites is significantly larger than that of Me axons (Wilcoxon test). ***$p < 0.001$. n = 10 hemisegments each.

See also Video S1.

probability that the homologous neuron is connected (with ≥1 synapse) to mechanosensory neurons in the contralateral hemisegment of the same individual is 75%, 89%, 97%, and 100%, respectively. Thus, numerically weak connections are not conserved between the left and right sides of the same individual, but numerically strong connections are, even across individuals. We therefore focused our analysis on strongly connected (i.e., ≥15 synapses) neuron types with 100% chance of conserved connections from mechanosensory neurons, referring to these as "preferred partners." We excluded long-range intersegmental interneurons from our analysis because they exit the 1.5-segment EM volume, and their contained fragments cannot be unambiguously matched to a wild-type reference. We focused on preferred local partners, contained mostly within the EM volume and readily identifiable. Furthermore, in order to compare effects on excitatory and inhibitory neurons (Figure 1C),

CellPress
OPEN ACCESS

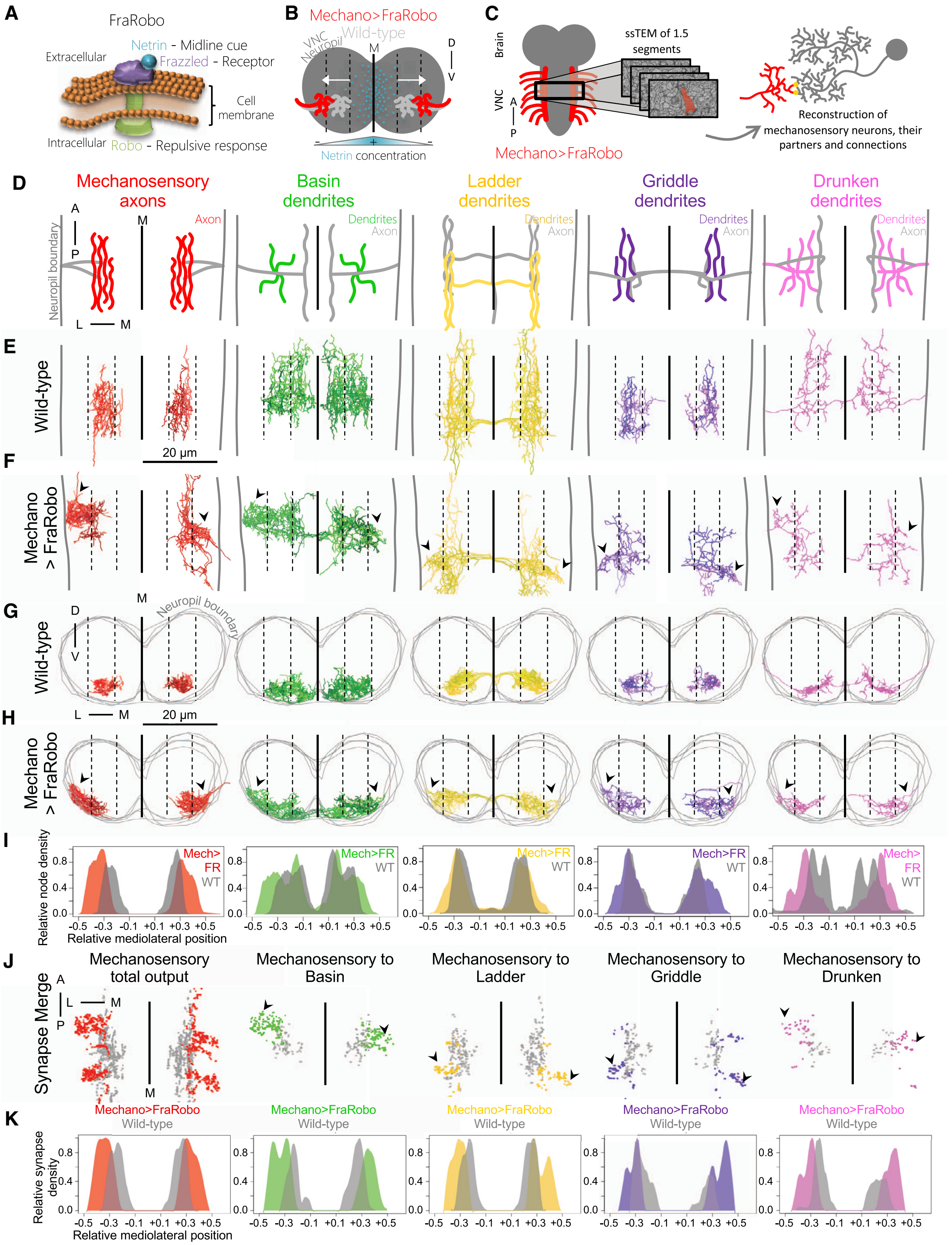

Figure 3. Postsynaptic Dendrites Extend Ectopic Branches to Reach Their Displaced Presynaptic Partner Axons

(A) FraRobo is a chimeric receptor with the ectodomain of Frazzled and the intracellular domain of Robo (Bashaw and Goodman, 1999). FraRobo binds to Netrin, triggering a repulsive response.

(B) Netrin concentration is highest at the M. Me axons expressing FraRobo (red) are more sensitive to Netrin and are repelled laterally compared with wild-type (WT) axons (light gray).

(legend continued on next page)

we chose neurons with available GAL4 lines and neurotransmitter profiles: three inhibitory interneurons types (Ladder, Griddle, and Drunken) and excitatory Basins that receive multisensory input from both mechanosensory and nociceptive neurons (Ohyama et al., 2015).

We first asked whether the preferred partners would extend ectopic lateral branches to follow the displaced mechanosensory axons. Indeed, the displacement of the mechanosensory axons caused a subsequent lateral shift of the dendrites of their postsynaptic partners (Figures 3E–3I, S1, and S2). Basins normally receive mechanosensory input in the medial and lateral subregions of their dendritic arbors and nociceptive input in the most medial portions. When the mechanosensory axons were shifted laterally, Basins broadened their dendritic coverage toward the lateral edge of the neuropil, a location they never occupy in wild-type animals. Ladder, Griddle, and Drunken also extended discrete ectopic lateral dendrites. Furthermore, these ectopic dendritic arbors received direct connections from the shifted mechanosensory axons (Figures 3J and 3K). As a control, we confirmed that the dendrites of Handle A, a neuron that normally does not receive synaptic input from mechanosensory axons (Jovanic et al., 2016), did not follow the shifted mechanosensory axons (Figure S3).

We reproduced an analogous postsynaptic displacement as a consequence of a presynaptic shift in a different pair of partners in the VNC: presynaptic dbd and postsynaptic A08a. Expression of Robo-2 or Unc-5 in dbd neurons causes an intermediate or strong lateral shift of their axons, respectively (Sales et al., 2019). We also observed a subsequent lateral shift in the dendritic distribution of A08a (Figure S4).

The most parsimonious explanation for the striking morphological adaptation of the postsynaptic interneuron dendrites in response to the displacement of their presynaptic axons is that these interneurons use partner-derived cues to recognize and follow their presynaptic partners even when they are in ectopic locations.

Presynaptic Axons Follow Their Displaced Postsynaptic Partner Axons

In addition to making synapses, the mechanosensory neurons also receive axo-axonic synapses from some inhibitory interneurons (Jovanic et al., 2016). To investigate the principles that govern the establishment of axo-axonic synapses, we analyzed the effect of the displacement of mechanosensory axons on their presynaptic partners' axons (Figures 4A–4I).

We found that the axons of Ladder and Drunken, which normally synapse onto mechanosensory axons, made clear ectopic branches that connected with the shifted mechanosensory axons (Figures 4B–4H). A portion of Ladder axons in one hemisegment was not shifted (Figure 4D). Interestingly, this corresponded to the location where mechanosensory axons were least shifted (Figure 3F). Although this variability was expected, it served as an internal (same sample) control, showing that the ectopic axons of Ladder interneurons are tightly correlated with the lateral displacement of their main synaptic partner, the mechanosensory neurons.

In contrast, the axons of Basin and Griddle interneurons were not displaced (Figures 4D, 4F, and 4G), likely because they do not normally form axo-axonic synapses onto mechanosensory neurons (Figures 4B and 4I), ruling out an overall shift in the entire neuropil resulting from the displacement of mechanosensory neurons. Thus, our results indicate that during the formation of axo-axonic connections, interneuron axons can follow their synaptic partners to ectopic locations.

Shifted Mechanosensory Axons Retain Most Preferred Partners and Do Not Gain New Ones

As shifting mechanosensory axons caused a subsequent shift of their synaptic partners, we next asked whether the connectivity between them was preserved (Figure 5A). For this, we analyzed all local postsynaptic partners (including the non-preferred ones; Data S2).

We found a lower number of total postsynaptic partners in the mechano > FraRobo EM volume than in wild-type. Interestingly, this reduction was at the expense of partners that receive very few mechanosensory inputs in wild-type. Twenty-four percent of neurons (9 of 37) receiving <15 mechanosensory synapses in wild-type failed to receive input from laterally displaced mechanosensory axons, while only 4% of neurons (1 of 23) receiving $\geq$15 mechanosensory synapses failed to do so (Figures 5B and 5C). Thus, the laterally shifted mechanosensory axons still connected to most of their preferred partners, presumably because the neurites of these partners followed them.

A purely location-based mechanism for synaptic specificity would predict that displaced mechanosensory axons will synapse onto new partners at the new location. Contrary to this, we found only one neuron downstream of the shifted mechanosensory axons that is not normally a partner in wild-type (Figure 5C). This new partner was numerically the most weakly connected, barely above the significant connectivity threshold of three synapses (Gerhard et al., 2017; Ohyama et al., 2015). This virtual absence of new partners and the retention of preferred partners show remarkable partner specificity despite the altered location of the mechanosensory neurons.

(C) EM reconstruction of shifted Me axons, all their synapses and preferred local partners.
(D) Schematic dorsal view of the Me axons and their preferred postsynaptic local partners in one abdominal segment. Colored regions are displayed in subsequent panels.
(E–H) Dorsal (E and F) and cross section (G and H) views of the reconstructed Me axons and postsynaptic dendrites in WT (E and G) and in a sample with FraRobo-expressing Me neurons (F and H). In mechano > FraRobo (Mech > FR), the Me axons are displaced laterally (arrowheads), away from the M (solid line), reaching the edge of the neuropil. The postsynaptic partners display ectopic branches in lateral domains (arrowheads). Neuropil boundary: gray vertical lines (D–F) or gray consecutive rings (G and H). Dashed lines split the width of the neuropil evenly in six sections.
(I) Node density distribution of reconstructed neurons (E–H) in the mediolateral axis in WT and Mech > FR.
(J) Overlay of reconstructed Me presynaptic sites in Mech > FR (colored) and WT (gray). Shifted Me axons make synapses in ectopic locations (arrowheads).
(K) Density distribution of Me synapses (shown in J).
See also Data S1 and Figures S1–S4.

CellPress
OPEN ACCESS

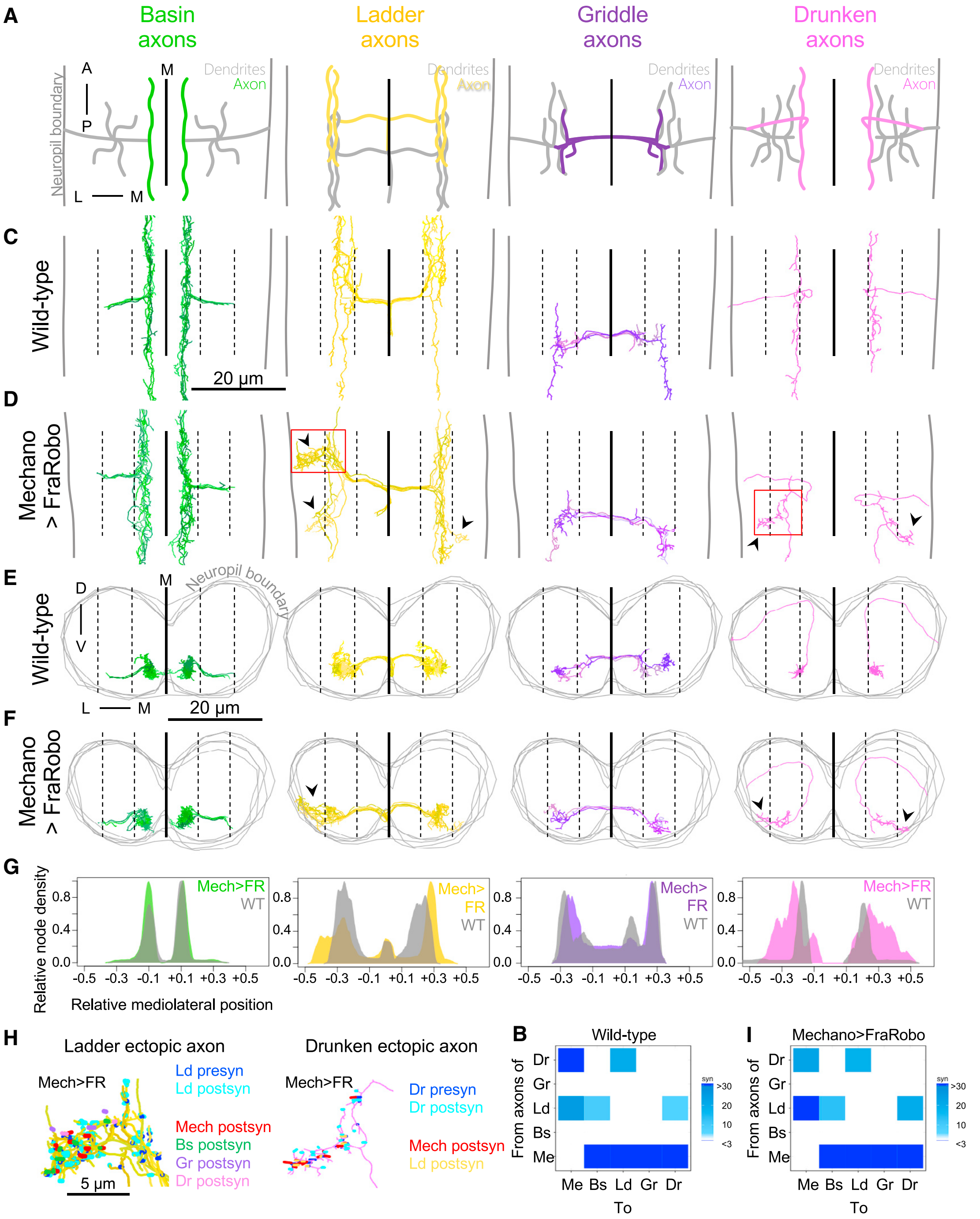

Figure 4. Presynaptic Axons Extend Ectopic Branches to Reach Their Displaced Postsynaptic Partner Axons

(A) Schematic dorsal view of Me axons and their preferred local partners in one abdominal segment. Colored regions are displayed in subsequent panels.

(B) Connectivity matrix of axon-to-whole-neuron connections between Me, Bs, Ld, Gr, and Dr neurons in WT (Jovanic et al., 2016). Connections with three or more synapses are shown.

(C–F) Dorsal (C and D) and cross section (E and F)) views of reconstructed axons of Me partners in WT (C and E) and Mech > FR (D and F). The axons of Ld and Dr, which normally synapse onto Me axons, extend ectopic branches in the Mech > FR sample (arrowheads). The axons of Bs and Gr interneurons, which normally do not synapse onto Me axons, do not extend ectopic branches. Image annotations as in Figures 3E–3H.

(G) Node density distribution of reconstructed axons (C–F) in WT and Mech > FR.

(legend continued on next page)

Interestingly, even though most postsynaptic partners connected with the laterally displaced mechanosensory axons, the numbers of mechanosensory synapses onto specific partners were altered. For example, Basin interneurons became the top partners of displaced mechanosensory neurons (Figure 5C), ranking higher than Ladder and Griddle interneurons, which are the top partners in wild-type (Figure 5B). This suggests that although the physical displacement of the presynaptic partners did not affect connectivity qualitatively, it may have a significant quantitative effect.

Shifting the Location of Mechanosensory Axons Alters Numbers of Connections with Partners, Generating Deficient Mechanosensory Behavior

The number of synapses in the nervous system increases throughout larval development (Gerhard et al., 2017), making it difficult to compare absolute synapse numbers across individuals. Therefore, to investigate in more detail the quantitative impact of the lateral shift of mechanosensory axons on connectivity, we computed the fraction of input their partners receive from them and the synapse density per unit of cable length (Figures 5D–5H and S5; Tables S1 and S2; see STAR Methods). We found that both the fractions of mechanosensory input and mechanosensory synapse density onto the excitatory Basin interneurons were significantly higher than in controls (Figures 5E and S5). Consistent with the connectivity increase, we found that optogenetic activation of mechanosensory neurons that express FraRobo evoked significantly stronger calcium responses in Basins compared with controls (Figures 5I and 5I′). In contrast, the fractions of mechanosensory input and the mechanosensory synapse density onto the inhibitory Griddle and Ladder were significantly lower than in wild-type (Figures 5G, 5H, and S5). Altogether, we found significant quantitative differences in connectivity from laterally shifted mechanosensory neurons onto their preferred partners.

We wondered whether these differences in connectivity lead to defects in the overall functional output of the circuit. Mechanosensory neurons are activated by sound-generated vibration, which elicits stereotypic body bending and hunching (Jovanic et al., 2016; Ohyama et al., 2013, 2015; Wu et al., 2011; Zhang et al., 2013). We found that larvae with mechanosensory neurons expressing FraRobo were still responsive to vibration. However, their bend duration and hunch probability and duration were significantly lower than in controls (Figures 5J and 5J′). Previous studies have shown that disinhibition plays a major role in triggering larval responses to mechanosensory stimuli (Jovanic et al., 2016). Therefore, these impaired behavioral responses could potentially be explained by the reduction of input from shifted mechanosensory neurons onto some inhibitory partners (Figures 5G, 5H, and S5). Therefore, despite evident partner specificity, precise positioning of synaptic partners in correct locations is important for the establishment of appropriate numbers of connections and appropriate function.

Silencing Mechanosensory Neurons during Development Alters the Numbers of Connections They Form with Specific Partners

Basin dendric filopodia first contact the mechanosensory axons during late embryonic development (Figure 2B), right before the onset of the first action potentials in the developing nervous system (Baines and Bate, 1998). This raises the possibility that neuronal activity might contribute to wiring specificity (Akin et al., 2019).

To investigate the role of neural activity in circuit assembly, we permanently blocked synaptic transmission in the mechanosensory neurons using tetanus toxin light chain (TNT) (Sweeney et al., 1995) and imaged 1.5 abdominal segments of a first instar larva using EM (Figure 6A). We then reconstructed the silenced mechanosensory neurons and their preferred downstream partners (Data S3; Tables S1 and S2). We found that the fraction of mechanosensory input onto the excitatory Basin interneurons was higher than in controls (Figure 6B). Interestingly, whereas previous studies have reported an increase in dendritic size following inactivation of a neuron or its excitatory presynaptic partners (Singh et al., 2010; Tripodi et al., 2008; Yuan et al., 2011), we found a significant increase in mechanosensory synapses onto Basin interneurons per millimeter cable (Figure S5) but no increase in Basin dendritic cable length compared with controls (Figure S6). In contrast, the fraction of mechanosensory input and the density of mechanosensory synapses onto the inhibitory Griddle and Ladder were lower than in controls (Figures 6C–6E and S5). Thus, silenced mechanosensory neurons could connect to their preferred partners, but the number of synapses with specific partners were altered, with opposite effects on excitatory and inhibitory interneurons (Figures 6B–6E).

Silencing Mechanosensory Neurons during Development Alters Functional Connections and Causes Permanent Defects in Mechanosensory Responses

We wondered whether the observed quantitative differences in connectivity induced by silencing mechanosensory neurons during embryonic development would be accompanied by differences in functional connectivity. We temporarily blocked synaptic transmission in mechanosensory neurons during embryonic development using temperature-sensitive Shibire (Shi^{ts1}) (Kitamoto, 2001) and later restored activity to test functional connectivity with postsynaptic partners in larvae (Figure 6F). Optogenetic activation of mechanosensory neurons that had been silenced during development evoked significantly larger calcium responses in Basins compared with controls (Figures 6G and 6G′). The increase in the strength of the functional connection

(H) Ectopic axonal branches of Ld and Dr (red squares in D) make appropriate synapses onto the shifted Me axons, and the shifted dendrites of other Me partner neurons. Abbreviations as in (B).
(I) Connectivity matrix of axon-to-whole-neuron connections between Me neurons and their partners (see B) in the Mech > FR volume. Note that the WT connectivity (B) is qualitatively preserved despite the shift of Me axons.
See also Data S1 and Figure S2.

CellPress
OPEN ACCESS

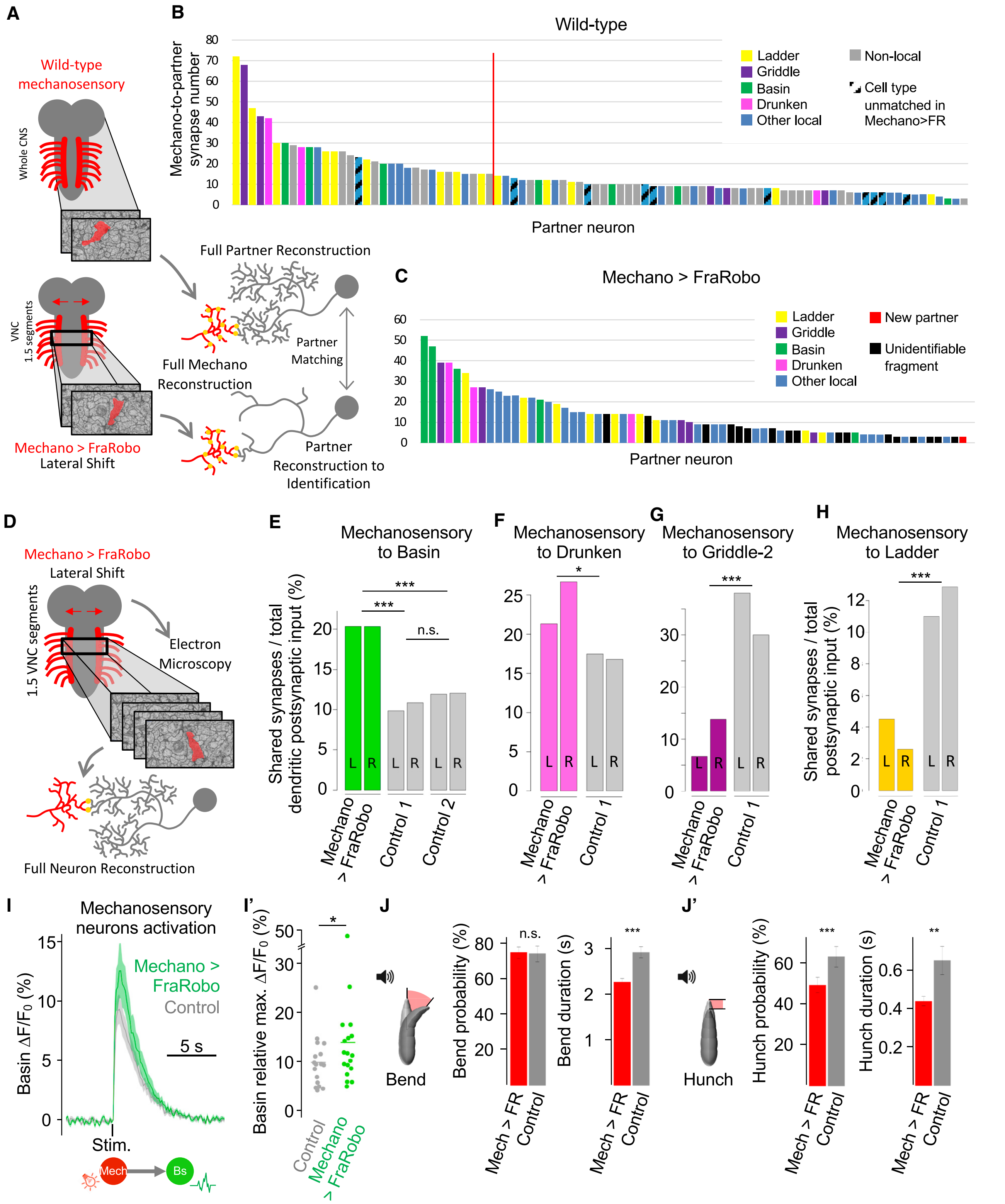

Figure 5. Shifting the Location of Sensory Axons Alters Numbers of Connections between Specific Partners, Generating Deficient Me Behavior

(A) Schematic of the comparison of Me partners in the Mech > FR and the WT EM volume. For Mech > FR, we reconstructed the principal arbors of all Me partners sufficiently for identification and matching to previously fully reconstructed neurons in WT.

(legend continued on next page)

is consistent with the increased number and density of mechanosensory synapses onto Basins, when mechanosensory neurons are silenced during development (Figure 6B).

We then investigated whether these differences in structural and functional connectivity affect the behavioral output of the circuit (Figure 6H). We found that early-stage larvae had a reduced probability of response to vibration compared with controls (Figures 6I and 6I′). This behavioral defect persisted in late-stage larvae even 5 days after activity was restored (Figures 6J and 6J′). These impaired behavioral responses could potentially be explained by the reduction in mechanosensory input onto inhibitory interneurons (Figures 6D and 6E). In summary, silencing mechanosensory neurons during development affected the numbers of synapses between specific partners and resulted in behavioral defects that persist days after activity restoration.

Silencing Mechanosensory Neurons Increases Nociceptive Input onto Basins and Increases Responsiveness to Nociceptive Stimulation

Basin interneurons process multisensory information from mechanosensory and nociceptive neurons (Ohyama et al., 2015). We therefore wondered whether silencing one sensory modality during embryonic development would affect inputs from the remaining functional modality. We found that Basin interneurons compensate for the lack of functional mechanosensory input by increasing structural input from nociceptive neurons (Figure 7A), accompanied by stronger functional connections between nociceptive and Basin neurons (Figures 7B and B′).

The increase in the fraction of mechanosensory and nociceptive synapses onto Basins (Figures 6B and 7A) must be at the expense of input from other neurons. Ladders provide feedforward inhibition from mechanosensory neurons onto Basins (Figure 1C). We found a significant reduction in Ladder inputs onto Basins in animals with silenced mechanosensory neurons (Figure S7), suggesting that the increase in the fraction of excitatory input onto Basins could be, at least in part, at the expense of inhibitory input.

In wild-type larvae, strong activation of multisensory Basin interneurons via nociceptive neurons, or via a combination of mechanosensory and nociceptive neurons, evokes a rolling escape response (Ohyama et al., 2015). We therefore asked whether the increase in structural and functional connections between nociceptive and Basin neurons observed after silencing mechanosensory neurons during development (Figures 7A–7B′) affects the behavioral responses to nociceptive stimuli (Figure 7C). To exclude possible effector-specific side effects (Nichols and Smith, 2019), we used several alternative approaches to manipulate activity. In all of these experiments, we observed an increase in rolling responses to the activation of nociceptive neurons (Figures 7D–7E′). As a control, silencing mechanosensory neurons only shortly before and during nociceptive activation (as opposed to silencing them throughout development) generated no significant differences in rolling responses (Figure 7F). These experiments show that silencing mechanosensory neurons during development results in increased structural and functional connections from nociceptive neurons onto Basins, along with increased responsiveness to nociceptive stimulation (Figure 7G).

DISCUSSION

Partners Find Each Other and Form Structural and Functional Connections Even in Aberrant Locations

In some systems the position of pre- or postsynaptic terminals is specified by non-partner-derived positional cues (Couton et al., 2015; Mauss et al., 2009; Sürmeli et al., 2011; Zlatic et al., 2003, 2009). In other systems, molecules have been identified that mediate partner matching (Ashrafi et al., 2014; Betley et al., 2009; Hong and Luo, 2014; Hong et al., 2012; Krishnaswamy et al., 2015; Pecho-Vrieseling et al., 2009; Sanes and Yamagata, 2009; Ward et al., 2015; Xu et al., 2018). However, it was unclear whether both mechanisms could operate in the same system and whether either mechanism specifies numbers of connections between partners.

Although developing sensory axons use non-partner-derived positional cues to select their final termination area in the

(B and C) Connectivity ranking plots of postsynaptic partners of Me neurons in one hemisegment in WT (B) and Mech > FR (C). Bars represent individual neurons. Synapse numbers are the sum of inputs from all eight Me axons onto single neurons. Only neurons with ≥3 synapses from any Me neuron in each hemisegment (left and right) in WT are shown. Local neurons mostly span 1–2 VNC segments and are identifiable in a 1.5-segment EM volume. Non-local neurons span multiple segments and can only be identified in bigger EM volumes. We only attempted to match local neurons from the Mech > FR volume (1.5 segments) to those in the WT volume.

(B) Local neurons not found downstream of Me neurons in FraRobo volume (C) are marked as unmatched. Red line separates partners strongly (≥15 synapses) and weakly (<15 synapses) connected to Me neurons.

(C) Neurons that receive synaptic input (reproducible in left and right hemisegments) from Me axons in Mech > FR, but not in WT are marked in red. Shifted Me neurons acquired only one new weakly connected partner in their ectopic location.

(D) Full reconstruction of Me neurons and their preferred local partners (Bs, Ld, Gr, and Dr) in the Mech > FR volume allowed the computation of synaptic input fractions and their comparison with WT.

(E–H) Fraction of synaptic input that preferred local partners receive from Me neurons. Connectivity from right (R) and left (L) sides of same segment shown separately to depict consistency within sample. Bs connectivity was also compared with a second control volume (control 2), in which these connections were previously reconstructed. *$p < 0.05$ and ***$p < 0.001$, chi-square test.

(I) Calcium responses (mean ± SEM) of Bs interneurons to the optogenetic activation of Me neurons.

(I′) Quantification of peak responses in (I). Experimental, n = 18 animals; control, n = 17. *$p < 0.05$, single-sided Wilcoxon rank-sum test.

(J and J′) Bend (J) and hunch (J′) responses to vibration in animals with shifted Me axons (mech > FR) and controls. Error bars represent the 95% confidence interval for probabilities or standard error for durations. For durations: experimental, n = 506 animals; control, n = 272. For probabilities: experimental, n = 677; control, n = 367. Probabilities compared using chi-square test for proportions; durations compared using double-sided t test. **$p < 0.01$ and ***$p < 0.001$.

See also Tables S1 and S2, Data S1 and S2, and Figures S5–S7.

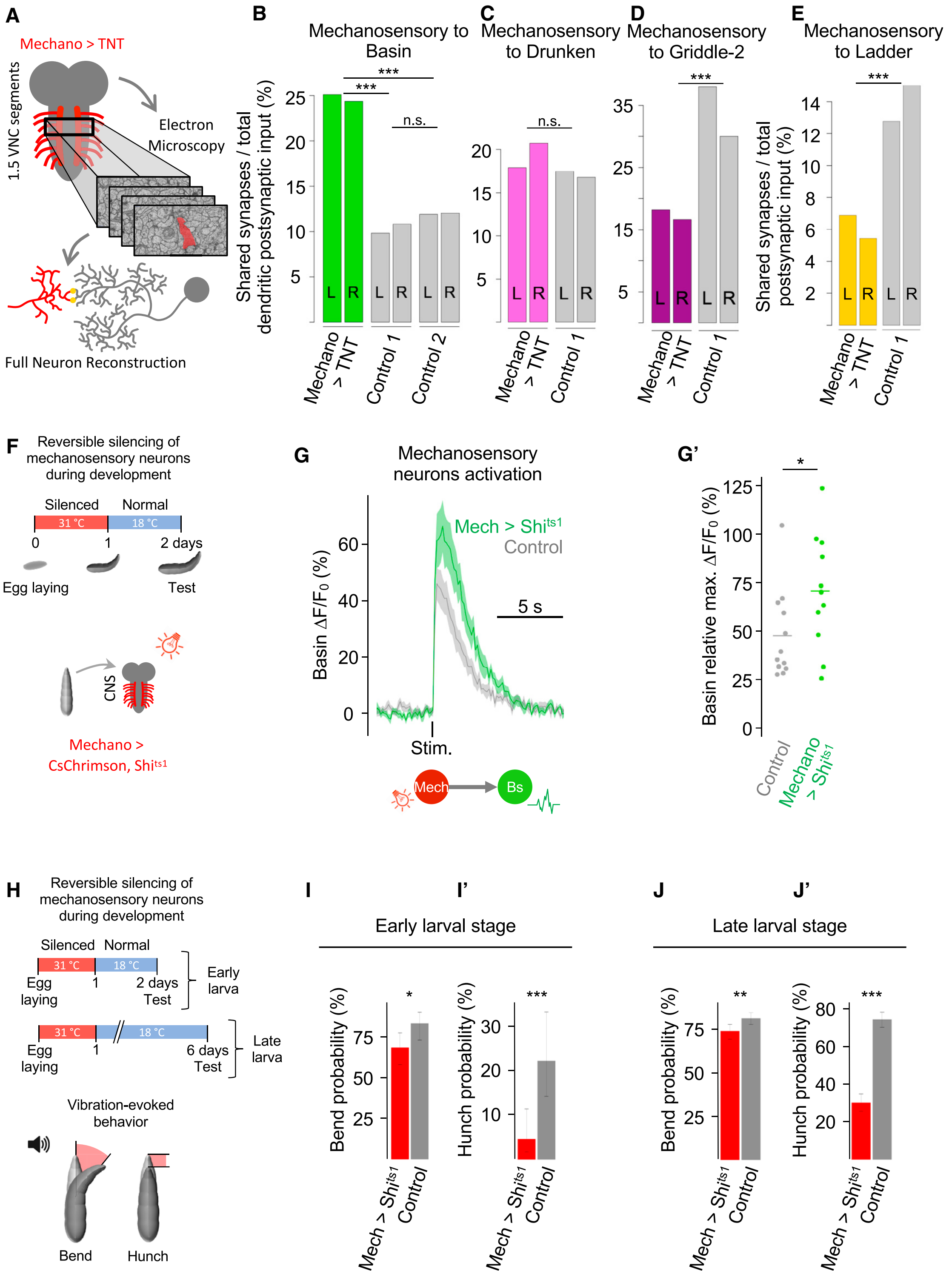

Figure 6. Silencing Me Neurons during Development Alters the Numbers of Connections between Specific Partners and Generates Defective Me Responses

(A) Full EM reconstruction of silenced Me neurons (mechano > TNT), all their synapses and preferred partners (Bs, Ld, Gr, and Dr).

(B–E) Fraction of synaptic input from silenced Me neurons onto preferred local partners. ***$p < 0.001$, chi-square test.

(F) Schematic of experimental conditions for reversible silencing of Me neurons during development used in (G) and (G′).

(G) Calcium responses (mean ± SEM) of Bs interneurons to the optogenetic activation of Me neurons that had been reversibly silenced during development (F).

(legend continued on next page)

Drosophila nerve cord (Zlatic et al., 2003, 2009), our results suggest that position alone does not specify connectivity and that partner recognition also exists. When we altered the location of sensory axons, their postsynaptic partners extended ectopic branches and formed synaptic connections with them (Figures 3, 4, and 5). The shifted axons did not gain any new strongly connected partners at their ectopic location (Figure 5C), providing further evidence of remarkable partner selectivity. It is hard to imagine which cue, other than the mechanosensory axons themselves, instructed partner dendrites to form these ectopic branches and synapses. Nevertheless, the final proof of the existence of the partner-derived cues will be their identification in the future.

Correct Partner Location Is Required for Forming Appropriate Numbers of Connections

If partner-recognition molecules are sufficient for selective synaptogenesis irrespective of the location of partners, why is the precise location of sensory neuron axon terminals so tightly regulated by non-partner-derived positional cues? Despite partner neurons' connecting in ectopic locations, they did not establish appropriate numbers of synapses (Figures 5E–5H), resulting in defective responses to mechanosensory stimuli (Figures 5J and 5J′). This indicates that precise positioning of presynaptic mechanosensory axons is necessary for the formation of appropriate number of synapses.

We do not know why some partners received more synapses from shifted mechanosensory axons and others fewer than in wild-type. One possibility could be the involvement of third-party guidepost cells in synaptogenesis (Shen and Bargmann, 2003; Shen et al., 2004) which would not be present in the aberrant location. Another speculation is that some neurons are better than others at finding their misplaced partners. Yet another possibility could be that shifting mechanosensory neurons initially resulted in fewer or weaker synaptic connections. This could have triggered compensatory homeostatic changes in the balance of excitation and inhibition within the circuit by increasing mechanosensory connections onto excitatory interneurons and reducing those onto inhibitory interneurons (Maffei and Turrigiano, 2008). This latter possibility could explain why we observed similar connectivity effects when sensory neurons were shifted (Figures 5E, 5I, and 5I′) and when they were inactivated during development (see below; Figures 6B, 6G, and 6G′).

Finally, in addition to changes in synapse numbers, silencing or shifting presynaptic partners could have also induced changes in synaptic strength and electrical properties (e.g., through changes in ion channel composition) that could account for some of the observed effects in behavior and functional connectivity (O'Leary et al., 2013; Santin and Schulz, 2019; Temporal et al., 2014). Furthermore, changes in the shapes of arbors could potentially affect electrical signal propagation. Future patch-clamp recordings following the same experimental manipulations could reveal the extent to which this occurs.

Silencing Sensory Neurons Changes the Balance of Excitatory and Inhibitory Connections

Activity plays a major role in refining the patterns of neuronal connections during development (Kutsarova et al., 2017; Leighton and Lohmann, 2016; Tien and Kerschensteiner, 2018), especially in vertebrates. However, the effects induced within the network in response to selective silencing of specific neuron types are not fully understood.

The role activity plays in the development of the insect central nervous system is less clear. Some studies have shown that a lack of sensory activity during development does not affect neuron morphology or the capacity to form connections (Baines et al., 2001; Constance et al., 2018; Hiesinger et al., 2006; Jefferis et al., 2004; Scott et al., 2003). Other studies have reported neural circuits can adapt their morphology, connectivity, or behavior in response to changes in developmental activity (Fushiki et al., 2013; Giachello and Baines, 2015, 2017; Kaneko et al., 2017; Prieto-Godino et al., 2012; Sheng et al., 2018; Tripodi et al., 2008; Wolfram and Baines, 2013; Yuan et al., 2011). However, a comprehensive synaptic-resolution analysis of the effects of silencing a specific neuron type on the numbers of connections between partners was lacking.

Our EM reconstructions revealed that silenced mechanosensory neurons connected to the appropriate partners, but with inappropriate numbers of synapses (Figures 6B, 6D, and 6E). Interestingly, excitatory multisensory interneurons (Basin) received a higher fraction of input from silenced mechanosensory neurons than in controls, while inhibitory interneurons (Ladder and Griddle) received a lower fraction. Selective silencing of mechanosensory neurons also increased input from a different sensory modality (nociceptive) onto Basin interneurons (Figure 7A) and decreased their input from inhibitory interneurons (Figure S7). This overall effect is similar to observations in the cortex, where sensory deprivation induces network-level homeostasis that alters the balance of excitation and inhibition (Maffei et al., 2004, Mendelsohn et al., 2015). Synaptic scaling in the cortex is thought to be multiplicative, such that all excitatory connections onto an excitatory neuron are scaled equally when excitatory drive onto that neuron is reduced (Turrigiano and Nelson, 2004). In contrast, the inhibitory connections onto excitatory neurons are reduced. Although the majority of studies in the cortex focus on homeostatic plasticity of functional connections, we demonstrate a drastic plasticity in the number of synaptic connections between partners (Figures 6B–6E and 7A). This apparent homeostasis of synapse numbers may follow similar multiplicative rules, because we found that both mechanosensory and nociceptive inputs onto Basin interneurons were increased when mechanosensory neurons were silenced (Figures 6B and 7A).

(G′) Quantification of the calcium responses in (G). Experimental, n = 11 animals; control, n = 12. *p < 0.05, single-sided Wilcoxon rank-sum test.
(H) Schematic of reversible silencing of Me neurons during development for behavioral experiments (I–J′).
(I–J′) Bend and hunch behavioral responses to vibration in animals with Me neurons that were reversibly silenced during development (mech > Shi^{ts1}) and controls. Reduced responses persist from early (I and I′) to late (J and J′) larval stages. For (I) and (I′): experimental, n = 86 animals; control, n = 72. For (J) and (J′): experimental, n = 380 animals; control, n = 476. Error bars represent the 95% confidence interval. *p < 0.05, **p < 0.01, and ***p < 0.001, chi-square test.
See also Tables S1 and S2, Data S3, and Figures S5–S7.

CellPress
OPEN ACCESS

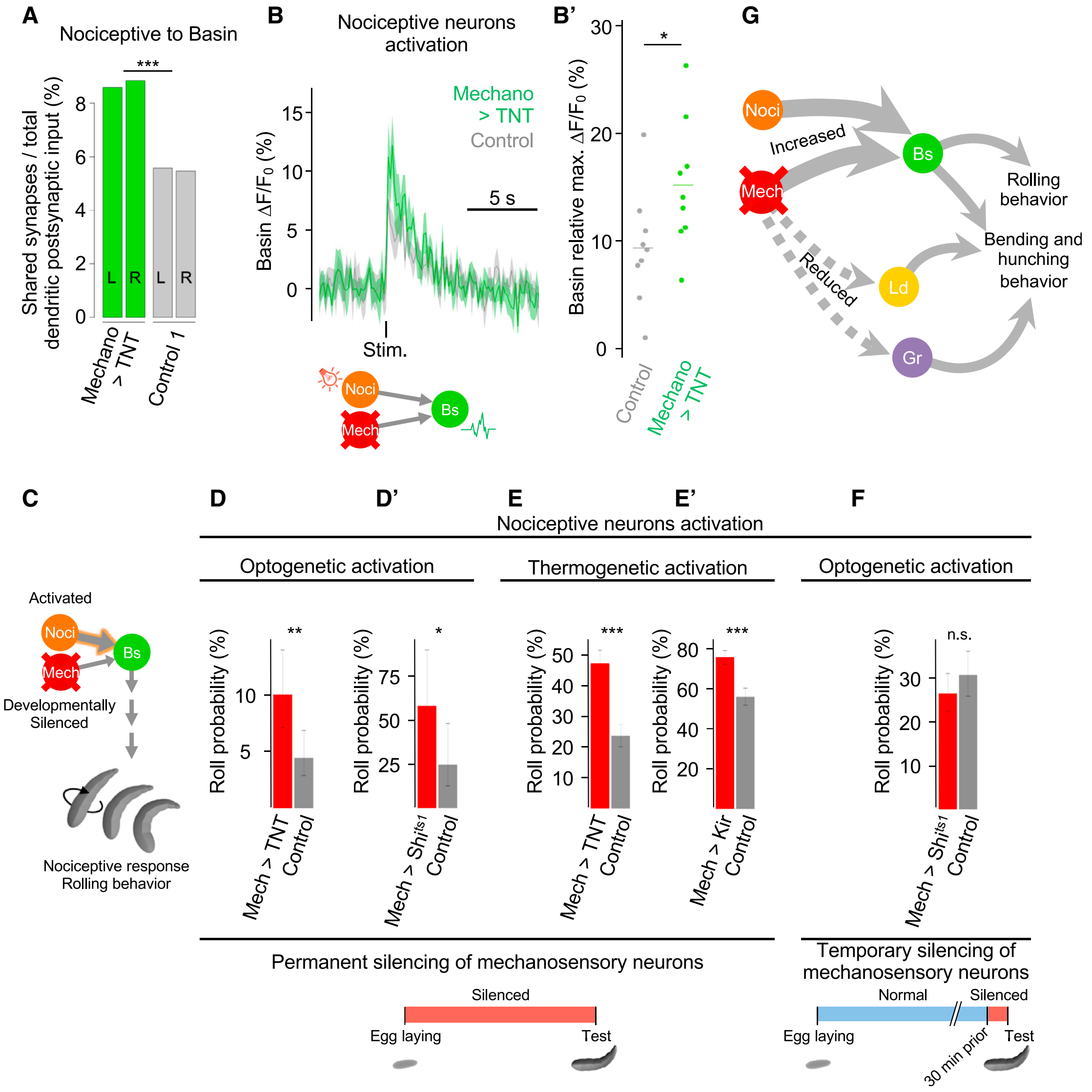

Figure 7. Bs Interneurons Compensate for Lack of Me Input by Increasing Nociceptive Input

(A) Fraction of input from nociceptive neurons onto Bs interneurons increases when Me neurons are silenced by the targeted expression of TNT, compared with control. ***p < 0.001, chi-square test.

(B) Calcium responses (mean ± SEM) of Bs interneurons to optogenetic activation of nociceptive neurons when Me neurons are silenced (mechano > TNT).

(B′) Quantification of calcium responses in (B). n = 9 animals for each condition. *p < 0.05, single-sided Wilcoxon rank-sum test.

(C) Schematic of behavioral experiments in which the Me neurons (mech) were temporarily or permanently silenced, and the nociceptive neurons (noci) were activated. Strong nociceptive activation can elicit rolling (Ohyama et al., 2015).

(D–F) Rolling probabilities upon activation (optogenetic in D, D′, and F; thermogenetic in E and E′) of nociceptive neurons and permanent (D–E′) or temporary (F) silencing of Me neurons (mech). Silencing achieved by targeted expression of TNT (D and E), Shi^{ts1} (D′ and F), or Kir (E′). Note that genotypes in (B) and (D) are the same. For (D): experimental, n = 298 animals; control, n = 426. For (D′): experimental, n = 310; control, n = 322. For (E), experimental, n = 550; control, n = 526. For (E′): experimental, n = 580; control, n = 512. For (F): experimental, n = 399; control, n = 305. Error bars represent 95% confidence interval. *p < 0.05, **p < 0.01, and ***p < 0.001, chi-square test.

(G) Summary of connectivity and behavioral effects of the developmental silencing of Me neurons. Abbreviations as in Figure 1C.

See also Tables S1 and S2, Data S3, and Figure S6.

Silencing Mechanosensory Neurons Enhances Nociceptive Responses and Permanently Reduces Mechanosensory Ones

We found that larvae with permanently silenced mechanosensory neurons not only had increased structural connections between nociceptive and Basin neurons (Figure 7A) but also stronger functional connections and behavioral responses to nociceptive stimuli (Figures 7B–7E′). This structural and behavioral compensation is reminiscent of findings in mammals, in which if one sensory modality is removed, another modality is

restructured and improved (Lomber et al., 2010; Rauschecker, 1995; Rauschecker and Korte, 1993).

Interestingly, silencing mechanosensory neurons during development permanently decreased responses to mechanosensory stimuli, even days after restoring activity (Figures 6I–6J′). This is also reminiscent of findings in mammals, in which deprivation of visual input during an early critical period permanently impairs vision (Hubel and Wiesel, 1964). However, this result appears at odds with the increased structural and functional connections from silenced mechanosensory neurons onto the excitatory Basins (Figures 6B, 6G, and 6G′). A possible explanation is the reduction of mechanosensory connections onto inhibitory neurons under the same conditions (Figures 6D and 6E). Unlike nociceptive neurons, the mechanosensory neurons have more inhibitory than excitatory postsynaptic partners, and these inhibitory interneurons play a role in triggering mechanosensory behaviors through disinhibition (Jovanic et al., 2016). Silencing the mechanosensory neurons may therefore result in a permanent reduction in disinhibition in the circuit with permanent consequences on behavior.

In summary, although partner-recognition molecules can ensure neurons recognize and connect only with appropriate partners, they are not sufficient to robustly specify appropriate numbers of synapses. Conversely, although neither precise location of presynaptic terminals nor neuronal activity in presynaptic partners directly instructs partner specificity, both are crucial to achieve appropriate numbers of connections, appropriate strengths of functional connections, appropriate balance of excitation and inhibition, and appropriate behavior. To our knowledge, our study reveals with unprecedented resolution how location, identity, and activity work together to give rise to appropriately wired neural circuits and appropriate behaviors.

STAR★METHODS

Detailed methods are provided in the online version of this paper and include the following:

- KEY RESOURCES TABLE
- RESOURCE AVAILABILITY
  - Lead Contact
  - Materials Availability
  - Data and Code Availability
- EXPERIMENTAL MODEL AND SUBJECT DETAILS
  - Fly stocks
- METHOD DETAILS
  - Live imaging
  - Calcium imaging with GCaMP
  - Behavioral assays
  - Electron microscopy volumes and reconstruction
  - Identification of manipulated neurons of interest in electron microscopy images
  - Immunohistochemistry
- QUANTIFICATION AND STATISTICAL ANALYSIS
  - Image processing for live imaging data
  - Image analysis of calcium imaging data
  - Electron microscopy connectivity analysis
  - Node and synapse distribution of reconstructed neurons
  - Statistical analysis
  - Quantification of mechanosensory, Basin and A08a membrane distribution

SUPPLEMENTAL INFORMATION

Supplemental Information can be found online at https://doi.org/10.1016/j.neuron.2020.10.004.

ACKNOWLEDGMENTS

We thank V. Jayaraman, G. Rubin, B. Dickson, A. Wong, and N. Brown for sharing fly lines; L. Narayan for technical assistance with behavior tracking; E. Trautman, R. Svirskas, and A. Stoychev for support with behavior analysis pipeline; K. Kilborn and the 3i team for guidance on holographic photostimulation; E. Croteau-Chonka for helping with behavioral experiments; C. Schneider-Mizell for assistance with EM reconstructions; and L. Zipursky for sharing equipment. We thank the Howard Hughes Medical Institute, the Wellcome Trust Investigator Awards 205050/B/16/Z and 205038/Z/16/Z, the European Research Council Consolidator Grant LeaRNN 819650, and the MRC MC_UP_1201/20 for funding.

AUTHOR CONTRIBUTIONS

J.V.-A. and M.Z. conceived the experiments and wrote the manuscript. J.V.-A. performed experiments and analyzed data. R.D.F. acquired the EM images. E.C.S. and E.L.H. performed immunohistochemistry experiments of A08a and dbd neurons. L.V. performed some behavioral experiments. A.C. registered the EM volumes. C.Q.D., M.L., and A.C. provided critical feedback on experimental design and writing of the manuscript.

DECLARATION OF INTERESTS

The authors declare no competing interests.

Received: December 18, 2019
Revised: August 12, 2020
Accepted: October 5, 2020
Published: October 28, 2020

REFERENCES

Abbott, L.F., and Nelson, S.B. (2000). Synaptic plasticity: taming the beast. Nat. Neurosci. *3* (*Suppl*), 1178–1183.

Ainsley, J.A., Pettus, J.M., Bosenko, D., Gerstein, C.E., Zinkevich, N., Anderson, M.G., Adams, C.M., Welsh, M.J., and Johnson, W.A. (2003). Enhanced locomotion caused by loss of the Drosophila DEG/ENaC protein Pickpocket1. Curr. Biol. *13*, 1557–1563.

Akin, O., Bajar, B.T., Keles, M.F., Frye, M.A., and Zipursky, S.L. (2019). Cell-type-specific patterned stimulus-independent neuronal activity in the Drosophila visual system during synapse formation. Neuron *101*, 894–904.e5.

Araújo, S.J., and Tear, G. (2003). Axon guidance mechanisms and molecules: lessons from invertebrates. Nat. Rev. Neurosci. *4*, 910–922.

Ashrafi, S., Betley, J.N., Comer, J.D., Brenner-Morton, S., Bar, V., Shimoda, Y., Watanabe, K., Peles, E., Jessell, T.M., and Kaltschmidt, J.A. (2014). Neuronal Ig/Caspr recognition promotes the formation of axoaxonic synapses in mouse spinal cord. Neuron *81*, 120–129.

Baines, R.A., and Bate, M. (1998). Electrophysiological development of central neurons in the Drosophila embryo. J. Neurosci. *18*, 4673–4683.

Baines, R.A., Uhler, J.P., Thompson, A., Sweeney, S.T., and Bate, M. (2001). Altered electrical properties in Drosophila neurons developing without synaptic transmission. J. Neurosci. *21*, 1523–1531.

CellPress
OPEN ACCESS

Bartol, T.M., Bromer, C., Kinney, J., Chirillo, M.A., Bourne, J.N., Harris, K.M., and Sejnowski, T.J. (2015). Nanoconnectomic upper bound on the variability of synaptic plasticity. eLife *4*, e10778.

Bashaw, G.J., and Goodman, C.S. (1999). Chimeric axon guidance receptors: the cytoplasmic domains of slit and netrin receptors specify attraction versus repulsion. Cell *97*, 917–926.

Betley, J.N., Wright, C.V.E.E., Kawaguchi, Y., Erdélyi, F., Szabó, G., Jessell, T.M., and Kaltschmidt, J.A. (2009). Stringent specificity in the construction of a GABAergic presynaptic inhibitory circuit. Cell *139*, 161–174.

Boulanger, J., Kervrann, C., Bouthemy, P., Elbau, P., Sibarita, J.-B., and Salamero, J. (2010). Patch-based nonlocal functional for denoising fluorescence microscopy image sequences. IEEE Trans. Med. Imaging *29*, 442–454.

Brose, K., Bland, K.S., Wang, K.H., Arnott, D., Henzel, W., Goodman, C.S., Tessier-Lavigne, M., and Kidd, T. (1999). Slit proteins bind Robo receptors and have an evolutionarily conserved role in repulsive axon guidance. Cell *96*, 795–806.

Brown, T.H., Zhao, Y., and Leung, V. (2009). Hebbian plasticity. In Encyclopedia of Neuroscience, L.R. Squire, ed. (Elsevier), pp. 1049–1056.

Burrone, J., and Murthy, V.N. (2003). Synaptic gain control and homeostasis. Curr. Opin. Neurobiol. *13*, 560–567.

Chen, M.S., Obar, R.A., Schroeder, C.C., Austin, T.W., Poodry, C.A., Wadsworth, S.C., and Vallee, R.B. (1991). Multiple forms of dynamin are encoded by shibire, a Drosophila gene involved in endocytosis. Nature *351*, 583–586.

Chen, T.-W., Wardill, T.J., Sun, Y., Pulver, S.R., Renninger, S.L., Baohan, A., Schreiter, E.R., Kerr, R.A., Orger, M.B., Jayaraman, V., et al. (2013). Ultrasensitive fluorescent proteins for imaging neuronal activity. Nature *499*, 295–300.

Constance, W.D., Mukherjee, A., Fisher, Y.E., Pop, S., Blanc, E., Toyama, Y., and Williams, D.W. (2018). Neurexin and Neuroligin-based adhesion complexes drive axonal arborisation growth independent of synaptic activity. eLife *7*, e31659.

Couton, L., Mauss, A.S., Yunusov, T., Diegelmann, S., Evers, J.F., and Landgraf, M. (2015). Development of connectivity in a motoneuronal network in Drosophila larvae. Curr. Biol. *25*, 568–576.

del Valle Rodríguez, A., Didiano, D., and Desplan, C. (2011). Power tools for gene expression and clonal analysis in Drosophila. Nat. Methods *9*, 47–55.

Denisov, G., Ohyama, T., Jovanic, T., and Zlatic, M. (2013). Model-based detection and analysis of animal behaviors using signals extracted by automated tracking. https://www.scitepress.org/Papers/2013/42351/42351.pdf.

Dickson, B.J. (2002). Molecular mechanisms of axon guidance. Science *298*, 1959–1964.

Dickson, B.J., and Gilestro, G.F. (2006). Regulation of commissural axon pathfinding by slit and its Robo receptors. Annu. Rev. Cell Dev. Biol. *22*, 651–675.

Eichler, K., Li, F., Litwin-Kumar, A., Park, Y., Andrade, I., Schneider-Mizell, C.M., Saumweber, T., Huser, A., Eschbach, C., Gerber, B., et al. (2017). The complete connectome of a learning and memory centre in an insect brain. Nature *548*, 175–182.

Fukuhara, K., Imai, F., Ladle, D.R., Katayama, K., Leslie, J.R., Arber, S., Jessell, T.M., and Yoshida, Y. (2013). Specificity of monosynaptic sensory-motor connections imposed by repellent Sema3E-PlexinD1 signaling. Cell Rep. *5*, 748–758.

Fushiki, A., Kohsaka, H., and Nose, A. (2013). Role of sensory experience in functional development of Drosophila motor circuits. PLoS ONE *8*, e62199.

Gerhard, S., Andrade, I., Fetter, R.D., Cardona, A., and Schneider-Mizell, C.M. (2017). Conserved neural circuit structure across *Drosophila* larval development revealed by comparative connectomics. eLife *6*, e29089.

Giachello, C.N., and Baines, R.A. (2015). Inappropriate neural activity during a sensitive period in embryogenesis results in persistent seizure-like behavior. Curr. Biol. *25*, 2964–2968.

Giachello, C.N., and Baines, R.A. (2017). Regulation of motoneuron excitability and the setting of homeostatic limits. Curr. Opin. Neurobiol. *43*, 1–6.

Gomez-Marin, A., Stephens, G.J., and Louis, M. (2011). Active sampling and decision making in Drosophila chemotaxis. Nat. Commun. *2*, 441.

Goodman, C.S. (1978). Isogenic grasshoppers: genetic variability in the morphology of identified neurons. J. Comp. Neurol. *182*, 681–705.

Hamada, F.N., Rosenzweig, M., Kang, K., Pulver, S.R., Ghezzi, A., Jegla, T.J., and Garrity, P.A. (2008). An internal thermal sensor controlling temperature preference in Drosophila. Nature *454*, 217–220.

Hamood, A.W., and Marder, E. (2015). Corrigendum: animal-to-animal variability in neuromodulation and circuit function. Cold Spring Harb. Symp. Quant. Biol. Published online May 6, 2015. https://doi.org/10.1101/sqb.2014.79.026955.

Helmstaedter, M., Briggman, K.L., and Denk, W. (2011). High-accuracy neurite reconstruction for high-throughput neuroanatomy. Nat. Neurosci. *14*, 1081–1088.

Helmstaedter, M., Briggman, K.L., Turaga, S.C., Jain, V., Seung, H.S., and Denk, W. (2013). Connectomic reconstruction of the inner plexiform layer in the mouse retina. Nature *500*, 168–174.

Hiesinger, P.R., Zhai, R.G., Zhou, Y., Koh, T.W., Mehta, S.Q., Schulze, K.L., Cao, Y., Verstreken, P., Clandinin, T.R., Fischbach, K.F., et al. (2006). Activity-independent prespecification of synaptic partners in the visual map of Drosophila. Curr. Biol. *16*, 1835–1843.

Hong, W., and Luo, L. (2014). Genetic control of wiring specificity in the fly olfactory system. Genetics *196*, 17–29.

Hong, W., Mosca, T.J., and Luo, L. (2012). Teneurins instruct synaptic partner matching in an olfactory map. Nature *484*, 201–207.

Hubel, D.H., and Wiesel, T.N. (1964). Effects of monocular deprivation in kittens. Naunyn Schmiedebergs Arch. Exp. Pathol. Pharmakol. *248*, 492–497.

Hwang, R.Y., Zhong, L., Xu, Y., Johnson, T., Zhang, F., Deisseroth, K., and Tracey, W.D. (2007). Nociceptive neurons protect Drosophila larvae from parasitoid wasps. Curr. Biol. *17*, 2105–2116.

Jefferis, G.S.X.E., Vyas, R.M., Berdnik, D., Ramaekers, A., Stocker, R.F., Tanaka, N.K., Ito, K., and Luo, L. (2004). Developmental origin of wiring specificity in the olfactory system of Drosophila. Development *131*, 117–130.

Jenett, A., Rubin, G.M., Ngo, T.-T.B., Shepherd, D., Murphy, C., Dionne, H., Pfeiffer, B.D., Cavallaro, A., Hall, D., Jeter, J., et al. (2012). A GAL4-driver line resource for Drosophila neurobiology. Cell Rep. *2*, 991–1001.

Johns, D.C., Marx, R., Mains, R.E., O'Rourke, B., and Marbán, E. (1999). Inducible genetic suppression of neuronal excitability. J. Neurosci. *19*, 1691–1697.

Jovanic, T., Schneider-Mizell, C.M., Shao, M., Masson, J.-B., Denisov, G., Fetter, R.D., Mensh, B.D., Truman, J.W., Cardona, A., and Zlatic, M. (2016). Competitive disinhibition mediates behavioral choice and sequences in Drosophila. Cell *167*, 858–870.e19.

Kane, E.A., Gershow, M., Afonso, B., Larderet, I., Klein, M., Carter, A.R., de Bivort, B.L., Sprecher, S.G., and Samuel, A.D.T. (2013). Sensorimotor structure of Drosophila larva phototaxis. Proc. Natl. Acad. Sci. U S A *110*, E3868–E3877.

Kaneko, T., Macara, A.M., Li, R., Hu, Y., Iwasaki, K., Dunnings, Z., Firestone, E., Horvatic, S., Guntur, A., Shafer, O.T., et al. (2017). Serotonergic modulation enables pathway-specific plasticity in a developing sensory circuit in Drosophila. Neuron *95*, 623–638.e4.

Kang, K., Panzano, V.C., Chang, E.C., Ni, L., Dainis, A.M., Jenkins, A.M., Regna, K., Muskavitch, M.A.T., and Garrity, P.A. (2011). Modulation of TRPA1 thermal sensitivity enables sensory discrimination in Drosophila. Nature *481*, 76–80.

Kidd, T., Russell, C., Goodman, C.S., and Tear, G. (1998). Dosage-sensitive and complementary functions of roundabout and commissureless control axon crossing of the CNS midline. Neuron *20*, 25–33.

Kidd, T., Bland, K.S., and Goodman, C.S. (1999). Slit is the midline repellent for the robo receptor in Drosophila. Cell *96*, 785–794.

Kilman, V., van Rossum, M.C.W., and Turrigiano, G.G. (2002). Activity deprivation reduces miniature IPSC amplitude by decreasing the number of

postsynaptic GABA(A) receptors clustered at neocortical synapses. J. Neurosci. *22*, 1328–1337.

Kitamoto, T. (2001). Conditional modification of behavior in Drosophila by targeted expression of a temperature-sensitive Shibire allele in defined neurons. J. Neurobiol. *47*, 81–92.

Klapoetke, N.C., Murata, Y., Kim, S.S., Pulver, S.R., Birdsey-Benson, A., Cho, Y.K., Morimoto, T.K., Chuong, A.S., Carpenter, E.J., Tian, Z., et al. (2014). Independent optical excitation of distinct neural populations. Nat. Methods *11*, 338–346.

Klein, M., Afonso, B., Vonner, A.J., Hernandez-Nunez, L., Berck, M., Tabone, C.J., Kane, E.A., Pieribone, V.A., Nitabach, M.N., Cardona, A., et al. (2015). Sensory determinants of behavioral dynamics in Drosophila thermotaxis. Proc. Natl. Acad. Sci. U S A *112*, E220–E229.

Kolodkin, A.L., and Tessier-Lavigne, M. (2011). Mechanisms and molecules of neuronal wiring: a primer. Cold Spring Harb. Perspect. Biol. *3*, 3.

Kolodziej, P.A., Timpe, L.C., Mitchell, K.J., Fried, S.R., Goodman, C.S., Jan, L.Y., and Jan, Y.N. (1996). frazzled encodes a Drosophila member of the DCC immunoglobulin subfamily and is required for CNS and motor axon guidance. Cell *87*, 197–204.

Krishnaswamy, A., Yamagata, M., Duan, X., Hong, Y.K., and Sanes, J.R. (2015). Sidekick 2 directs formation of a retinal circuit that detects differential motion. Nature *524*, 466–470.

Kutsarova, E., Munz, M., and Ruthazer, E.S. (2017). Rules for shaping neural connections in the developing brain. Front. Neural Circuits *10*, 111.

Kwon, Y., Shen, W.L., Shim, H.-S., and Montell, C. (2010). Fine thermotactic discrimination between the optimal and slightly cooler temperatures via a TRPV channel in Chordotonal neurons. J. Neurosci. *30*, 10465–10471.

Langley, J.N. (1895). Note on regeneration of prae-ganglionic fibres of the sympathetic. J. Physiol. *18*, 280–284.

Le, T., Liang, Z., Patel, H., Yu, M.H., Sivasubramaniam, G., Slovitt, M., Tanentzapf, G., Mohanty, N., Paul, S.M., Wu, V.M., et al. (2006). A new family of Drosophila balancer chromosomes with a w- dfd-GMR yellow fluorescent protein marker. Genetics *174*, 2255–2257.

Lee, W.-C.A., Bonin, V., Reed, M., Graham, B.J., Hood, G., Glattfelder, K., and Reid, R.C. (2016). Anatomy and function of an excitatory network in the visual cortex. Nature *532*, 370–374.

Leighton, A.H., and Lohmann, C. (2016). The wiring of developing sensory circuits-from patterned spontaneous activity to synaptic plasticity mechanisms. Front. Neural Circuits *10*, 71.

Lomber, S.G., Meredith, M.A., and Kral, A. (2010). Cross-modal plasticity in specific auditory cortices underlies visual compensations in the deaf. Nat. Neurosci. *13*, 1421–1427.

Louis, M., and de Polavieja, G. (2017). Collective behavior: social digging in Drosophila larvae. Curr. Biol. *27*, R1010–R1012.

Lu, J., Tapia, J.C., White, O.L., and Lichtman, J.W. (2009). The interscutularis muscle connectome. PLoS Biol. *7*, e32.

Luo, L., Gershow, M., Rosenzweig, M., Kang, K., Fang-Yen, C., Garrity, P.A., and Samuel, A.D.T. (2010). Navigational decision making in Drosophila thermotaxis. J. Neurosci. *30*, 4261–4272.

Maffei, A., and Turrigiano, G.G. (2008). Multiple modes of network homeostasis in visual cortical layer 2/3. J. Neurosci. *28*, 4377–4384.

Maffei, A., Nelson, S.B., and Turrigiano, G.G. (2004). Selective reconfiguration of layer 4 visual cortical circuitry by visual deprivation. Nat. Neurosci. *7*, 1353–1359.

Malenka, R.C., and Bear, M.F. (2004). LTP and LTD: an embarrassment of riches. Neuron *44*, 5–21.

Marder, E. (2011). Variability, compensation, and modulation in neurons and circuits. Proc. Natl. Acad. Sci. U S A *108* (*Suppl 3*), 15542–15548.

Marder, E., and Goaillard, J.-M. (2006). Variability, compensation and homeostasis in neuron and network function. Nat. Rev. Neurosci. *7*, 563–574.

Mauss, A., Tripodi, M., Evers, J.F., and Landgraf, M. (2009). Midline signalling systems direct the formation of a neural map by dendritic targeting in the Drosophila motor system. PLoS Biol. *7*, e1000200.

Mendelsohn, A.I., Simon, C.M., Abbott, L.F., Mentis, G.Z., and Jessell, T.M. (2015). Activity regulates the incidence of heteronymous sensory-motor connections. Neuron *87*, 111–123.

Mitchell, K.J., Doyle, J.L., Serafini, T., Kennedy, T.E., Tessier-Lavigne, M., Goodman, C.S., and Dickson, B.J. (1996). Genetic analysis of Netrin genes in Drosophila: Netrins guide CNS commissural axons and peripheral motor axons. Neuron *17*, 203–215.

Mogami, K., and Hotta, Y. (1981). Isolation of Drosophila flightless mutants which affect myofibrillar proteins of indirect flight muscle. Mol. Gen. Genet. *183*, 409–417.

Mumm, J.S., Williams, P.R., Godinho, L., Koerber, A., Pittman, A.J., Roeser, T., Chien, C.-B., Baier, H., and Wong, R.O.L. (2006). In vivo imaging reveals dendritic targeting of laminated afferents by zebrafish retinal ganglion cells. Neuron *52*, 609–621.

Nichols, E.L., and Smith, C.J. (2019). Synaptic-like vesicles facilitate pioneer axon invasion. Curr. Biol. *29*, 2652–2664.e4.

Niell, C.M., Meyer, M.P., and Smith, S.J. (2004). In vivo imaging of synapse formation on a growing dendritic arbor. Nat. Neurosci. *7*, 254–260.

O'Donnell, P.T., and Bernstein, S.I. (1988). Molecular and ultrastructural defects in a Drosophila myosin heavy chain mutant: differential effects on muscle function produced by similar thick filament abnormalities. J. Cell Biol. *107*, 2601–2612.

O'Leary, T., Williams, A.H., Caplan, J.S., and Marder, E. (2013). Correlations in ion channel expression emerge from homeostatic tuning rules. Proc. Natl. Acad. Sci. U S A *110*, E2645–E2654.

Ohyama, T., Jovanic, T., Denisov, G., Dang, T.C., Hoffmann, D., Kerr, R.A., and Zlatic, M. (2013). High-throughput analysis of stimulus-evoked behaviors in Drosophila larva reveals multiple modality-specific escape strategies. PLoS ONE *8*, e71706.

Ohyama, T., Schneider-Mizell, C.M., Fetter, R.D., Aleman, J.V., Franconville, R., Rivera-Alba, M., Mensh, B.D., Branson, K.M., Simpson, J.H., Truman, J.W., et al. (2015). A multilevel multimodal circuit enhances action selection in Drosophila. Nature *520*, 633–639.

Pecho-Vrieseling, E., Sigrist, M., Yoshida, Y., Jessell, T.M., and Arber, S. (2009). Specificity of sensory-motor connections encoded by Sema3e-Plxnd1 recognition. Nature *459*, 842–846.

Peters, A., and Feldman, M.L. (1976). The projection of the lateral geniculate nucleus to area 17 of the rat cerebral cortex. I. General description. J. Neurocytol. *5*, 63–84.

Pfeiffer, B.D., Jenett, A., Hammonds, A.S., Ngo, T.-T.B., Misra, S., Murphy, C., Scully, A., Carlson, J.W., Wan, K.H., Laverty, T.R., et al. (2008). Tools for neuroanatomy and neurogenetics in Drosophila. Proc. Natl. Acad. Sci. U S A *105*, 9715–9720.

Pfeiffer, B.D., Ngo, T.-T.B., Hibbard, K.L., Murphy, C., Jenett, A., Truman, J.W., and Rubin, G.M. (2010). Refinement of tools for targeted gene expression in Drosophila. Genetics *186*, 735–755.

Pfeiffer, B.D., Truman, J.W., and Rubin, G.M. (2012). Using translational enhancers to increase transgene expression in Drosophila. Proc. Natl. Acad. Sci. U S A *109*, 6626–6631.

Prieto-Godino, L.L., Diegelmann, S., and Bate, M. (2012). Embryonic origin of olfactory circuitry in Drosophila: contact and activity-mediated interactions pattern connectivity in the antennal lobe. PLoS Biol. *10*, e1001400.

R Core Team (2015). R: A Language and Environment for Statistical Computing. https://www.r-project.org.

Rauschecker, J.P. (1995). Compensatory plasticity and sensory substitution in the cerebral cortex. Trends Neurosci. *18*, 36–43.

Rauschecker, J.P., and Korte, M. (1993). Auditory compensation for early blindness in cat cerebral cortex. J. Neurosci. *13*, 4538–4548.

CellPress
OPEN ACCESS

Rees, C.L., Moradi, K., and Ascoli, G.A. (2017). Weighing the evidence in Peters' rule: does neuronal morphology predict connectivity? Trends Neurosci. *40*, 63–71.

Robertson, J.L., Tsubouchi, A., and Tracey, W.D. (2013). Larval defense against attack from parasitoid wasps requires nociceptive neurons. PLoS ONE *8*, e78704.

Rutherford, L.C., Nelson, S.B., and Turrigiano, G.G. (1998). BDNF has opposite effects on the quantal amplitude of pyramidal neuron and interneuron excitatory synapses. Neuron *21*, 521–530.

Ryan, K., Lu, Z., and Meinertzhagen, I.A. (2016). The CNS connectome of a tadpole larva of *Ciona intestinalis* (L.) highlights sidedness in the brain of a chordate sibling. eLife *5*, e16962.

Saalfeld, S., Cardona, A., Hartenstein, V., and Tomancak, P. (2009). CATMAID: collaborative annotation toolkit for massive amounts of image data. Bioinformatics *25*, 1984–1986.

Sales, E.C., Heckman, E.L., Warren, T.L., and Doe, C.Q. (2019). Regulation of subcellular dendritic synapse specificity by axon guidance cues. eLife *8*, e43478.

Sanes, J.R., and Yamagata, M. (2009). Many paths to synaptic specificity. Annu. Rev. Cell Dev. Biol. *25*, 161–195.

Santin, J.M., and Schulz, D.J. (2019). Membrane voltage is a direct feedback signal that influences correlated ion channel expression in neurons. Curr. Biol. *29*, 1683–1688.e2.

Schindelin, J., Arganda-Carreras, I., Frise, E., Kaynig, V., Longair, M., Pietzsch, T., Preibisch, S., Rueden, C., Saalfeld, S., Schmid, B., et al. (2012). Fiji: an open-source platform for biological-image analysis. Nat. Methods *9*, 676–682.

Schneider-Mizell, C.M., Gerhard, S., Longair, M., Kazimiers, T., Li, F., Zwart, M.F., Champion, A., Midgley, F.M., Fetter, R.D., Saalfeld, S., and Cardona, A. (2016). Quantitative neuroanatomy for connectomics in Drosophila. eLife *5*, e12059.

Schulz, D.J., and Lane, B.J. (2017). Homeostatic plasticity of excitability in crustacean central pattern generator networks. Curr. Opin. Neurobiol. *43*, 7–14.

Scott, E.K., Reuter, J.E., and Luo, L. (2003). Dendritic development of Drosophila high order visual system neurons is independent of sensory experience. BMC Neurosci. *4*, 14.

Shen, K., and Bargmann, C.I. (2003). The immunoglobulin superfamily protein SYG-1 determines the location of specific synapses in C. elegans. Cell *112*, 619–630.

Shen, K., Fetter, R.D., and Bargmann, C.I. (2004). Synaptic specificity is generated by the synaptic guidepost protein SYG-2 and its receptor, SYG-1. Cell *116*, 869–881.

Sheng, C., Javed, U., Gibbs, M., Long, C., Yin, J., Qin, B., and Yuan, Q. (2018). Experience-dependent structural plasticity targets dynamic filopodia in regulating dendrite maturation and synaptogenesis. Nat. Commun. *9*, 3362.

Simpson, J.H., Bland, K.S., Fetter, R.D., and Goodman, C.S. (2000). Short-range and long-range guidance by Slit and its Robo receptors: a combinatorial code of Robo receptors controls lateral position. Cell *103*, 1019–1032.

Singh, A.P., VijayRaghavan, K., and Rodrigues, V. (2010). Dendritic refinement of an identified neuron in the Drosophila CNS is regulated by neuronal activity and Wnt signaling. Development *137*, 1351–1360.

Sommer, C., Straehle, C., Koethe, U., and Hamprecht, F.A. (2011). ilastik: Interactive Learning and Segmentation Toolkit. In 8th IEEE International Symposium on Biomedical Imaging (ISBI 2011 (Institute of Electrical and Electronics Engineers).

Sperry, R.W. (1963). Chemoaffinity in the orderly growth of nerve fiber patterns and connections. Proc. Natl. Acad. Sci. U S A *50*, 703–710.

Sugie, A., Marchetti, G., and Tavosanis, G. (2018). Structural aspects of plasticity in the nervous system of Drosophila. Neural Dev. *13*, 14.

Sürmeli, G., Akay, T., Ippolito, G.C., Tucker, P.W., and Jessell, T.M. (2011). Patterns of spinal sensory-motor connectivity prescribed by a dorsoventral positional template. Cell *147*, 653–665.

Sweeney, S.T., Broadie, K., Keane, J., Niemann, H., and O'Kane, C.J. (1995). Targeted expression of tetanus toxin light chain in Drosophila specifically eliminates synaptic transmission and causes behavioral defects. Neuron *14*, 341–351.

Swierczek, N.A., Giles, A.C., Rankin, C.H., and Kerr, R.A. (2011). High-throughput behavioral analysis in C. elegans. Nat. Methods *8*, 592–598.

Takemura, S.Y., Bharioke, A., Lu, Z., Nern, A., Vitaladevuni, S., Rivlin, P.K., Katz, W.T., Olbris, D.J., Plaza, S.M., Winston, P., et al. (2013). A visual motion detection circuit suggested by Drosophila connectomics. Nature *500*, 175–181.

Takemura, S.Y., Xu, C.S., Lu, Z., Rivlin, P.K., Parag, T., Olbris, D.J., Plaza, S., Zhao, T., Katz, W.T., Umayam, L., et al. (2015). Synaptic circuits and their variations within different columns in the visual system of Drosophila. Proc. Natl. Acad. Sci. U S A *112*, 13711–13716.

Takemura, S.Y., Aso, Y., Hige, T., Wong, A., Lu, Z., Xu, C.S., Rivlin, P.K., Hess, H., Zhao, T., Parag, T., et al. (2017). A connectome of a learning and memory center in the adult *Drosophila* brain. eLife *6*, e26975.

Temporal, S., Lett, K.M., and Schulz, D.J. (2014). Activity-dependent feedback regulates correlated ion channel mRNA levels in single identified motor neurons. Curr. Biol. *24*, 1899–1904.

Tessier-Lavigne, M., and Goodman, C.S. (1996). The molecular biology of axon guidance. Science *274*, 1123–1133.

Tien, N.W., and Kerschensteiner, D. (2018). Homeostatic plasticity in neural development. Neural Dev. *13*, 9.

Tobin, W.F., Wilson, R.I., and Lee, W.A. (2017). Wiring variations that enable and constrain neural computation in a sensory microcircuit. eLife *6*, e24838.

Tripodi, M., Evers, J.F., Mauss, A., Bate, M., and Landgraf, M. (2008). Structural homeostasis: compensatory adjustments of dendritic arbor geometry in response to variations of synaptic input. PLoS Biol. *6*, e260.

Turrigiano, G.G. (2017). The dialectic of Hebb and homeostasis. Philos. Trans. R. Soc. B Biol. Sci. *372*, 20160258.

Turrigiano, G.G., and Nelson, S.B. (2004). Homeostatic plasticity in the developing nervous system. Nat. Rev. Neurosci. *5*, 97–107.

Venken, K.J.T., Simpson, J.H., and Bellen, H.J. (2011). Genetic manipulation of genes and cells in the nervous system of the fruit fly. Neuron *72*, 202–230.

Vogelstein, J.T., Park, Y., Ohyama, T., Kerr, R.A., Truman, J.W., Priebe, C.E., and Zlatic, M. (2014). Discovery of brainwide neural-behavioral maps via multiscale unsupervised structure learning. Science *344*, 386–392.

Vonhoff, F., and Keshishian, H. (2017). *In Vivo* calcium signaling during synaptic refinement at the *Drosophila* neuromuscular junction. J. Neurosci. *37*, 5511–5526.

Ward, S., Thomson, N., White, J.G., and Brenner, S. (1975). Electron microscopical reconstruction of the anterior sensory anatomy of the nematode Caenorhabditis elegans. J. Comp. Neurol. *160*, 313–337.

Ward, A., Hong, W., Favaloro, V., and Luo, L. (2015). Toll receptors instruct axon and dendrite targeting and participate in synaptic partner matching in a Drosophila olfactory circuit. Neuron *85*, 1013–1028.

White, J.G., Southgate, E., Thomson, J.N., and Brenner, S. (1986). The structure of the nervous system of the nematode Caenorhabditis elegans. Philos. Trans. R. Soc. Lond. B Biol. Sci. *314*, 1–340.

Wickham, H. (2009). ggplot2: Elegant Graphics for Data Analysis (Springer-Verlag).

Wolfram, V., and Baines, R.A. (2013). Blurring the boundaries: developmental and activity-dependent determinants of neural circuits. Trends Neurosci. *36*, 610–619.

Wu, Z., Sweeney, L.B., Ayoob, J.C., Chak, K., Andreone, B.J., Ohyama, T., Kerr, R., Luo, L., Zlatic, M., and Kolodkin, A.L. (2011). A combinatorial semaphorin code instructs the initial steps of sensory circuit assembly in the Drosophila CNS. Neuron *70*, 281–298.

Xiang, Y., Yuan, Q., Vogt, N., Looger, L.L., Jan, L.Y., and Jan, Y.N. (2010). Light-avoidance-mediating photoreceptors tile the Drosophila larval body wall. Nature *468*, 921–926.

Xu, S., Xiao, Q., Cosmanescu, F., Sergeeva, A.P., Yoo, J., Lin, Y., Katsamba, P.S., Ahlsen, G., Kaufman, J., Linaval, N.T., et al. (2018). Interactions between the Ig-superfamily proteins DIP-α and Dpr6/10 regulate assembly of neural circuits. Neuron *100*, 1369–1384.e6.

Yogev, S., and Shen, K. (2014). Cellular and molecular mechanisms of synaptic specificity. Annu. Rev. Cell Dev. Biol. *30*, 417–437.

Yuan, Q., Xiang, Y., Yan, Z., Han, C., Jan, L.Y., and Jan, Y.N. (2011). Light-induced structural and functional plasticity in Drosophila larval visual system. Science *333*, 1458–1462.

Zarin, A.A., Mark, B., Cardona, A., Litwin-Kumar, A., and Doe, C.Q. (2019). A multilayer circuit architecture for the generation of distinct locomotor behaviors in *Drosophila*. eLife *8*, e51781.

Zhang, W., Yan, Z., Jan, L.Y., and Jan, Y.N. (2013). Sound response mediated by the TRP channels NOMPC, NANCHUNG, and INACTIVE in Chordotonal organs of Drosophila larvae. Proc. Natl. Acad. Sci. U S A *110*, 13612–13617.

Zheng, Z., Lauritzen, J.S., Perlman, E., Robinson, C.G., Nichols, M., Milkie, D., Torrens, O., Price, J., Fisher, C.B., Sharifi, N., et al. (2018). A complete electron microscopy volume of the brain of adult Drosophila melanogaster. Cell *174*, 730–743.e22.

Zlatic, M., Landgraf, M., and Bate, M. (2003). Genetic specification of axonal arbors: atonal regulates robo3 to position terminal branches in the Drosophila nervous system. Neuron *37*, 41–51.

Zlatic, M., Li, F., Strigini, M., Grueber, W., and Bate, M. (2009). Positional cues in the Drosophila nerve cord: semaphorins pattern the dorso-ventral axis. PLoS Biol. *7*, e1000135.

STAR★METHODS

KEY RESOURCES TABLE

REAGENT or RESOURCE	SOURCE	IDENTIFIER
Antibodies		
Mouse anti-V5	Invitrogen	Cat# R960-25, Lot 1949337; RRID: AB_2556564
Rabbit anti-mCherry	Novus Biologicals	Cat# NBP2-25157, Lot 102816; RRID: AB_2753204
Rat anti-HA	Roche	Cat# 11867423001, Lot 27573500; RRID: AB_390918
Donkey anti-mouse IgG Alexa Fluor 488	Jackson ImmunoResearch	Cat# 715-545-151; RRID: AB_2341099
Donkey anti-rat IgG Alexa Fluor 647	Jackson ImmunoResearch	Cat# 712-605-153; RRID: AB_2340694
Donkey anti-rabbit IgG Rhodamine RedTM-X (RRX)	Jackson ImmunoResearch	Cat# 711-295-152; RRID: AB_2340613
Chicken anti-GFP	Abcam	Cat# 13970; RRID: AB_300798
Rabbit anti-dsRed	Clontech	Cat# 632496; RRID: AB_10013483
Goat anti-chicken Alexa Fluor 488	Invitrogen	Cat# A11039; RRID: AB_2534096
Goat anti-rabbit Alexa Fluor 568	Invitrogen	Cat# A11011; RRID: AB_143157
Chemicals, Peptides, and Recombinant Proteins		
All-trans-retinal	Toronto Research Chemicals	Cat# R240000
Experimental Models: Organisms/Strains		
Drosophila: w;; attP2 (empty insertion site)	Pfeiffer et al., 2008	N/A
Drosophila: w; iav-GAL4 in VK00014	Bloomington Drosophila Stock Center	Derived from: BDSC: 36360
Drosophila: w;; iav-GAL4	Bloomington Drosophila Stock Center	BDSC: 52273
Drosophila: w; ppk-LexA in attP40	Vogelstein et al., 2014	N/A
Drosophila: w; R26F05-LexA in attP40	Pfeiffer et al., 2010	BDSC: 54702
Drosophila: w; R61D08-LexAp65 in JK22C	Pfeiffer et al., 2010	N/A
Drosophila: w;; R61D08-GAL4 in attP2	Pfeiffer et al., 2008	BDSC: 39272
Drosophila: w;; R72F11-GAL4 in attP2	Pfeiffer et al., 2008	BDSC: 39786
Drosophila: w; R72F11-LexAp65 in JK22C	Pfeiffer et al., 2010	N/A
Drosophila: w;; ppk-LexA in attP2	Vogelstein et al., 2014	N/A
Drosophila: w;; ppk-QF2	Bloomington Drosophila Stock Center	BDSC: 66475
Drosophila: w;; 165-GAL4	Gift from W. Grueber	N/A
Drosophila w; mhc[1]	Gift from N. Brown	N/A
Drosophila: w; 13XLexAop2-IVS-myr::GFP in su(Hw)attP5	Pfeiffer et al., 2012	N/A
Drosophila: w;; UAS-IVS-myr::tdTomato in attP2	Pfeiffer et al., 2010	BDSC: 32221
Drosophila: w, QUAS-syn21-CsChrimson tdTomato_tr p10 in attP18	This study	N/A
Drosophila: w; 13XLexAop2-CsChrimson-tdTomato in attP40	Gift from V. Jayaraman	N/A
Drosophila: w;; 13XLexAop2-CsChrimsontdTomato in VK00005	Gift from V. Jayaraman	BDSC: 82183
Drosophila: w, 10XUAS-Syn21-Chrimson88-tdT-3.1 in attP18	Gift from A. Wong	N/A
Drosophila: w;; 20xUAS-IVS-GCaMP6s 15.641 in attP2	Gift from V. Jayaraman	N/A

(*Continued on next page*)

Continued

REAGENT or RESOURCE	SOURCE	IDENTIFIER
Drosophila: w;; pJFRC97-20XUAS-IVS-GCaMP3-p10 in attP2	Pfeiffer et al., 2012	N/A
Drosophila: w, LexAop2-Syn21-opGCaMP6s in su(Hw)attP8	Gift from Allan Wong	N/A
Drosophila: w; pSW922[260b] (LexAop-TNT)	Gift from B. Dickson	N/A
Drosophila: w; UAS-TNT-E	Sweeney et al., 1995	N/A
Drosophila: w; 13XLexAop2-IVS-Syn21-Shibire-ts1-p10 in su(Hw)attP5	Pfeiffer et al., 2012	N/A
Drosophila: w;; 20XUAS-TTS-Shibire-ts1-p10 in VK00005	Pfeiffer et al., 2012	BDSC: 66600
Drosophila: w;; UAS-Kir 2.1	Bloomington Drosophila Stock Center	BDSC: 6595
Drosophila: w;; pJFRC26-13XLexAop2-IVS-dTrpA1-WPRE in VK00005	Pfeiffer et al., 2010	N/A
Drosophila: w;; UAS-FraRobo	Bashaw and Goodman, 1999	N/A
Drosophila: w, 10xUAS-IVS-myr::smGdP-HA in attP18	Bloomington Drosophila Stock Center	BDSC: 64092
Drosophila: w, 13xLexAop2-IVS-myr::smGdP-V5 in su(Hw)attP8	Bloomington Drosophila Stock Center	BDSC: 64092
Drosophila: w; UAS-bruchpilot (short)-mstraw	Gift from S. Sigrist	N/A
Drosophila: w; UAS-LacZ	Bloomington Drosophila Stock Center	BDSC: 8530
Drosophila: w; UAS-robo-2::HA	Bloomington Drosophila Stock Center	BDSC: 66886
Drosophila: w; UAS-unc-5::HA	Gift from B. Dickson	N/A
Software and Algorithms		
CATMAID	Saalfeld et al., 2009; Schneider-Mizell et al., 2016	https://www.catmaid.org
R	R Core Team, 2015	https://www.r-project.org/
FIJI	Schindelin et al., 2012	https://imagej.net/Fiji
LARA package	Denisov et al., 2013; Ohyama et al., 2013	https://sourceforge.net/projects/salam-hhmi
Multi Worm Tracker	Swierczek et al., 2011	https://sourceforge.net/projects/mwt

RESOURCE AVAILABILITY

Lead Contact

Further information and requests for resources and reagents may be directed to and will be fulfilled by the Lead Contact, Dr. Marta Zlatic (mzlatic@mrc-lmb.cam.ac.uk).

Materials Availability

Fly strains generated in this study are available from the Lead Contact upon request.

Data and Code Availability

The published article includes all datasets generated or analyzed during this study.

EXPERIMENTAL MODEL AND SUBJECT DETAILS

Fly stocks

All animals used in this study are of the *Drosophila melanogaster* species and were kept on fly food at 25°C unless otherwise specified. The fly food composition is as follows: molasses 5.1% v/v, dry yeast 2.04% m/v, corn meal 8.45% m/v, agar 0.75% m/v, Tegosept 0.2% v/v, and propionic acid 0.5% v/v. Animals for optogenetic experiments were kept in the dark on fly food supplemented with all-trans-retinal (Cat. #R240000, Toronto Research Chemicals) to a concentration of 0.5 mM.

All throughout this document, abbreviated names of the fly strains have been used for simplicity. See Table S3 for full genotypes of all experimental flies used in this study. Different driver lines were used to restrict the expression of a given transgene to the neurons of interest. The GAL4/UAS, LexA/LexAop, and QF/QUAS binary expression systems (del Valle Rodríguez et al., 2011) were used interchangeably. The specific expression system used for each experiment is stated where appropriate.

The R72F11 driver was used for transgene expression in Basin interneurons (Ohyama et al., 2015), *iav* or R61D08 for mechanosensory neurons (Kwon et al., 2010; Ohyama et al., 2015), *ppk* for nociceptive neurons (Ainsley et al., 2003), R26F05 for A08a neurons, and *165-GAL4* for dbd neurons (Sales et al., 2019). The *w;; attP2* line has an empty insertion site with no driver and was used as control for some experiments (where indicated). The mutant line *w; mhc[1]/CyO* was used for live imaging (gift from Nick Brown).

The following effector lines were used: *13XLexAop2-IVS-myr::GFP in su(Hw)attP5* (Pfeiffer et al., 2012), *UAS-IVS-myr::tdTomato in attP2* (Pfeiffer et al., 2010), *UAS-FraRobo* (Bashaw and Goodman, 1999), *20xUAS-IVS-GCaMP6s 15.641 in attP2* (gift from V. Jayaraman) (Chen et al., 2013), *13XLexAop2-CsChrimson-tdTomato in VK00005* and *attp40* (gift from V. Jayaraman), *13XLexAop2-IVS-Syn21-Shibire-ts1-p10 in su(Hw)attP5* (Pfeiffer et al., 2012), *QUAS-syn21-CsChrimson-tdTomato_tr-p10 in attP18*, *pSW922[260b]* (LexAop-TNT) (gift from B. Dickson), *13XLexAop2-IVS-dTrpA1-WPRE in VK00005* (Pfeiffer et al., 2010), *UAS-TNT-E* (Sweeney et al., 1995), *20XUAS-TTS-Shibire-ts1-p10 in VK00005* (Pfeiffer et al., 2012), *UAS-Kir-2.1* (Baines et al., 2001), *UAS-LacZ* (BDSC #8529), *UAS-unc-5::HA* (gift from B. Dickson), *UAS-robo-2::HA* (BDSC #66886).

METHOD DETAILS

Live imaging

For live imaging experiments, fly stocks were generated to label Basin interneurons with myristoylated GFP using the *72F11-LexA* driver, and the mechanosensory neurons with myristoylated tdTomato using the *iav-GAL4* driver. These animals contained a mutation in the myosin heavy chain (*mhc[1]*) that disables muscle contraction in homozygous mutants in order to prevent interference during the imaging process (Mogami and Hotta, 1981; O'Donnell and Bernstein, 1988; Vonhoff and Keshishian, 2017). This mutation was kept over the balancer CyO to establish viable stocks. When possible, CyO labeled with dfd-GMR-Yellow fluorescent protein (DGY) (Le et al., 2006) was used to facilitate the selection of homozygous embryos. For simultaneous live imaging of Basin interneurons and mechanosensory neurons, the following line was used: *w; R72F11-LexAp65 in JK22C, 13XLexAop2-IVS-myr::GFP in su(Hw)attP5, mhc[1]/CyO, DGY; iav-GAL4, UAS-IVS-myr::tdTomato in attP2.*

Eggs were collected for one hour at 25°C on agar plates with yeast paste. After collection, the eggs were incubated at 25°C for 13 hours. Then the eggs were treated with a 1:1 mixture of water and commercial bleach for five minutes or until the chorion was fully removed. The resulting mixture was passed through a sieve to recover the dechorionated eggs. These were rinsed with distilled water to remove bleach and transferred into a Petri dish. Single embryos were carefully picked under a dissection microscope and placed ventral side up on an oxygen-permeable teflon membrane (Lumox). Such membrane was stretched on a custom-made mount that can hold liquid and fits the microscope stage. Multiple embryos were aligned in a row and fully covered with room temperature distilled water. This was done not more than 10 minutes after the embryos were dechorionated to prevent dehydration.

The imaging setup consisted of a Yokogawa CSU-22 spinning disk confocal field scanner mounted on an Olympus BX51 WI fixed-stage upright compound microscope, with an Evolve EMCCD camera (Photometrics) and a LUMPlanFl 60X/0.9 NA (Olympus) water dipping objective. The excitation wavelengths for imaging GFP and tdT were 488 nm and 561 nm, respectively. 50 μm Z stacks with a 1 μm step size and 218 nm/pixel resolution were acquired in two imaging channels every time point for each embryo. The time point frequency varied from 1 to 5 min depending on the number of embryos imaged simultaneously in each session. The center of the stack in the Z axis was roughly located at the center of the developing ventral nerve cord at the beginning of the imaging session. The imaging range in the Z axis was manually readjusted during the session if needed to ensure coverage of the neurons of interest. The images were acquired with the control of MetaMorph software (Molecular devices).

Calcium imaging with GCaMP

Calcium responses were imaged as GCaMP6s (Chen et al., 2013) fluorescence fluctuations in Basin interneurons. CsChrimson was expressed in presynaptic neurons (mechanosensory or nociceptive neurons) for optogenetic activation (Klapoetke et al., 2014). GCaMP signals were recorded in dissected central nervous systems in a saline solution (135 mM NaCl, 5 mM KCl, 2 mM $CaCl_2 \cdot 2H_2O$, 4 mM $MgCl_2 \cdot 6H_2O$, 5 mM TES, 36 mM Sucrose, pH 7.15) and adhered by the ventral side to a cover glass coated with poly-L-lysine (SIGMA, P1524) on a small Sylgard (Dow Corning) plate.

The calcium imaging experiments were performed using a 3i VIVO Multiphoton upright microscope (Intelligent Imaging Innovations). The mechanosensory neurons were photo-stimulated using a 1040 nm laser (1040-3 femtoTrain, Spectra-Physics) coupled to a 2-photon Phasor (Intelligent Imaging Innovations) to generate a holographic pattern to restrict the activation area. GCaMP responses were recorded using an imaging laser tuned to 925 nm (Insight DS+ Dual, Spectra-Physics) and an Apo LWD 25x/1.10W objective (Nikon).

For the reversible silencing of mechanosensory neurons with Shibire^{ts1} (Chen et al., 1991) and recording of Basin interneuron responses the *w; R61D08-LexA; R72F11-GAL4* line was crossed to: *w; LexAop-Shi; UAS-GCaMP6s, LexAop-CsChrimson* for experimental animals, or to *w;; UAS-GCaMP6s, LexAop-CsChrimson* for control. Embryos were collected on retinal food for two hours at 25°C and then incubated in the dark at 31°C for 24 hours, and for another day at 18°C until testing. For the activation of

mechanosensory neurons and recording of Basin interneuron responses, the stimulation protocol consisted of an initial 30 s resting period, a 100 ms stimulation event, and a final 30 s resting period. A photo-stimulation region of 25.1 μm x 10.7 μm was delimited to contain the mechanosensory axon terminals within one abdominal hemisegment, approximately. The stimulation power value measured at the objective end with a power meter (PM100D Thorlabs) was 34.2 mW. This protocol was executed in three different abdominal hemisegments per sample. Any two stimulated ipsilateral hemisegments were separated by at least one unstimulated hemisegment as a precaution. GCaMP responses were imaged at the Basin interneuron axons on a single Z plane at 6.61 frames/s.

For the shifting of mechanosensory neurons by the expression of FraRobo and recording of Basin interneuron responses, the experiments were performed as detailed above with the following modifications. Females of *w, LexAop2-Syn21-opGCaMP6s in su(Hw) attP8, 10XUAS-Syn21-Chrimson88-tdT-3.1 in attP18* and males of *w; R72F11-LexAp65 in JK22C; iav-GAL4, UAS-IVS-myr::tdTomato in attP2* were crossed for control animals. Females of *w, LexAop2-Syn21-opGCaMP6s in su(Hw)attP8, 10XUAS-Syn21-Chrimson88-tdT-3.1 in attP18;; UAS-FraRobo* and males of *w; R72F11-LexAp65 in JK22C; iav-GAL4, UAS-IVS-myr::tdTomato in attP2, UAS-FraRobo* were crossed for experimental animals. Embryos were collected on retinal food for around five hours at 25°C and then incubated in the dark at 25°C for four days until testing. The photo-stimulation was restricted to a region of 22.3 μm x 11.4 μm, and the stimulation event lasted 200 ms. The stimulation power value measured at the objective end with a power meter (PM100D Thorlabs) was 93.0 mW.

For the experiments in which the mechanosensory neurons were silenced with TNT (Sweeney et al., 1995) and the nociceptive neurons were optogenetically activated, the *w; R61D08-LexA; R72F11-GAL4, ppk-QF2* line was crossed to: *w, QUAS-CsChrimson; LexAop-TNT; UAS-GCaMP6s* for experimental animals, or to *w, QUAS-CsChrimson;; UAS-GCaMP6s* for control animals. Eggs were collected on retinal food and incubated in the dark at 25°C for four days. The dissected samples were left in the dark for at least two minutes immediately before initiating the imaging session. All the axons of nociceptive neurons were photo-stimulated with a 625 nm LED mounted on the microscope stage to illuminate the entire sample with 170 $\mu W/cm^2$. The stimulation protocol consisted of an initial 30 s resting period, four 1 s stimulation events of the same intensity, each followed by a 30 s resting period. This protocol was executed once per sample. All other imaging details are as stated above.

Behavioral assays

All the behavioral apparatuses used in this study have been described previously (Ohyama et al., 2013, 2015). Briefly, all rigs had some common core components and differed mostly in the hardware to deliver different types of stimuli. Generally, all consisted of a temperature-controlled enclosure with a high-resolution camera on top, an array of infrared (850 nm) LEDs for illumination, a computer for data acquisition and storage, and the respective hardware modules to deliver and control different stimuli.

For thermogenetic activation, the neurons of interest expressed the heat-activated cation channel TrpA1 (Hamada et al., 2008; Kang et al., 2011). For these experiments, eggs were collected on food plates for 6-8 hours and incubated at 18°C for 8 days, unless otherwise stated. The animals were placed on a thin layer of 4% charcoal agar on top of an aluminum plate. This was placed on a Peltier module to control temperature. The thermogenetic activation protocol consisted of 30 s at 20°C, followed by a ramping-up period of 40 s to reach 35°C, 50 s at 35°C, and a final ramping-down period of 60 s to reach 20°C. Whenever optogenetic activation was paired with a thermal stimulus, red (630 nm) LEDs were used with a power density of 490 $\mu W/cm^2$.

For vibration experiments, eggs were collected on food plates for 6-8 hours and incubated at 25°C for four days, unless otherwise stated. Particularly for those experiments in which the mechanosensory neurons were silenced during development with Shibire^{ts1}, eggs were incubated at 31°C for 24 hours right after collection, and then larvae were incubated at 18°C for another day (for early stage larvae) or 5 days (for late stage larvae) until testing. The mechanical stimulus was delivered as vibration using a speaker located to the side of a 4% agar plate holding the animals. Tones were played at 1000 Hz, with a measured volume (Extech, 407730) of 122 dB. The protocol consisted of 30 s of no sound, 30 s tone at 1000 Hz, and 30 s of no sound.

For optogenetic activation, animals carried the CsChrimson transgene (Klapoetke et al., 2014) in the neurons of interest. Eggs were collected on retinal food for 6-8 hours and incubated in the dark at 25°C for four days, unless otherwise specified. When photo-activation was the only stimulus, larvae were placed on a 4% agar plate on top of an array of red (630 nm) LEDs with power density of 638 $\mu W/cm^2$ through the plate. The activation protocol consisted of 30 s of the LEDs being off, 15 s on, and 30 s off.

For each behavioral experiment, a total of 400-500 animals were tested across multiple trials. For experiments performed on a thermal plate, each trial included approximately 20 animals. All other experimental trials included approximately 50 animals each. The number of animals from experiments that involved young (before 3rd instar) larvae is much lower due to technical difficulties of handling and tracking smaller animals. Many animal traces are discarded throughout the subsequent analysis pipeline. The resulting number of animals used for statistical analysis varies across experiments and depends on the nature of the behavior evoked, stimulus and size of behavioral plate.

As homologous approaches to manipulate activity, the mechanosensory neurons we silenced with the targeted expression of TNT (Sweeney et al., 1995), Shibire^{ts1} (Kitamoto, 2001) or Kir2.1 (Baines et al., 2001; Johns et al., 1999), as indicated.

Stimulus control, object detection, and feature extraction were performed by the Multi Worm Tracker and SALAM-LARA (https://sourceforge.net/projects/salam-hhmi) software as previously described (Denisov et al., 2013; Ohyama et al., 2013; Swierczek et al., 2011).

Electron microscopy volumes and reconstruction

Four electron microscopy volumes were used in this study. They comprise a whole or partial central nervous system of first instar *Drosophila* larvae. Two of these are control volumes which have been previously reported (Ohyama et al., 2015): a whole-central nervous system (CNS) volume (A1 segment, Control 1) and a 1.5-segment long volume (A2/A3 segment, Control 2). Some neurons from the control volumes were previously reconstructed by members and collaborators of the Cardona lab (Janelia Research Campus, HHMI). Control 2 volume had a gap in sections that prevented the complete reconstruction of Griddle, Drunken, and Ladder interneurons, but allowed complete reconstruction of Basin dendrites. The mechano > FraRobo and mechano > TNT EM volumes were acquired for this study using the same preparation and imaging protocols reported for the control volumes (Ohyama et al., 2015). These volumes include a 1.5-segment fraction of the central nervous system (A1/A2 segment) of 1st instar larvae. The genotypes for these volumes are: 1) *w;; iav-GAL4/UAS-FraRobo* 2) *w; UAS-TNT/+; iav-GAL4/+*. They have an image resolution of 3.8 nm by 3.8 nm by 40 nm in x, y and z, respectively. The neurons of interest were reconstructed using CATMAID (Saalfeld et al., 2009) to obtain the skeletonized structure and connectivity of the cells of interest. The neuronal reconstruction process has been detailed previously (Ohyama et al., 2015; Schneider-Mizell et al., 2016).

Identification of manipulated neurons of interest in electron microscopy images

The wild-type morphology and connectivity of all neuron types analyzed in this manuscript have been previously reported (Jovanic et al., 2016; Ohyama et al., 2015). These neurons are uniquely identifiable, both in the wild-type samples and in the experimental samples with silenced or shifted mechanosensory neurons, based on a combination of the following key morphological features (Jovanic et al., 2016; Ohyama et al., 2015): i) the nerve entry point of the main neurite into the neuropil; ii) the growth pattern of the main axonal and dendritic branches in the neuropil on the way to their target area; iii) whether or not the neuron has bilateral or ipsilateral projections; iv) the position of the terminal projections within the medio-lateral, dorso-ventral and antero-posterior axes of the neuropil.

In in our experimental mechano > FraRobo sample, the mechanosensory neurons and their partner interneurons had one of these features altered due to the manipulation (feature (iv), the position of the terminal projections within the medio-lateral axis of the neuropil). Nevertheless, the other features were sufficient to uniquely identify all neurons (see Figure S2 for images of reconstructed whole neurons). The neurons most affected in this sample are the mechanosensory neurons themselves. However, they are distinguishable from all other sensory neurons based on features (i) and (ii). Thus, exactly eight mechanosensory chordotonal axons per hemisegment enter the neuropil at specific and stereotypic points. Mechanosensory chordotonal axons are normally the most lateral sensory neurons in the neuropil that enter in the same nerve bundle. FraRobo expression shifted them even more laterally.

All of the local interneurons can also be uniquely distinguished based on a combination of the features mentioned above. For example, even though the dendrites may look similar (Figures 3E–3H), Ladder and Drunken are uniquely distinguishable based on the following features of their axonal morphologies (Figures 4C–4F): i) neuropil entry point; ii) growth pattern of the main neurite in the neuropil; iii) bilateral versus ipsilateral axonal projection.

Drunken neurite enters the neuropil dorsally and laterally (i) and projects along the dorsal edge before turning ventrally at the midline and then looping back toward the more lateral domain of the neuropil (ii) and has an ipsilateral projection (iii).

Ladder axons enter the neuropil ventrally and medially (i) and then extend laterally on both sides of the midline (ii) making bilateral projections (iii).

Basins can also be distinguished based on these same features: the main neurite enters the neuropil laterally (i) and extends medially on the way to target area (ii) and terminate in the medial region of the neuropil (iv).

Griddle axons enter the neuropil centrally and laterally (i) and project medially toward the midline before looping slightly ventrally and laterally (ii) to terminate in the ventral intermediate domain of the contralateral hemisegment. They are bilateral (iii).

Immunohistochemistry

Third instar larval brains were dissected in PBS, mounted on 12mm #1.5 thickness poly-L-lysine coated coverslips (Neuvitro Corporation, Vancouver, WA, Cat# H-12-1.5-PLL) and fixed for 23 minutes in fresh 4% paraformaldehyde (PFA) (Electron Microscopy Sciences, Hatfield, PA, Cat. 15710) in PBST. Brains were washed in PBST and then blocked with 2.5% normal donkey serum and 2.5% normal goat serum (Jackson ImmunoResearch Laboratories, Inc., West Grove, PA) in PBST overnight. Brains were incubated in a primary antibody mix of mouse anti-V5 tag (Invitrogen, Carlsbad, CA, Cat. R96025, Lot 1949337; 1:1000), rabbit anti-mCherry (Novus Biologicals, Littleton, CO. Cat. NBP2-25157, Lot 102816; 1:500), and rat anti-HA tag (Roche Holding, AG, Basel, Switzerland, Cat. 11867423001, Lot 27573500; 1:100, after suggested dilution) for two days at 4°C. The primary antibodies were removed, and the brains were washed with PBST, then incubated in a secondary antibody mix of Alexa Fluor 488 AffiniPure Donkey Anti-Mouse IgG (H + L) (Jackson ImmunoResearch, West Grove, PA, Cat. 715-545-151; 1:400), Alexa Fluor 647 AffiniPure Donkey Anti-Rat IgG (H + L) (Jackson ImmunoResearch, West Grove, PA, Cat. 712-605-153; 1:400), and Rhodamine RedTM-X (RRX) AffiniPure Donkey Anti-Rabbit IgG (H + L) (Jackson ImmunoResearch, West Grove, PA, Cat. 711-295-152; 1:400) overnight at 4°C. The secondary antibodies were removed following overnight incubation and the brains were washed in PBST. Brains were dehydrated with an ethanol series (30%, 50%, 75%, 100%, 100%, 100% ethanol; all v/v, 10 minutes each) (Decon Labs, Inc., King of Prussia, PA, Cat. 2716GEA) then incubated in xylene (Fisher Chemical, Eugene, OR, Cat. X5-1) for 2x 10 minutes. Samples were mounted onto slides containing DPX mounting medium (Millipore Sigma, Burlington, MA, Cat. 06552) and cured for 3 days then stored at 4°C until imaged.

For the staining of the mechanosensory and Basin neurons, the samples were processed as described above with the following exceptions. Dissected brains were incubated overnight at 4°C in a mix of primary antibodies consisting of chicken anti-GFP (abcam #13970, 1:1000) and rabbit anti-dsRed (Clontech #632496, 1:200). The mix of secondary antibodies consisted of goat anti-chicken Alexa Fluor 488 (AF488) (Invitrogen #A11039, 1:200) and goat anti-rabbit AF568 (Invitrogen #A11011, 1:200). The samples were washed as described above after each antibody incubation. These samples were not processed with ethanol or xylene. The brains were then transferred into 50% EverBrite mounting medium (Biotium #23001) for 30 minutes and then into 100% EverBrite overnight at 4°C.

QUANTIFICATION AND STATISTICAL ANALYSIS

Image processing for live imaging data

Standard image processing was performed using Fiji (Schindelin et al., 2012). Briefly, the imaging stacks were cropped to remove Z sections that did not contain the neurons of interest. The images were denoised using nd-safir (Boulanger et al., 2010). Z-projections were generated, and the imaging channels were merged to create 2D time-lapse videos of the developing neurons in two colors. Bleach correction (Fiji) was used to adjust for the increasing brightness of the neurons through time. Ilastik (Sommer et al., 2011) was used for pixel classification to generate segmented images. Different trained pixel-classification parameters were used for each imaging channel.

Image analysis of calcium imaging data

The GCaMP image data were processed using custom macros in Fiji (Schindelin et al., 2012) and analyzed using custom code written in R (R Core Team, 2015). Briefly, a region of interest (ROI) was manually defined to include the corresponding GCaMP-expressing axons. The average pixel value inside such ROI was measured with Fiji across all time points for each sample. All fluorescence values were reported relative to a fluorescence baseline (F_0) defined as the median pixel value of the corresponding ROI during the entire imaging experiment. $\Delta F/F_0$ was calculated as $\Delta F/F_0 = (F_t - F_0)/F_0$, where F_t is the mean fluorescence value of the ROI at a given time point. The relative maximum $\Delta F/F_0$ was defined in a 4.5 s time window immediately after stimulation offset from which the recent baseline (mean $\Delta F/F_0$ of the 3 s preceding stimulation onset) was subtracted. Those failed individual trials in which there were no detectable responses were discarded. A trial with no response was defined as that in which the mean $\Delta F/F_0$ in the 4.5 s following stimulation was within ± 1.5 (for mechanosensory neurons) or ± 0.5 (for nociceptive neurons) standard deviations of the recent baseline (3 s preceding stimulation). Individual imaging trials were averaged by animal. The calcium imaging data were plotted using the ggplot2 (Wickham, 2009) package in R.

Electron microscopy connectivity analysis

All synaptic connections in this study represent chemical synapses. All the connectivity data were generated in CATMAID and processed in R. For all connectivity quantifications, individual neurons of the same cell type in one hemisegment were grouped to include: 8 mechanosensory axons, 6 Ladder interneurons, 4 Basin interneurons, 3 nociceptive axons, one Griddle-2 interneuron, or one Drunken-1 interneuron. Since Ladder interneurons are bilateral with medial cell bodies, the difference between their left and right connectivity resides in their presynaptic connections, which come from unilateral neurons.

The fraction of mechanosensory input onto preferred partners (number of synapses from mechanosensory neurons to partner A, divided by the total number of inputs of partner A) was compared between EM volumes. Basin, Drunken, and Griddle interneurons receive most of their mechanosensory input onto their dendrites, so we calculated mechanosensory inputs relative to the total amount of input onto their dendrites (Tables S1 and S2), which are mostly contained in the mechano > FraRobo and mechano > TNT EM volumes. However, Ladder interneurons normally receive significant mechanosensory input onto dendrites and axons, so we calculated the fraction of mechanosensory input from their total (axonal and dendritic) input synapses (Tables S1 and S2). Since parts of Ladder arbors exited the mechano > FraRobo and mechano > TNT EM volumes (1.5 segments), equivalent (in coverage) subvolume limits were used to restrict the total number of Ladder synapses considered from the wild-type volume (whole CNS). This correction made it possible to compare Ladder connectivity between wild-type and experimental volumes.

The calculation of connectivity likelihood between mechanosensory neurons (in A1 segment of whole-CNS WT volume) and homolog neurons in the right and left sides of the same segment included only those partners that had been previously identified and were at least 500 nodes in length. This filter returned 160 and 149 partners for the left and right side, respectively. The average of the left and right calculations is reported.

Node and synapse distribution of reconstructed neurons

Node density was quantified using a 2.5 μm sliding window along the mediolateral axis. The mediolateral positions were normalized to the width of the neuropil of the corresponding EM volume and centered at the midline. The node densities were normalized to the maximum density of the respective cell type. Density plots were smoothed using the loess function in R with a span of 0.1.

CellPress
OPEN ACCESS

Statistical analysis

Statistical analyses were performed using R. All statistical tests, significance levels, number of observations and other relevant information for data comparisons are specified in the respective figure legend and below. In all figures, * represents p value < 0.05, ** represents p value < 0.01, and *** represents p value < 0.001.

The calcium responses between control and experimental animals were compared using the single-sided Wilcoxon rank-sum test.

For the behavioral assays, the probability of a behavior occurring was calculated as the proportion of animals that performed the specified behavior at least once during the 15 s (for optogenetic activation or vibration stimulus) or 40 s (for thermogenetic activation) immediately after stimulus onset across all trials. The analysis time window for thermogenetic activation is longer due to its slower activation resulting from temperature ramping. Therefore, the stimulus onset for thermogenetic activation experiments was defined as the moment the thermal plate reached 35°C. Only those animals that were detected for at least 95% of the analyzed time window and did not come into contact with another animal during this period were included in the analysis. The behavior probabilities were compared using a chi-square test for proportions. Behavior durations were calculated for the time windows mentioned above and compared using a double-sided t test.

Electron microscopy connectivity was computed as the fraction of postsynaptic input, unless otherwise specified. Connectivity data were compared using a chi-square test for proportions.

Quantification of mechanosensory, Basin and A08a membrane distribution

Image processing and analysis was performed using FIJI (Schindelin et al., 2012). Stepwise, images were rotated (Image > Transform > Rotate(bicubic)) to align dendrites of interest along the x axis, then a region of interest was selected in 3D to include the dendrites to analyze in one hemisegment (Rectangular selection > Image > Crop). To identify the voxels that contain dendrite intensity, a mask was manually applied (Image > Adjust > Threshold). The threshold was assigned to include dendrite positive voxels and minimize contribution from background. To quantify the amount of dendrite positive voxels across the medial-lateral axis, images were reduced in the Z-dimension (Image > Stacks > Z-project > Sum Slices) and a plot profile was obtained to measure the average voxel intensity (Rectangular selection > Analyze > Plot profile).

Neuron, Volume *109*

Supplemental Information

Comparative Connectomics Reveals How Partner Identity, Location, and Activity Specify Synaptic Connectivity in *Drosophila*

Javier Valdes-Aleman, Richard D. Fetter, Emily C. Sales, Emily L. Heckman, Lalanti Venkatasubramanian, Chris Q. Doe, Matthias Landgraf, Albert Cardona, and Marta Zlatic

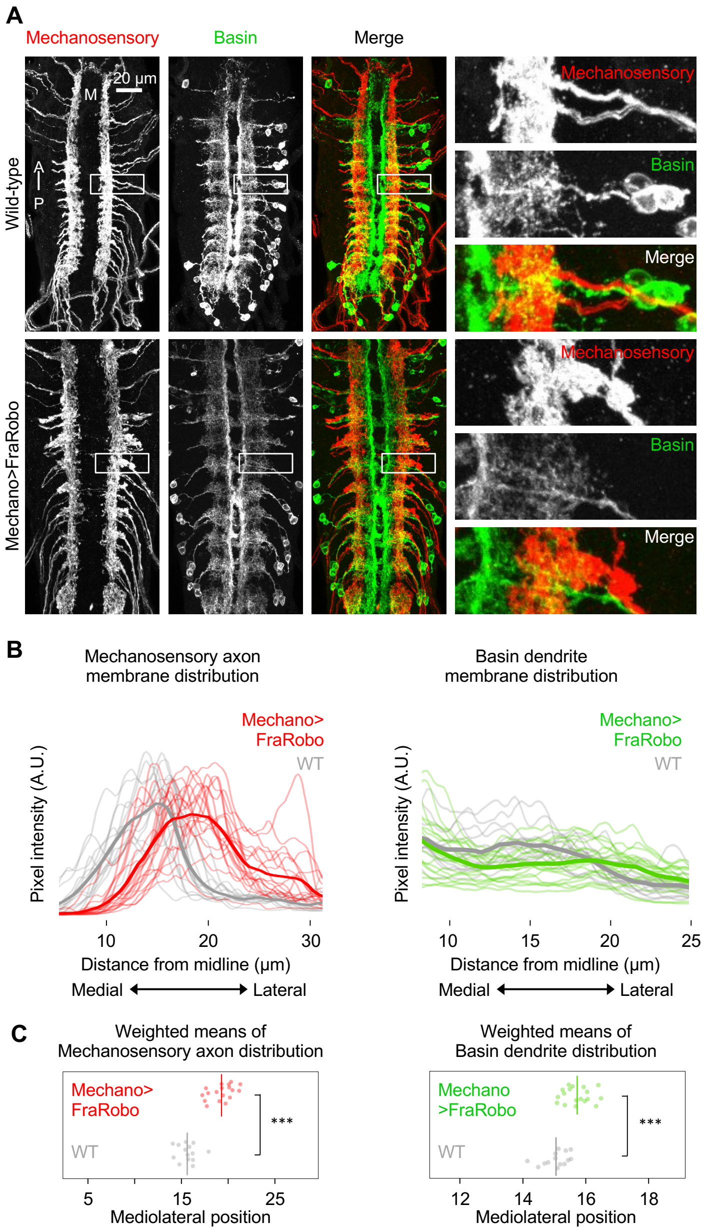

Figure S1. Light imaging of displacement of mechanosensory neuron axons and postsynaptic Basin dendrites. Related to Figure 3.

A) Representative Z-projections of confocal images of mechanosensory neurons and Basin interneurons in the nerve cord of 3rd instar larvae expressing FraRobo in mechanosensory neurons (mechano>FraRobo) and wild-type. White squares enlarged to the right. M, midline.

B) Pixel distribution plots for mechanosensory axons and Basin interneurons in a hemisegment. Left and right sides of multiple segments were compiled in a single hemisegment representation. Bold lines represent mean traces, faded lines represent individual hemisegments. n= 14 hemisegments for wild-type; n= 18 hemisegments for mechano>FraRobo.

C) Weighted mean of the distribution of mechanosensory axons and Basin dendrites. The mediolateral range considered for quantification is shown in B. n= 14 hemisegments for wild-type; n= 18 hemisegments for mechano>FraRobo. Data compared using an unpaired t-test. ***, $P < 0.001$.

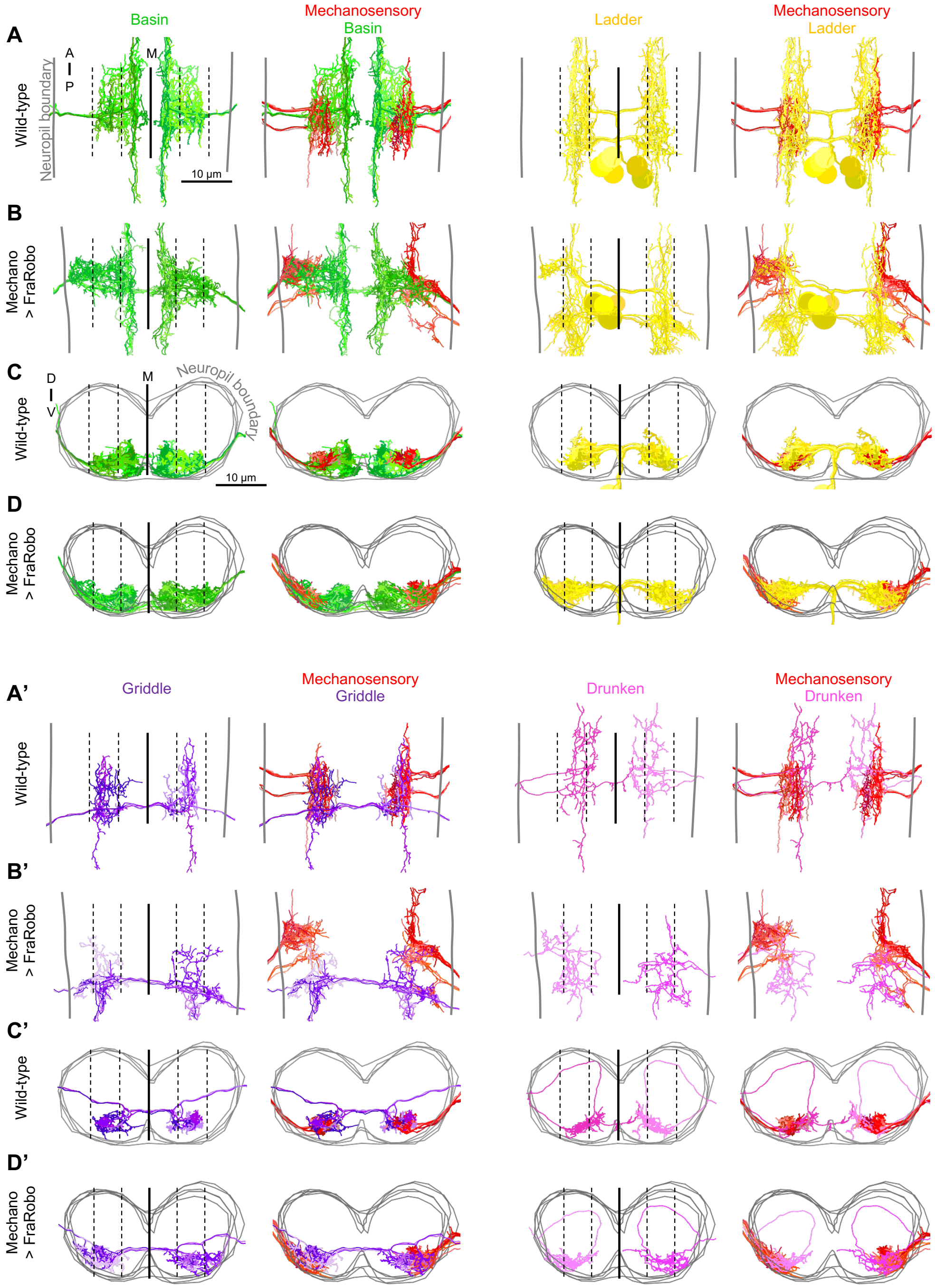

Figure S2. Full neuron (axon, dendrites and neuropil entry point) view of partner cells and their overlap with mechanosensory neurons. Related to Figures 3 and 4.

A-D') Dorsal (A-B') and cross section (C-D') views of the mechanosensory neurons and their preferred partners in wild-type (A, A', C and C') and the mechano>FraRobo (B, B', D and D') volumes. The neuropil boundary is represented by either a pair of gray vertical lines for dorsal views (A-B') or gray consecutive rings for cross section views (C-D'). Dashed lines split the maximum width of the neuropil in six equidistant sections, three on either side of the midline (M).

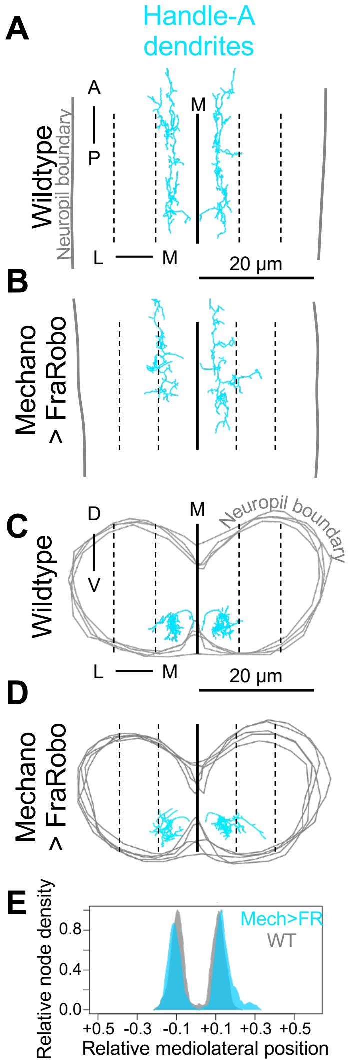

Figure S3. Displacement of mechanosensory axons does not affect the position of non-partner neurons. Related to Figures 3 and 4.

A-D) Dorsal (A-B) and cross section (C-D) views of the dendrites of Handle-A in wild-type (A and C) and the mechano>FraRobo (B and D) volumes. Handle-A does not receive direct input from the mechanosensory neurons. The mediolateral position of its dendrites is not affected by the lateral displacement of the mechanosensory axons.

E) Node density distribution of Handle-A in the mediolateral axis in wild-type (WT) and mechano>FraRobo (mech>FR).

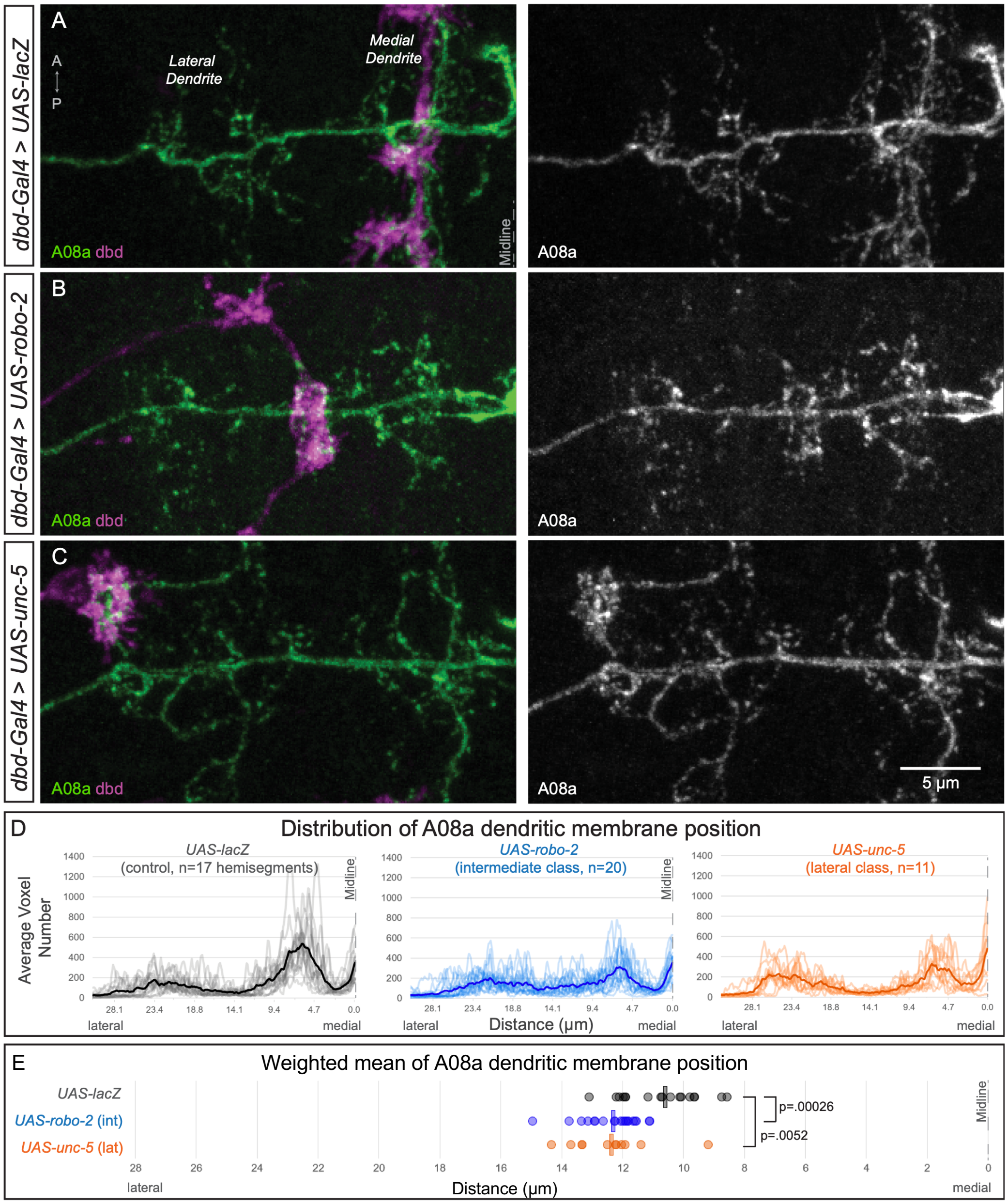

Figure S4. Displacement of dbd sensory neuron axons causes their postsynaptic partner dendrites to follow. Related to Figure 3.

A-C) Confocal maximum intensity projection of the dorsal view of dbd axon terminal (magenta) and the A08a dendritic domain (green) in one hemisegment of a 3rd instar larva. Merged channels shown to the left; A08a channel shown to the right.

A) In wild-type, dbd axon targets the A08a medial arbor. n=17 hemisegments from 11 animals.

B) dbd axons expressing Robo-2 are shifted laterally and often contact the A08a intermediate domain. n=20 hemisegments from 10 animals.

C) dbd expressing Unc-5 often contact the A08a lateral arbor. n=11 hemisegments from 10 animals.

D) Quantification of the distribution of A08a dendrite position in the context of medial, intermediate, and lateral dbd axons. Transparent lines represent individual hemisegments and each solid line represents the average for the cohort.

E) Weighted mean of the dendrite distributions shown in D. Each circle represents one hemisegment and bars represent the average weighted mean for each cohort. Average weighted mean: *UAS-lacZ*: 10.63 µm; *UAS-robo-2*, intermediate: 12.33 µm; *UAS-unc-5*, lateral: 12.38 µm. P-values were obtained using an unpaired t-test.

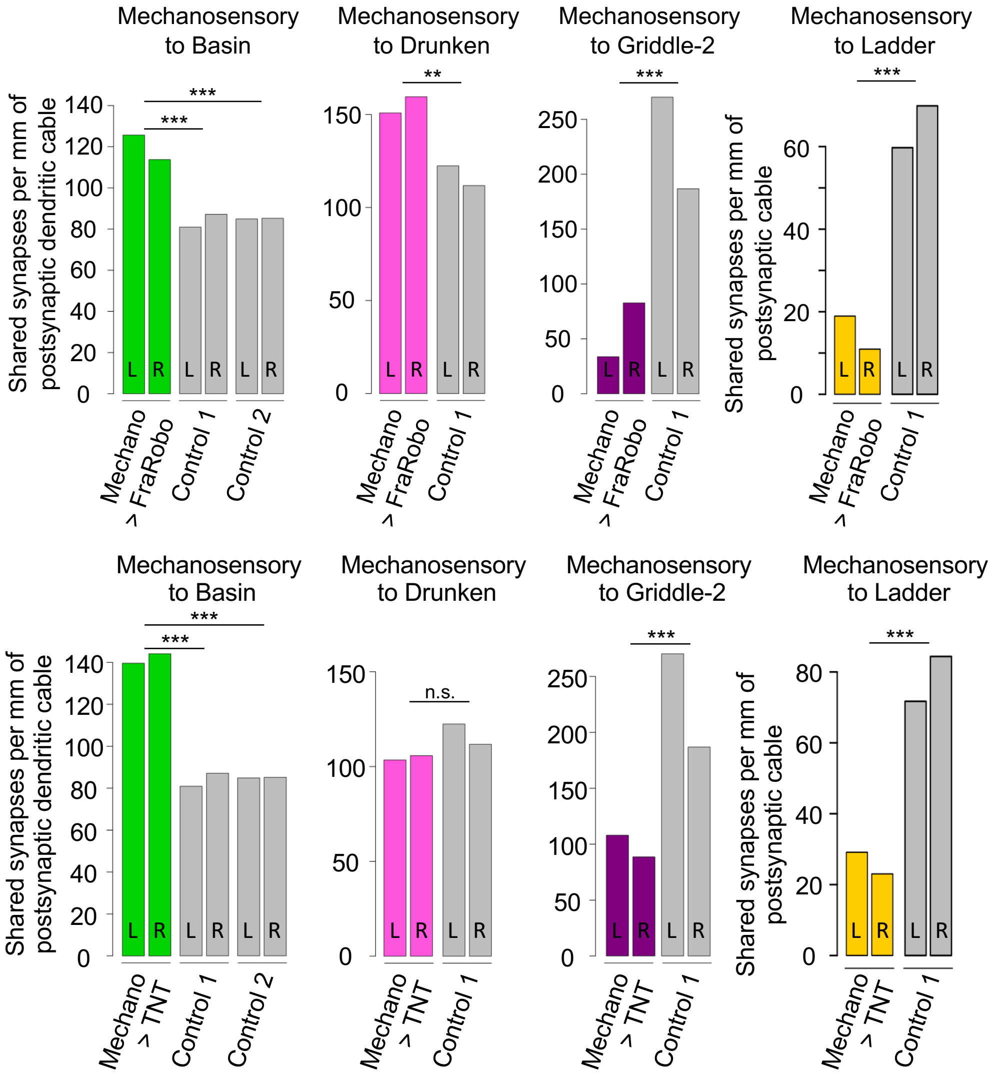

Figure S5. Connectivity relative to cable length between mechanosensory neurons and their preferred postsynaptic partners in the mechano>FraRobo and mechano>TNT EM volumes. Related to Figures 5 and 6.

Number of synapses from mechanosensory neurons onto postsynaptic partners per mm of cable length. Chi-square test. **, P < 0.01, and ***, P < 0.001.

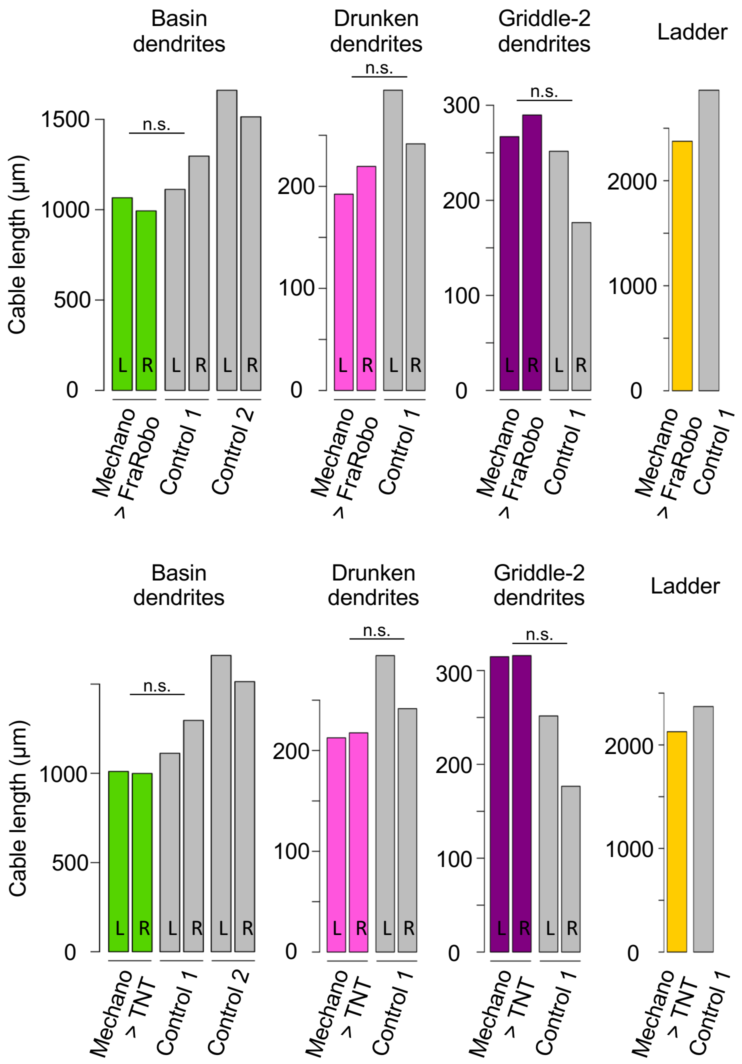

Figure S6. Cable length of reconstructed interneurons in the mechano>FraRobo and mechano>TNT EM volumes. Related to Figures 5, 6 and 7.

Total linearized distance of the reconstructed neurons of interest in the left (L) and right (R) hemisegments of the mechano>FraRobo, mechano>TNT, control-1 (1st instar), control-2 (older 1st instar) EM volumes. Axonal and dendritic distance shown for Ladder. There are no left and right homologous cells for ladder, as their cell bodies are medial with symmetrical bilateral projections. The quantification of the cable length for ladder in control-1 is adjusted based on the size and position of the corresponding control subvolume it is compared to (see Methods). Cable length compared with t-test, n=2 hemisegments.

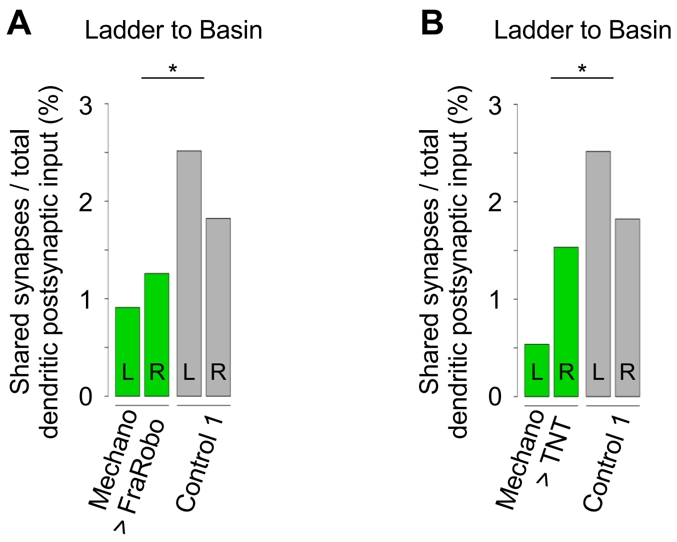

Figure S7. Connectivity between interneurons is decreased in the mechano>FraRobo and mechano>TNT EM volumes. Related to Figures 5, 6 and 7.

The fraction of input from inhibitory Ladders onto Basin interneurons is decreased when the mechanosensory neurons were shifted by the expression of FraRobo (A) or silenced by the expression of TNT (B). This reduction in connectivity between Ladder and Basin interneurons may be at the expense of the increase in connections between sensory neurons (mechanosensory and/or nociceptive) and Basin interneurons under the same conditions (Figures 5E, 6B and 7A). Chi-square test. *, $P < 0.05$.

Direction	mechano-FraRobo	mechano-TNT	WT-1	WT-2
Mechano_to_Basin_L	134	141	90	141
Mechano_to_Basin_R	113	144	113	129
Mechano_to_Noci_L	0	0	0	NA
Mechano_to_Noci_R	0	0	0	NA
Mechano_to_Drunken-1_L	29	22	36	NA
Mechano_to_Drunken-1_R	35	23	27	NA
Mechano_to_Griddle-2_L	9	34	68	NA
Mechano_to_Griddle-2_R	24	28	33	NA
Mechano_to_Ladder_L	45	62	171	NA
Mechano_to_Ladder_R	26	49	200	NA
Noci_to_Basin_L	NA	52	51	NA
Noci_to_Basin_R	NA	61	57	NA
Ladder_to_Basin_L	6	3	23	NA
Ladder_to_Basin_R	7	9	19	NA

Table S1. Connectivity data. Related to Figures 5E-H, 6B-E and 7A.

Synapse counts between mechanosensory or nociceptive neurons and their preferred partners. Connectivity from the mechano>FraRobo and mechano>TNT EM volumes generated for this study, and from the previously reported control-1 (WT-1) and control-2 (WT-2) EM volumes (Jovanic et al., 2016; Ohyama et al., 2015).

Group	Cable length	Presynaptic sites	Postsynaptic sites	Volume	Sub arbor	Side
Mechano	461567.848	430	225	WT-1	axon	L
Mechano	483976.708	437	203	WT-1	axon	R
Nociceptive	226464.851	148	38	WT-1	axon	L
Nociceptive	228380.665	162	47	WT-1	axon	R
Ladder	2370566.76	338	1333	WT-1 (to compare with TNT)	whole	Bi
Ladder	2862200.75	409	1556	WT-1 (to compare with FraRobo)	whole	Bi
Basin	1112276.31	38	914	WT-1	dendrites	L
Basin	1296695.63	32	1042	WT-1	dendrites	R
Griddle-2	251606.121	41	179	WT-1	dendrites	L
Griddle-2	176651.487	38	110	WT-1	dendrites	R
Drunken-1	294221.904	26	206	WT-1	dendrites	L
Drunken-1	241624.37	29	161	WT-1	dendrites	R
Mechano	738345.826	226	133	mechano-FraRobo	axon	L
Mechano	699198.101	202	122	mechano-FraRobo	axon	R
Nociceptive	282349.661	105	54	mechano-FraRobo	axon	L
Nociceptive	287000.292	98	41	mechano-FraRobo	axon	R
Ladder	2375634.7	323	1000	mechano-FraRobo	whole	Bi
Basin	1066024.36	17	659	mechano-FraRobo	dendrites	L
Basin	993480.482	19	556	mechano-FraRobo	dendrites	R
Griddle-2	266985.898	25	135	mechano-FraRobo	dendrites	L
Griddle-2	289732.528	23	174	mechano-FraRobo	dendrites	R
Drunken-1	192320.879	11	136	mechano-FraRobo	dendrites	L
Drunken-1	219498.444	14	131	mechano-FraRobo	dendrites	R
Mechano	485055.1	216	87	mechano-TNT	axon	L
Mechano	361425.439	217	137	mechano-TNT	axon	R
Nociceptive	201983.643	133	59	mechano-TNT	axon	L
Nociceptive	208142.3	106	68	mechano-TNT	axon	R
Basin	1010526.71	12	558	mechano-TNT	dendrites	L
Basin	999269.61	16	587	mechano-TNT	dendrites	R
Ladder	2127555.75	336	901	mechano-TNT	whole	Bi
Griddle-2	314722.78	23	187	mechano-TNT	dendrites	L
Griddle-2	315893.148	22	168	mechano-TNT	dendrites	R
Drunken-1	212620.198	12	123	mechano-TNT	dendrites	L
Drunken-1	217554.555	11	111	mechano-TNT	dendrites	R
Mechano	466430.487	366	201	WT-2	axon	L
Mechano	418329.247	341	200	WT-2	axon	R
Basin	1660816.4	35	1185	WT-2	dendrites	L
Basin	1513899.48	37	1072	WT-2	dendrites	R

Table S2. EM-reconstructed neuron details. Related to Figures 5E-H, 6B-E and 7A.

Cable length (nm), presynaptic and postsynaptic sites of mechanosensory, nociceptive, Basin, Ladder, Griddle-2, and Drunken-1 neurons in mechano>FraRobo, mechano>TNT, control-1 (WT-1), or control-2 (WT-2) EM volumes.

Figure	Details	Genotype
2, Video S1	Live imaging of mechanosensory and Basin neurons	*w; R72F11-LexAp65 in JK22C, 13XLexAop2-IVS-myr::GFP in su(Hw)attP5, mhc[1]; iav-GAL4, UAS-IVS-myr::tdTomato in attP2*
3, 4, 5B-C, 5E-H, S2, S3, S5, S6, S7	Control	*[iso] Canton S G1 × w^{1118} [iso] 5905*
	Experimental	*w;; iav-GAL4/UAS-FraRobo*
5I-I’	Control	*w, LexAop2-Syn21-opGCaMP6s in su(Hw)attP8, 10XUAS-Syn21-Chrimson88-tdT-3.1 in attP18/+; R72F11-LexAp65 in JK22C/+; iav-GAL4, UAS-IVS-myr::tdTomato in attP2, UAS-FraRobo/UAS-FraRobo*
	Experimental	*w, LexAop2-Syn21-opGCaMP6s in su(Hw)attP8, 10XUAS-Syn21-Chrimson88-tdT-3.1 in attP18/+; R72F11-LexAp65 in JK22C/+; iav-GAL4, UAS-IVS-myr::tdTomato in attP2/+*
5J-J’	Control	*w;; iav-GAL4, UAS-IVS-myr::tdTomato in attP2*
	Experimental	*w;; iav-GAL4, UAS-IVS-myr::tdTomato in attP2, UAS-FraRobo*
6B-E, 7A, S5, S6, S7	Control	*[iso] Canton S G1 × w^{1118} [iso] 5905*
	Experimental	*w; UAS-TNT-E/+; iav-GAL4/+*
6G-G’	Control	*w; R61D08-LexAp65 in JK22C/+; 20xUAS-IVS-GCaMP6s 15.641 in attP2, 13XLexAop2-CsChrimson-tdTomato in VK00005/R72F11-GAL4 in attP2*
	Experimental	*w; R61D08-LexAp65 in JK22C/13XLexAop2-IVS-Syn21-Shibire-ts1-p10 in su(Hw)attP5; 20xUAS-IVS-GCaMP6s 15.641 in attP2, 13XLexAop2-CsChrimson-tdTomato in VK00005/R72F11-GAL4 in attP2*
6I-J’	Control	*;; attP2/UAS-Shibire-ts1*
	Experimental	*;; R61D08-GAL4 in attP2/UAS-Shibire-ts1*
7B-B’, D	Control	*w, QUAS-syn21-CsChrimson tdTomato_tr p10 in attP18/+; R61D08-LexAp65 in JK22C/+; R72F11-GAL4 in attP2, ppk-QF2/20xUAS-IVS-GCaMP6s 15.641 in attP2*
	Experimental	*w, QUAS-syn21-CsChrimson tdTomato_tr p10 in attP18/+; R61D08-LexAp65 in JK22C/pSW922[260b] (LexAop-TNT); R72F11-GAL4 in attP2, ppk-QF2/20xUAS-IVS-GCaMP6s 15.641 in attP2*
7D’, F	Control	*w; attP2/13XLexAop2-CsChrimson-tdTomato in attP40; ppk-LexA in attP2, 20XUAS-TTS-Shibire-ts1-p10 in VK00005/+*
	Experimental	*w; R61D08-GAL4 in attP2/13XLexAop2-CsChrimson-tdTomato in attP40; ppk-LexA in attP2, 20XUAS-TTS-Shibire-ts1-p10 in VK00005/+*
7E	Control	*w; UAS-TNT-E/ppk-LexA in attP40; pJFRC97-20XUAS-IVS-GCamp3-p10 in attP2, pJFRC26-13XLexAop2-IVS-dTrpA1-WPRE in VK00005/+*
	Experimental	*w; iav-GAL4, UAS-TNT-E/ppk-LexA in attP40; pJFRC97-20XUAS-IVS-GCaMP3-p10 in attP2, pJFRC26-13XLexAop2-IVS-dTrpA1-WPRE in VK00005/+*
7E’	Control	*w; ppk-LexA in attP40/+; attP2/LexAop-TrpA1 in VK00005, UAS-Kir 2.1*
	Experimental	*w; ppk-LexA in attP40/+; R61D08-GAL4 in attP2/LexAop-TrpA in VK00005, UAS-Kir 2.1*
S1	Control	*w; R72F11-LexAp65 in JK22C, 13XLexAop2-IVS-myr::GFP in su(Hw)attP5 ; iav-GAL4, UAS-IVS-myr::tdTomato in attP2*
	Experimental	*w; R72F11-LexAp65 in JK22C, 13XLexAop2-IVS-myr::GFP in su(Hw)attP5 ; iav-GAL4, UAS-IVS-myr::tdTomato in attP2, UAS-FraRobo*
S4	Control	*w, 10xUAS-IVS-myr::smGdP-HA in attP18, 13xLexAop2-IVS-myr::smGdP-V5 in su(Hw)attP8/+; R26F05-LexA, UAS-bruchpilot (short)-mstraw/UAS-LacZ; 165-GAL4/+*
	Expression of Robo2 in dbd neurons	*w, 10xUAS-IVS-myr::smGdP-HA in attP18, 13xLexAop2-IVS-myr::smGdP-V5 in su(Hw)attP8/+; R26F05-LexA, UAS-bruchpilot (short)-mstraw/UAS-robo-2::HA; 165-GAL4/+*
	Expression of Unc-5 in dbd neurons	*w, 10xUAS-IVS-myr::smGdP-HA in attP18, 13xLexAop2-IVS-myr::smGdP-V5 in su(Hw)attP8/+; R26F05-LexA, UAS-bruchpilot (short)-mstraw/UAS-unc-5::HA; 165-GAL4/+*

Table S3. Fly line genotypes. Related to STAR Methods.

Data S1. Neuronal atlas of mechanosensory neurons and preferred partners in the mechano>FraRobo EM volume. Related to Figures 3-5.

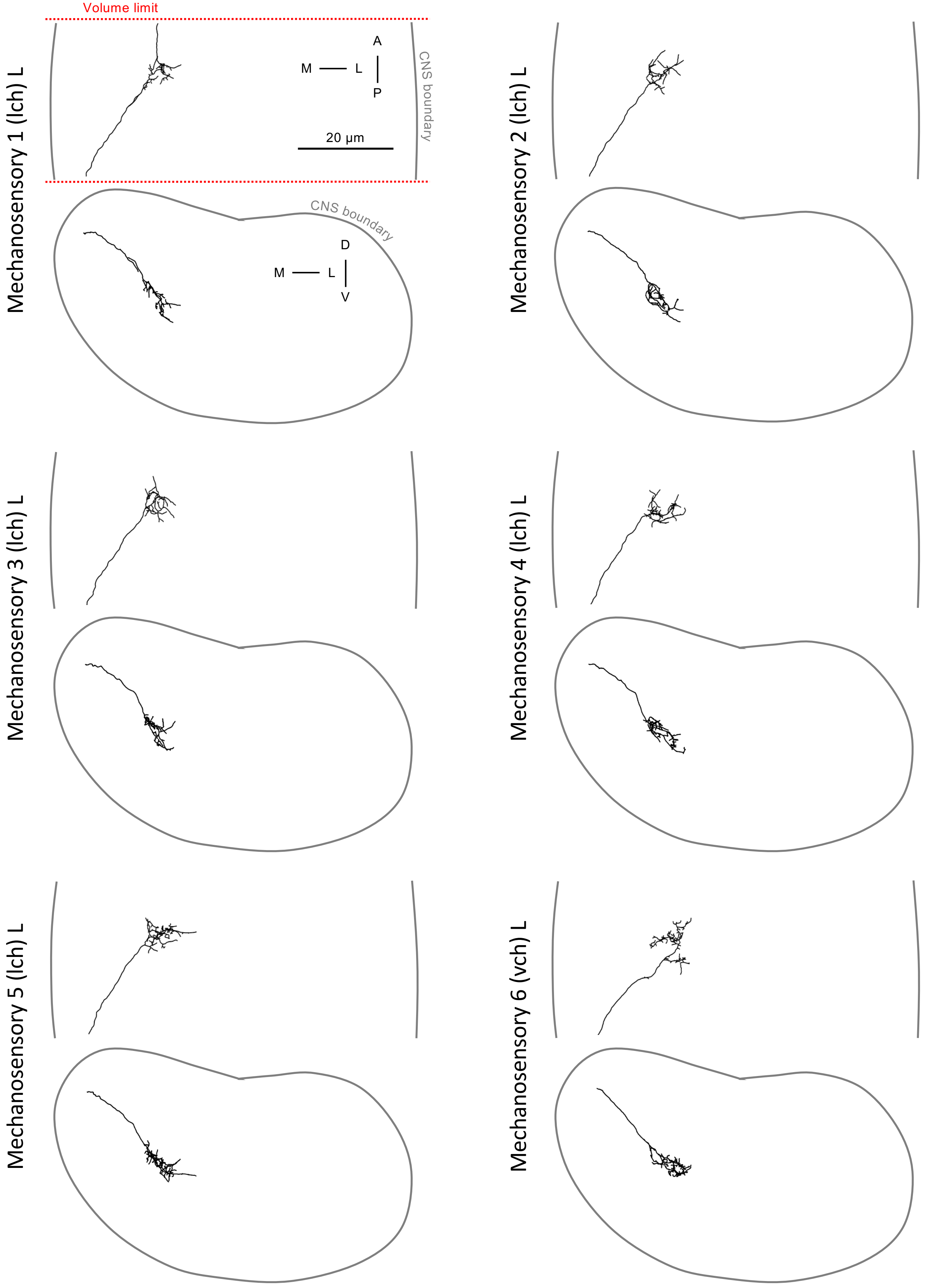

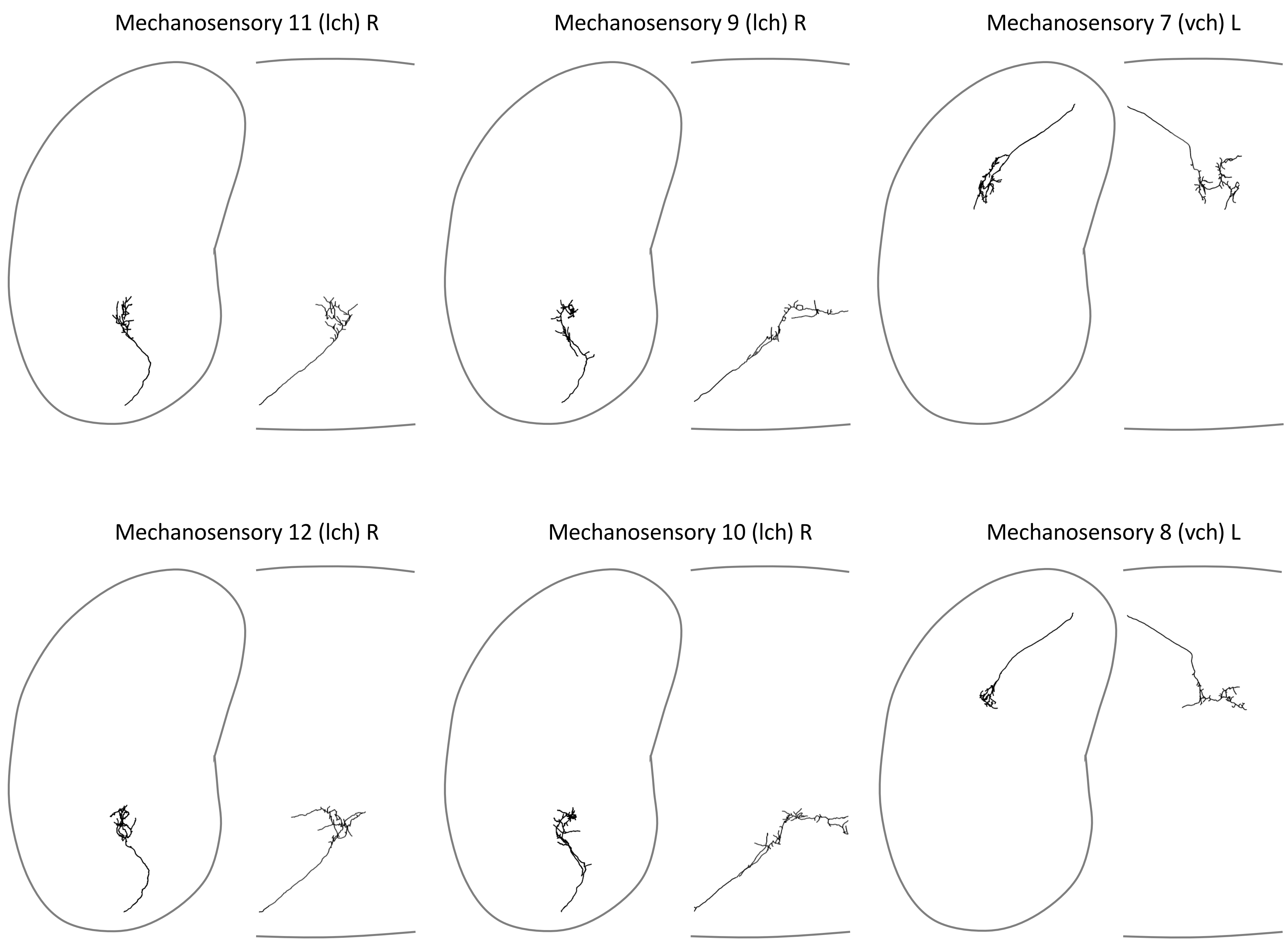
Mechanosensory 11 (lch) R
Mechanosensory 9 (lch) R
Mechanosensory 7 (vch) L
Mechanosensory 12 (lch) R
Mechanosensory 10 (lch) R
Mechanosensory 8 (vch) L

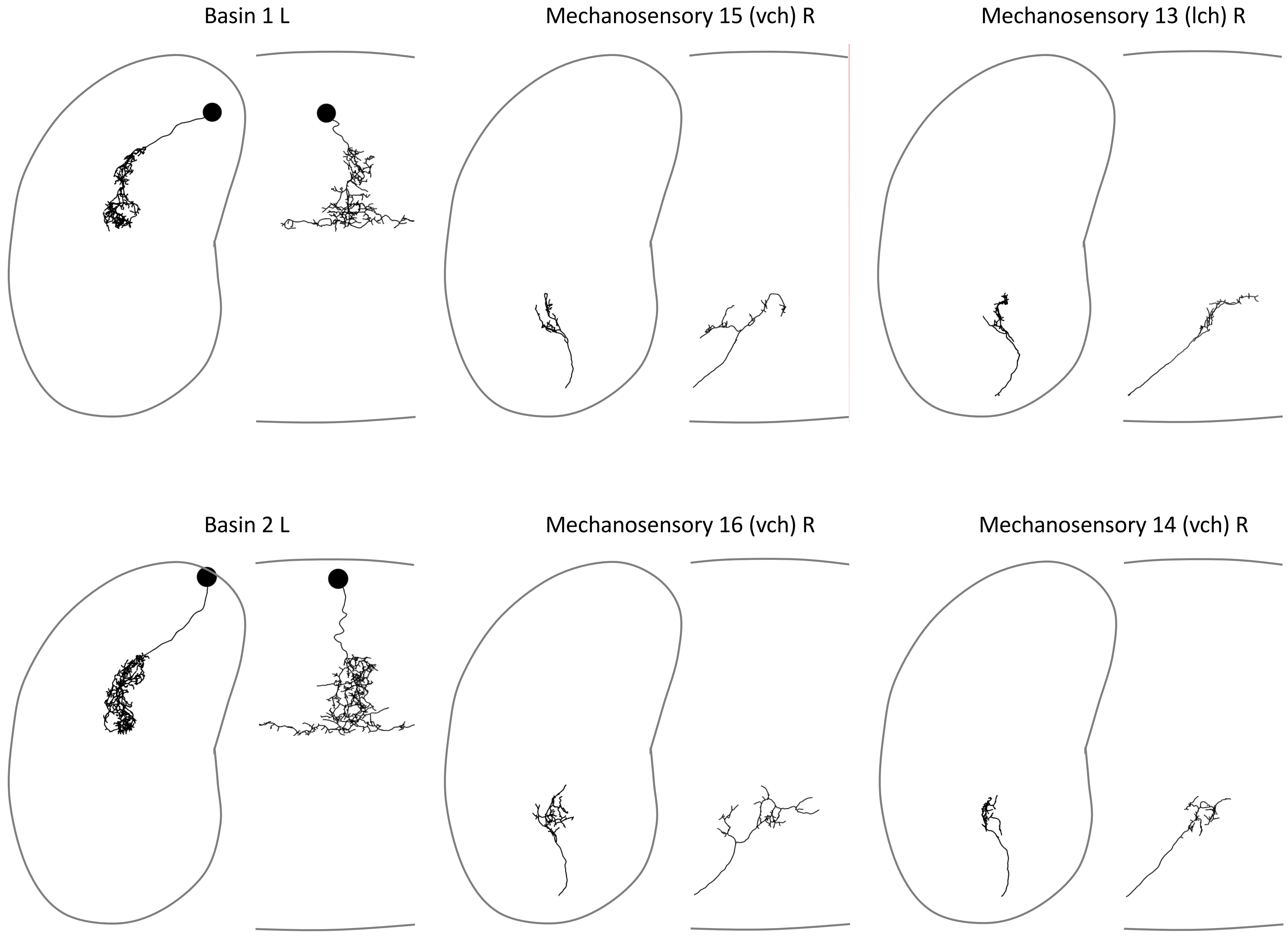
Basin 1 L
Mechanosensory 15 (vch) R
Mechanosensory 13 (lch) R
Basin 2 L
Mechanosensory 16 (vch) R
Mechanosensory 14 (vch) R

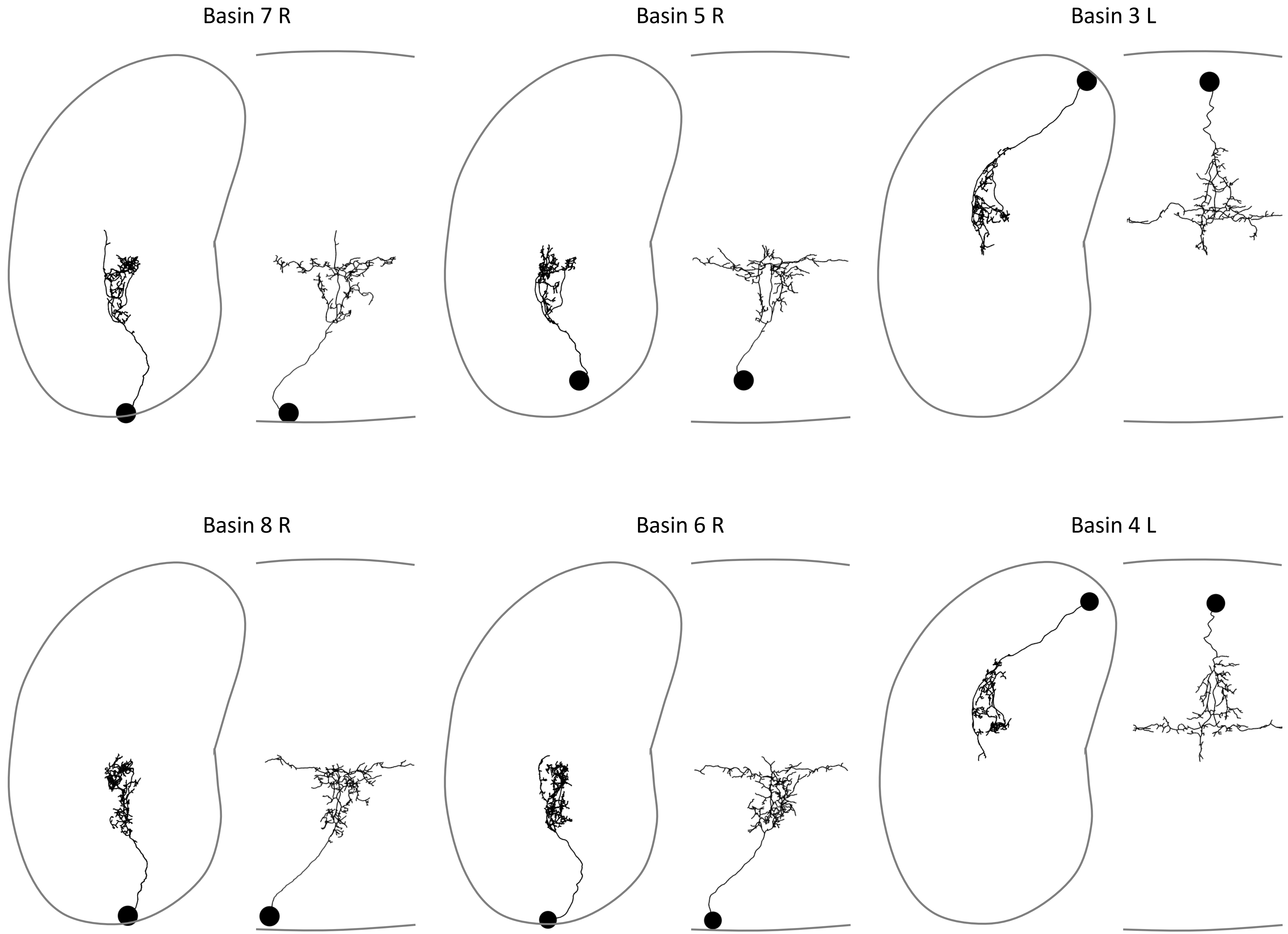
Basin 7 R
Basin 5 R
Basin 3 L
Basin 8 R
Basin 6 R
Basin 4 L

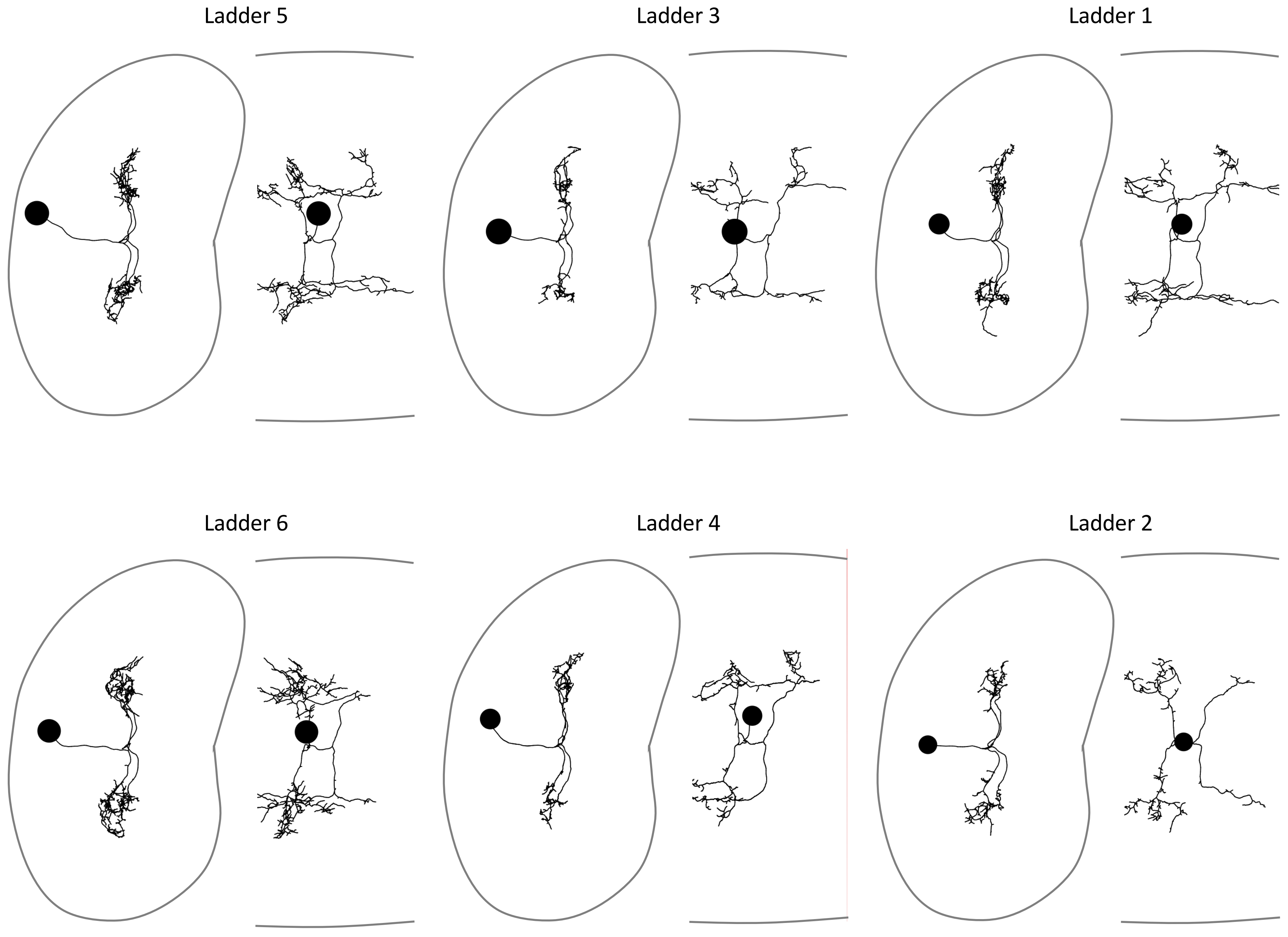

Ladder 5
Ladder 3
Ladder 1
Ladder 6
Ladder 4
Ladder 2

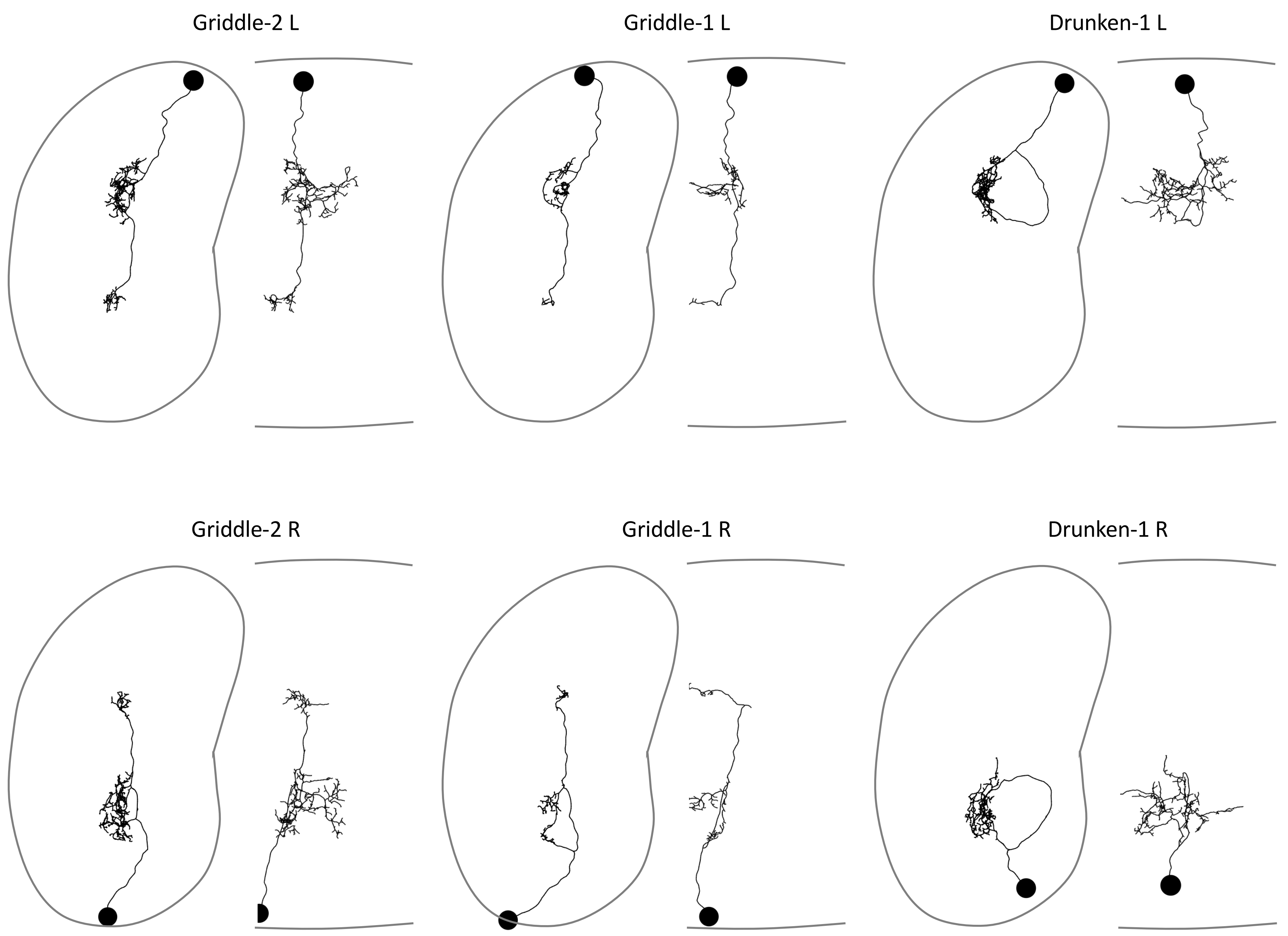
Griddle-2 L
Griddle-1 L
Drunken-1 L
Griddle-2 R
Griddle-1 R
Drunken-1 R

Data S2. Neuronal atlas of "other local" mechanosensory partners in the mechano>FraRobo and control EM volumes. Related to Figure 5B-C.

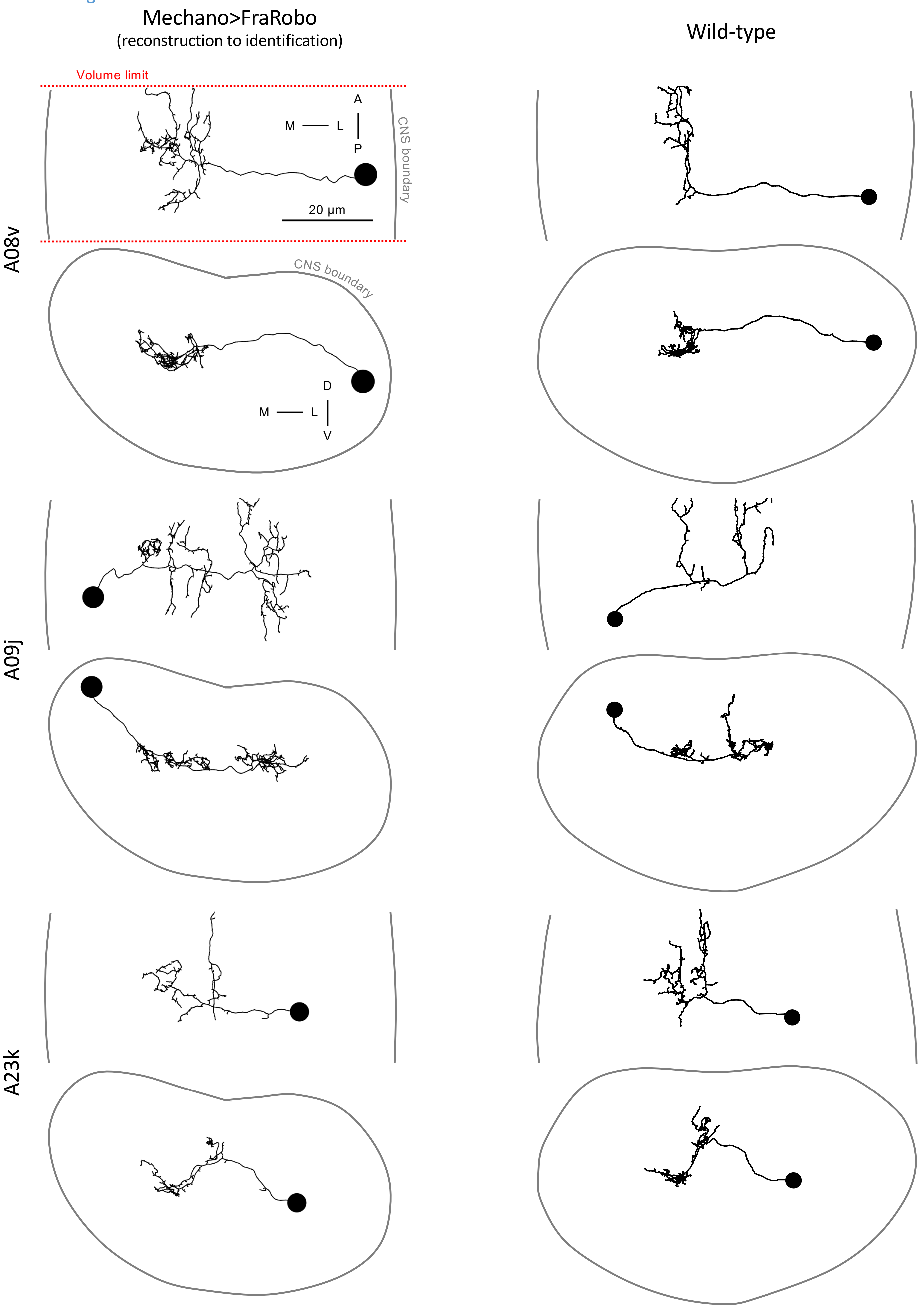

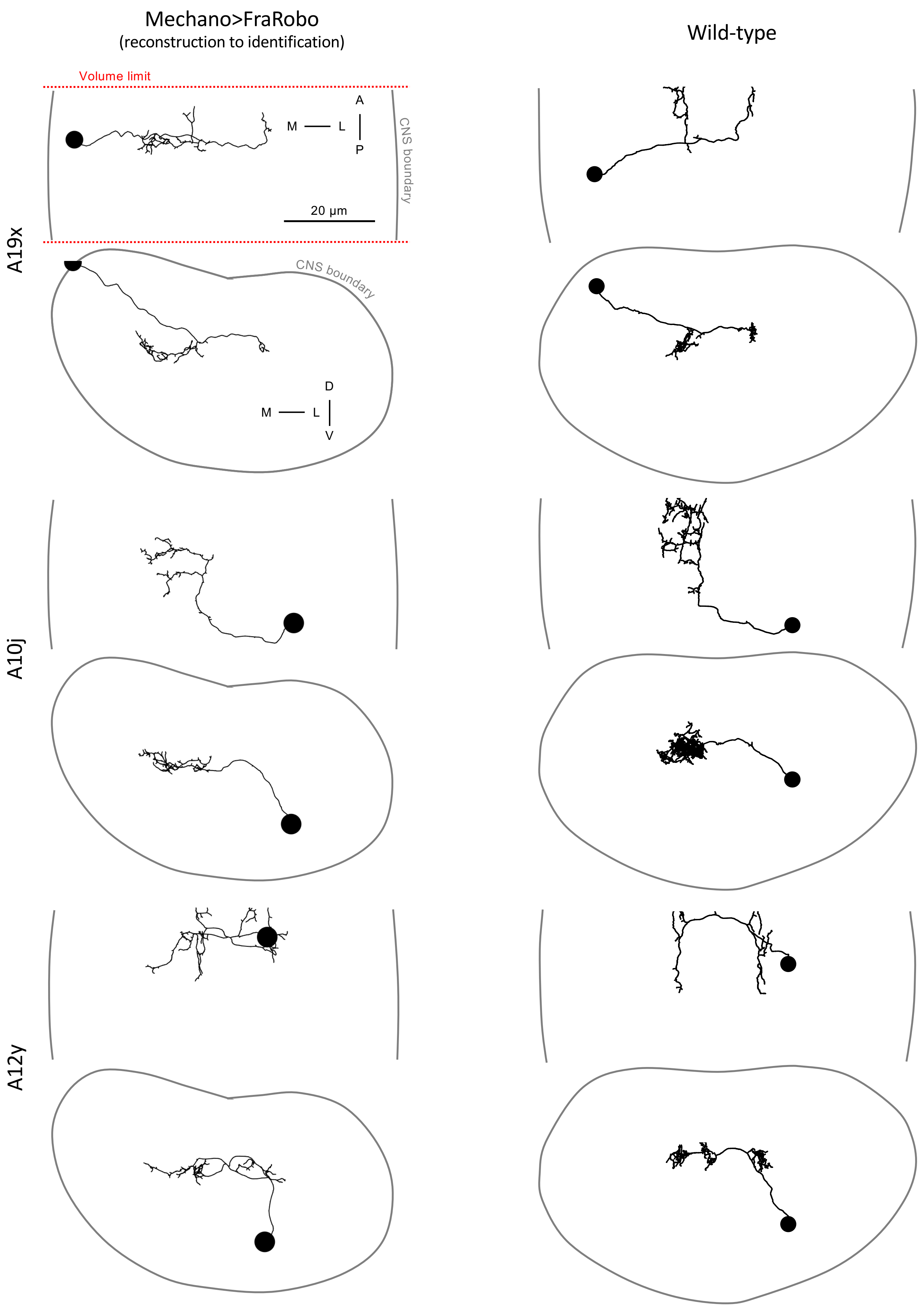
Mechano>FraRobo
(reconstruction to identification)
Wild-type
Volume limit
A
M
L
P
CNS boundary
20 μm
A19x
CNS boundary
D
M
L
V
A10j
A12y

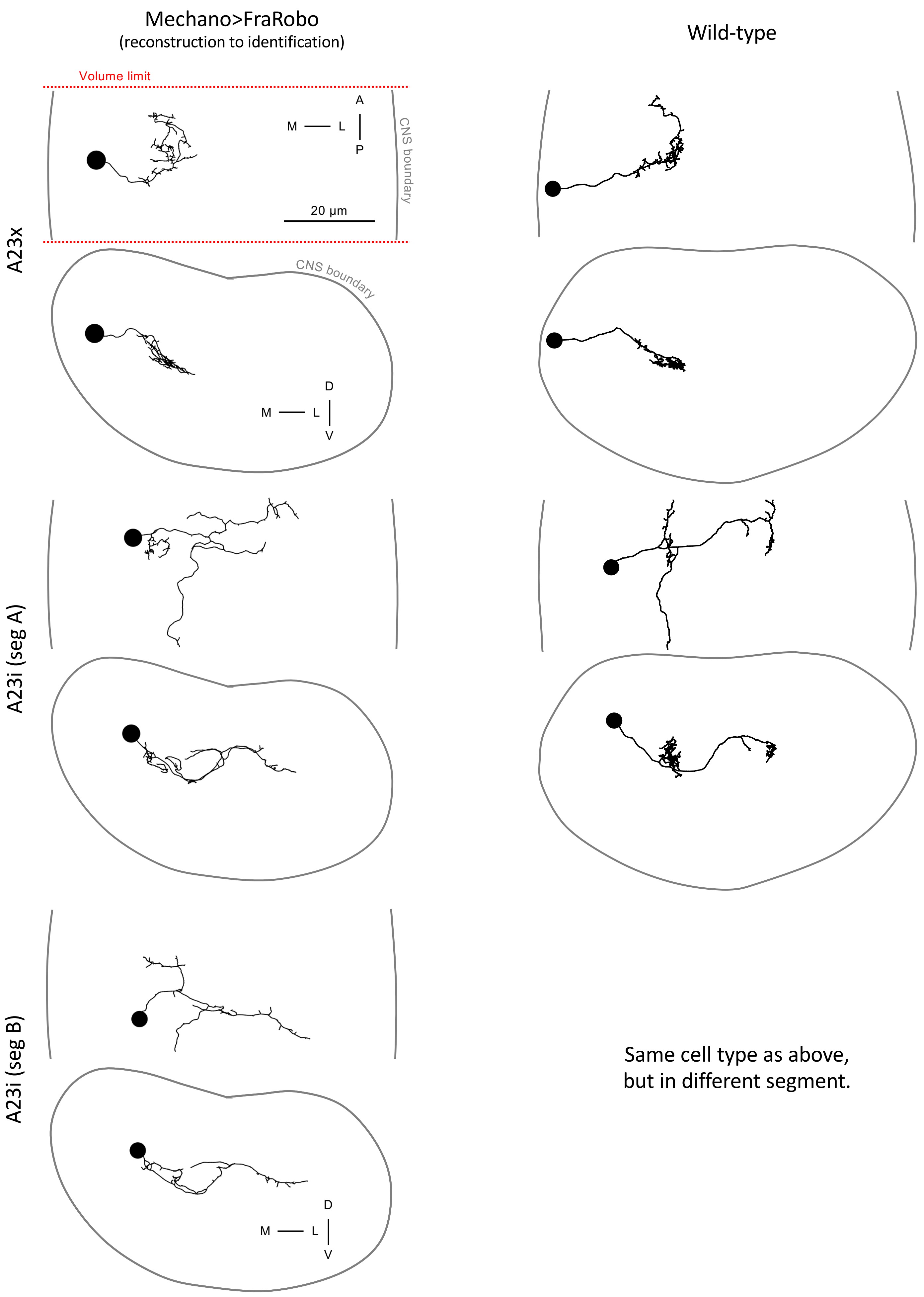
Mechano>FraRobo
(reconstruction to identification)
Wild-type
Volume limit
A
M
L
P
CNS boundary
20 µm
A23x
CNS boundary
D
M
L
V
A23i (seg A)
A23i (seg B)
D
M
L
V
Same cell type as above,
but in different segment.

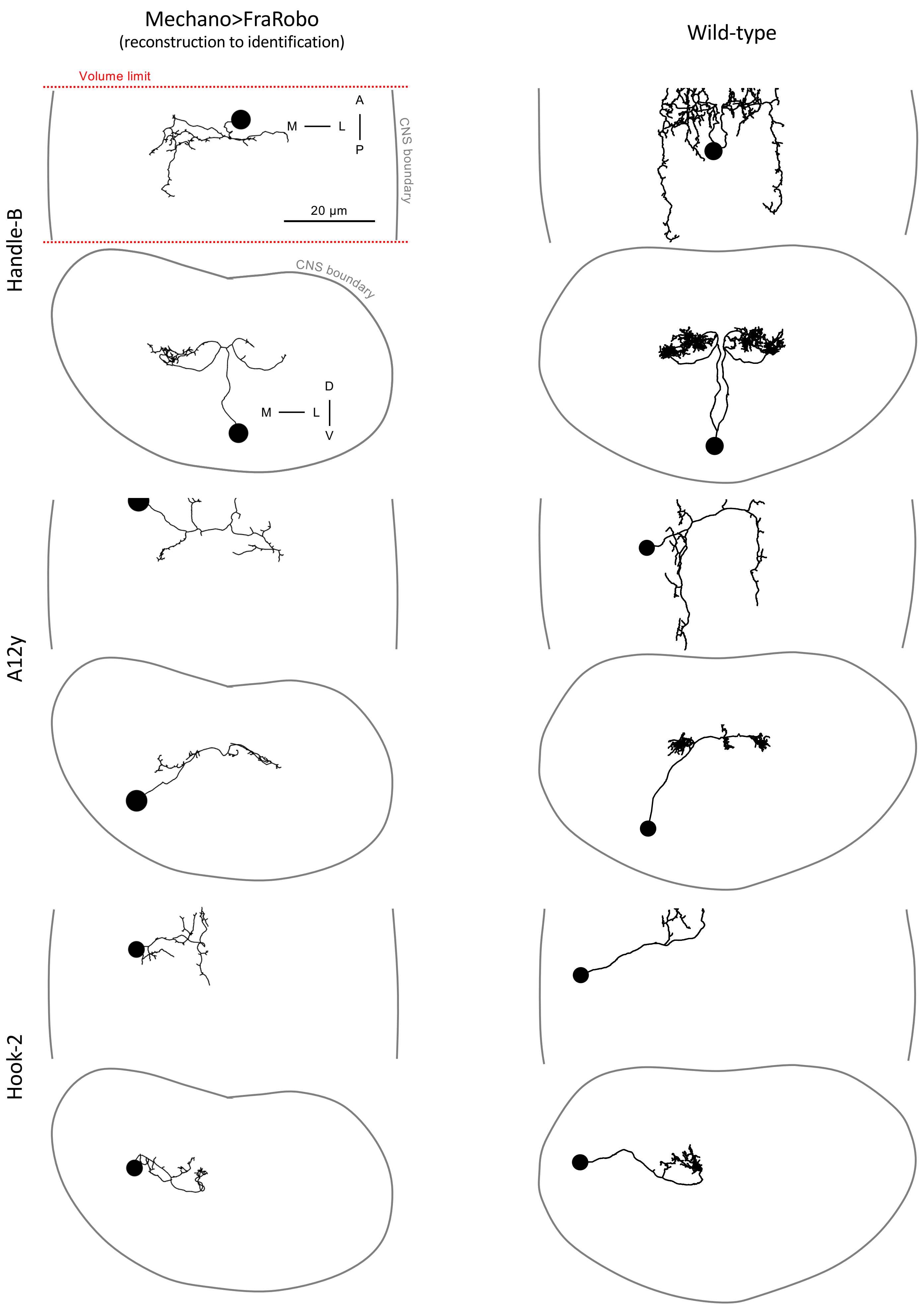

Mechano>FraRobo
(reconstruction to identification)
Wild-type
Volume limit
A
M
L
P
CNS boundary
20 µm
Handle-B
CNS boundary
D
M
L
V
A12y
Hook-2

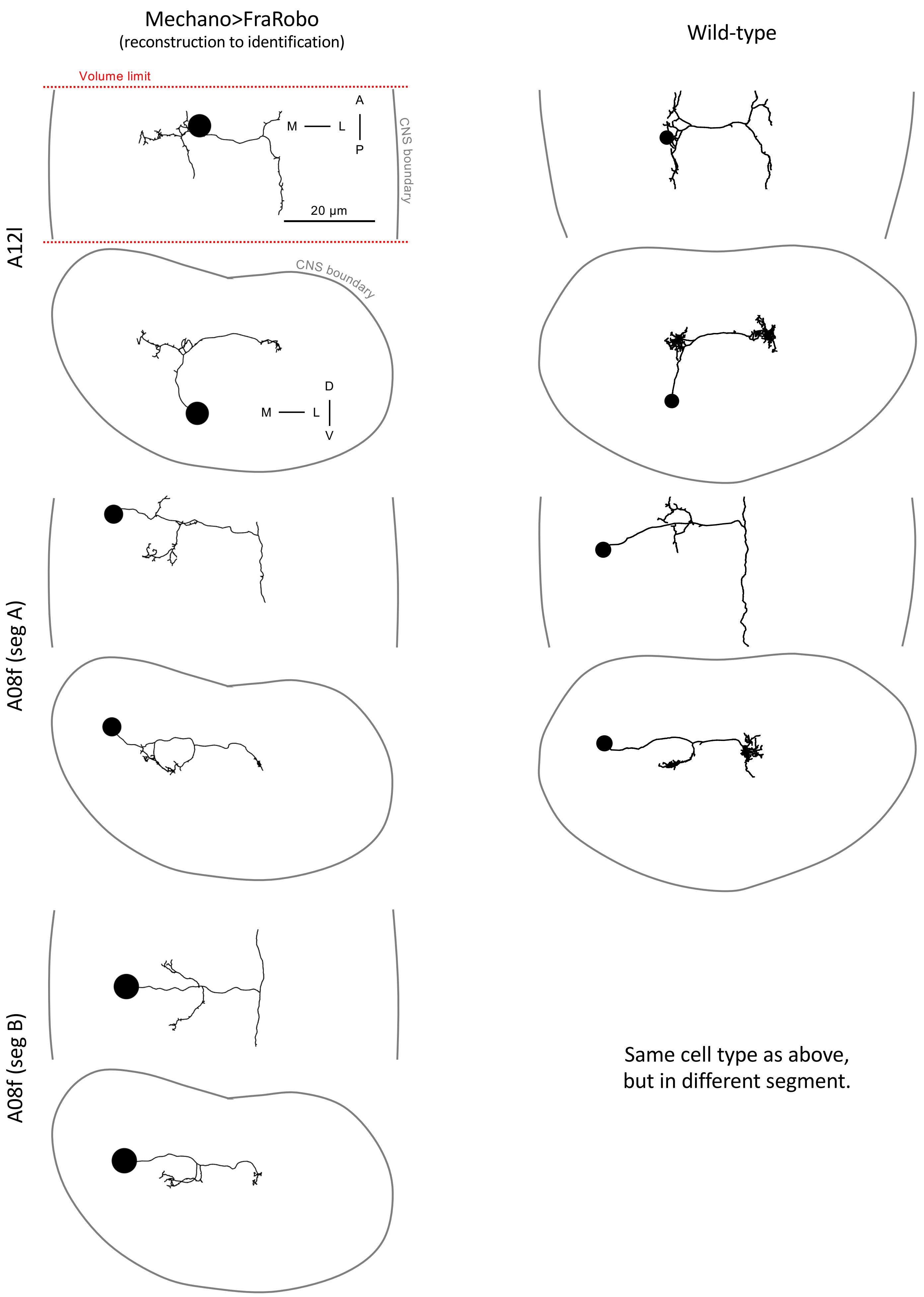

Mechano>FraRobo
(reconstruction to identification)
Wild-type
Volume limit
A
M
L
P
CNS boundary
20 µm
A12l
CNS boundary
D
M
L
V
A08f (seg A)
A08f (seg B)
Same cell type as above,
but in different segment.

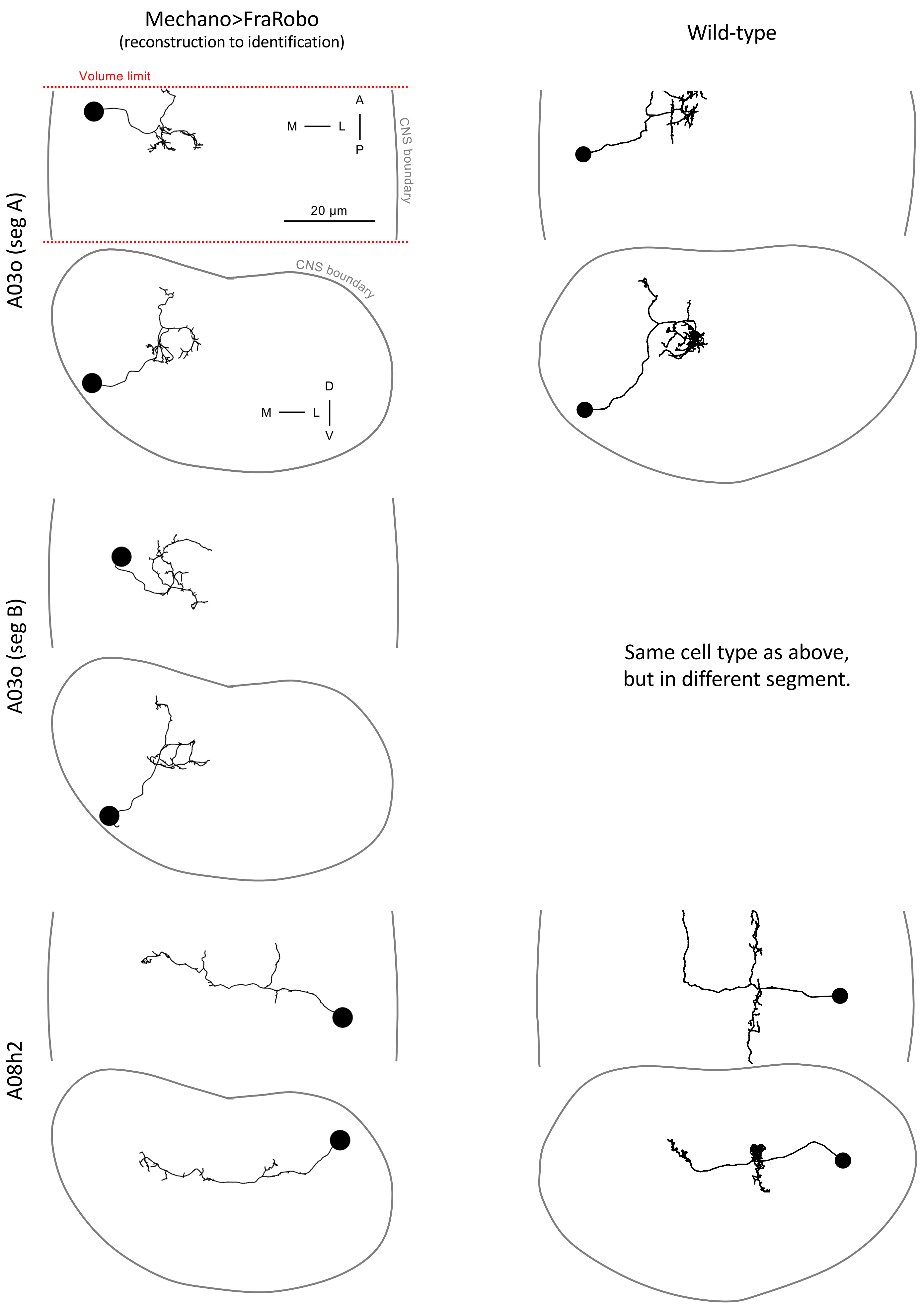

Mechano>FraRobo
(reconstruction to identification)
Wild-type
Volume limit
A
M
L
P
CNS boundary
20 μm
A03o (seg A)
CNS boundary
D
M
L
V
A03o (seg B)
Same cell type as above,
but in different segment.
A08h2

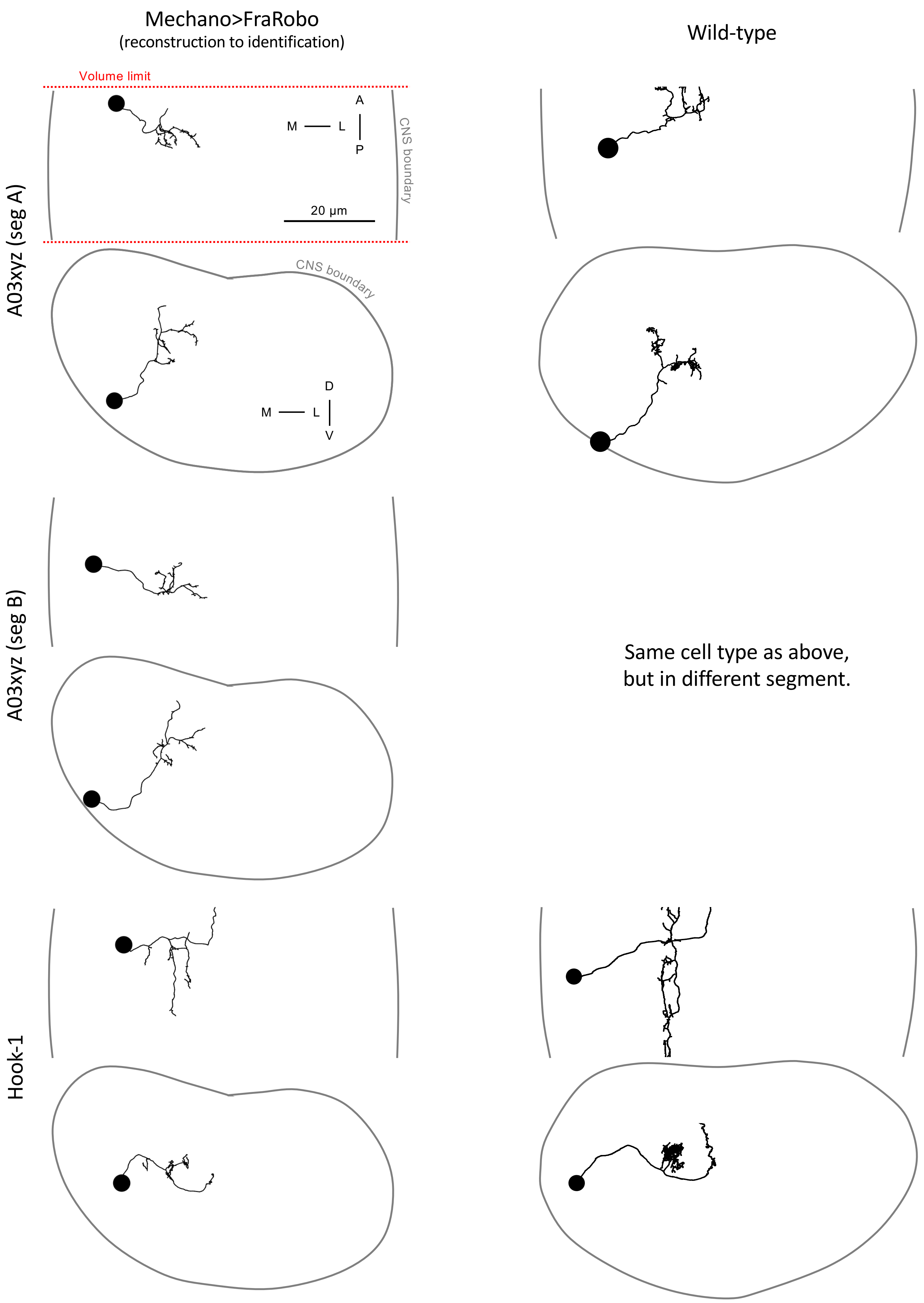
Mechano>FraRobo
(reconstruction to identification)
Wild-type
Volume limit
A
M
L
P
CNS boundary
20 µm
CNS boundary
D
M
L
V
A03xyz (seg A)
A03xyz (seg B)
Same cell type as above,
but in different segment.
Hook-1

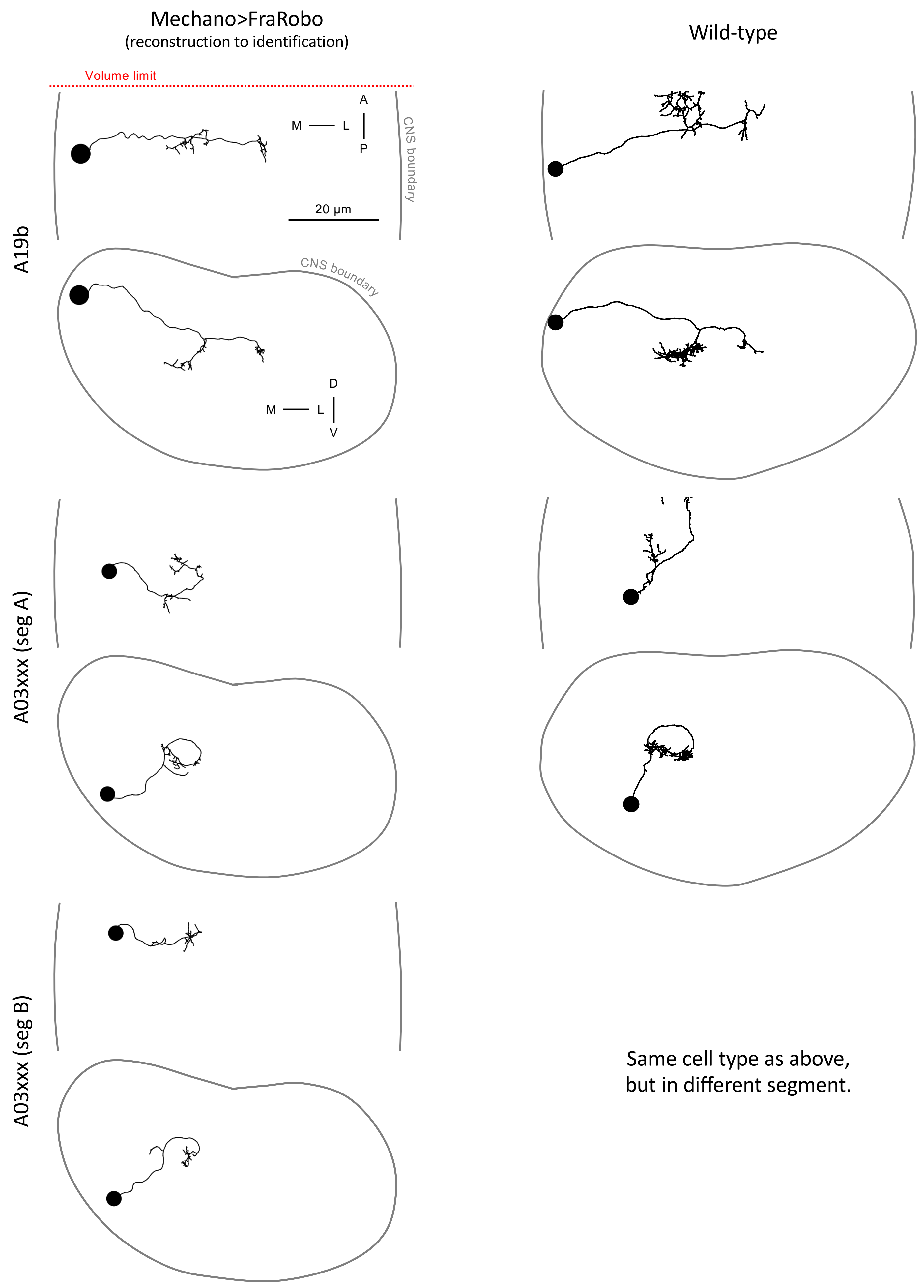

Mechano>FraRobo
(reconstruction to identification)
Wild-type
Volume limit
A
M
L
P
CNS boundary
20 µm
CNS boundary
D
M
L
V
A19b
A03xxx (seg A)
A03xxx (seg B)
Same cell type as above,
but in different segment.

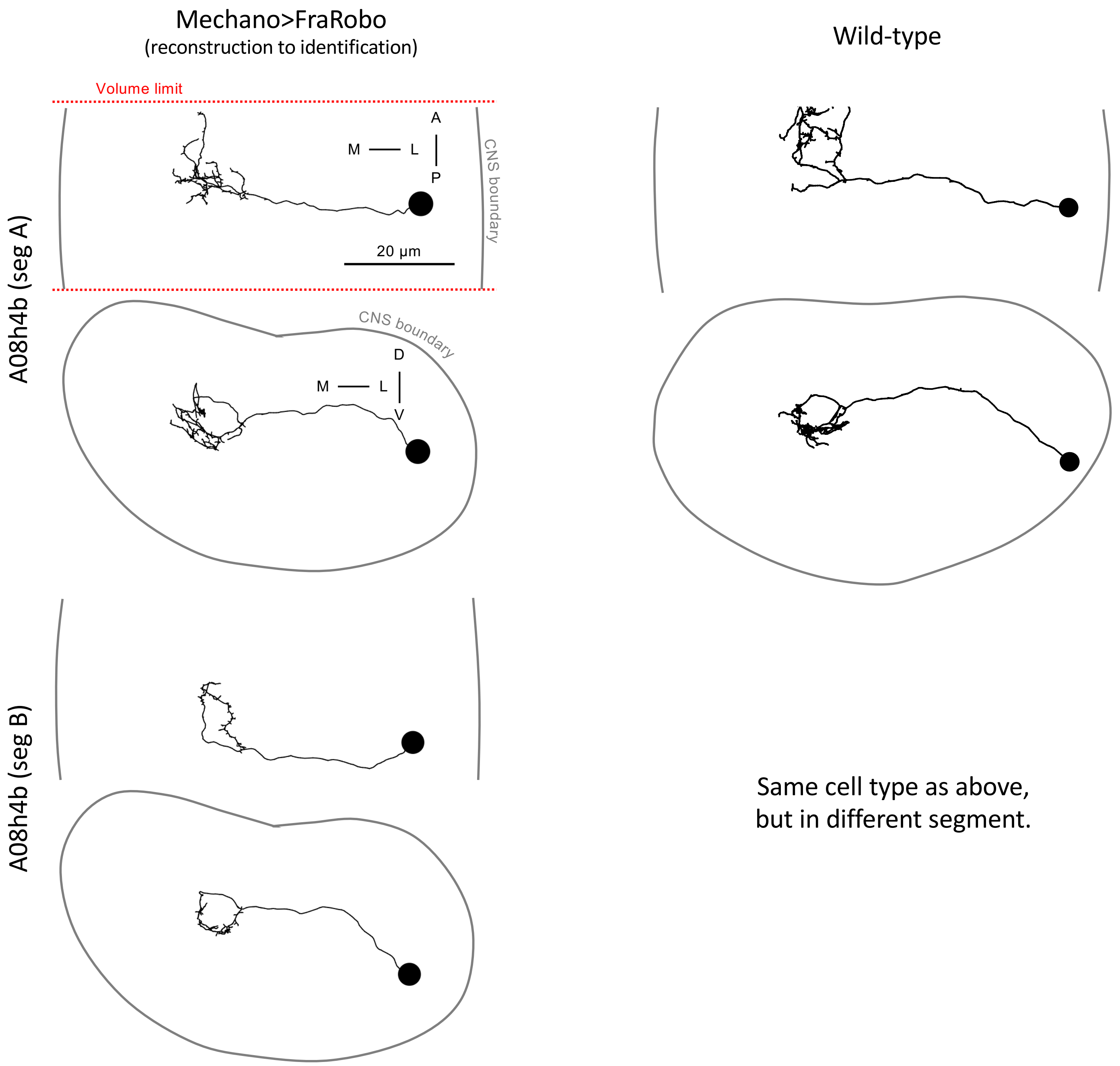

Mechano>FraRobo
(reconstruction to identification)
Wild-type
Volume limit
A
M
L
P
CNS boundary
20 µm
A08h4b (seg A)
CNS boundary
D
M
L
V
A08h4b (seg B)
Same cell type as above,
but in different segment.

Data S3. Neuronal atlas of mechanosensory neurons and preferred partners in the mechano>TNT EM volume. Related to Figures 6 and 7.

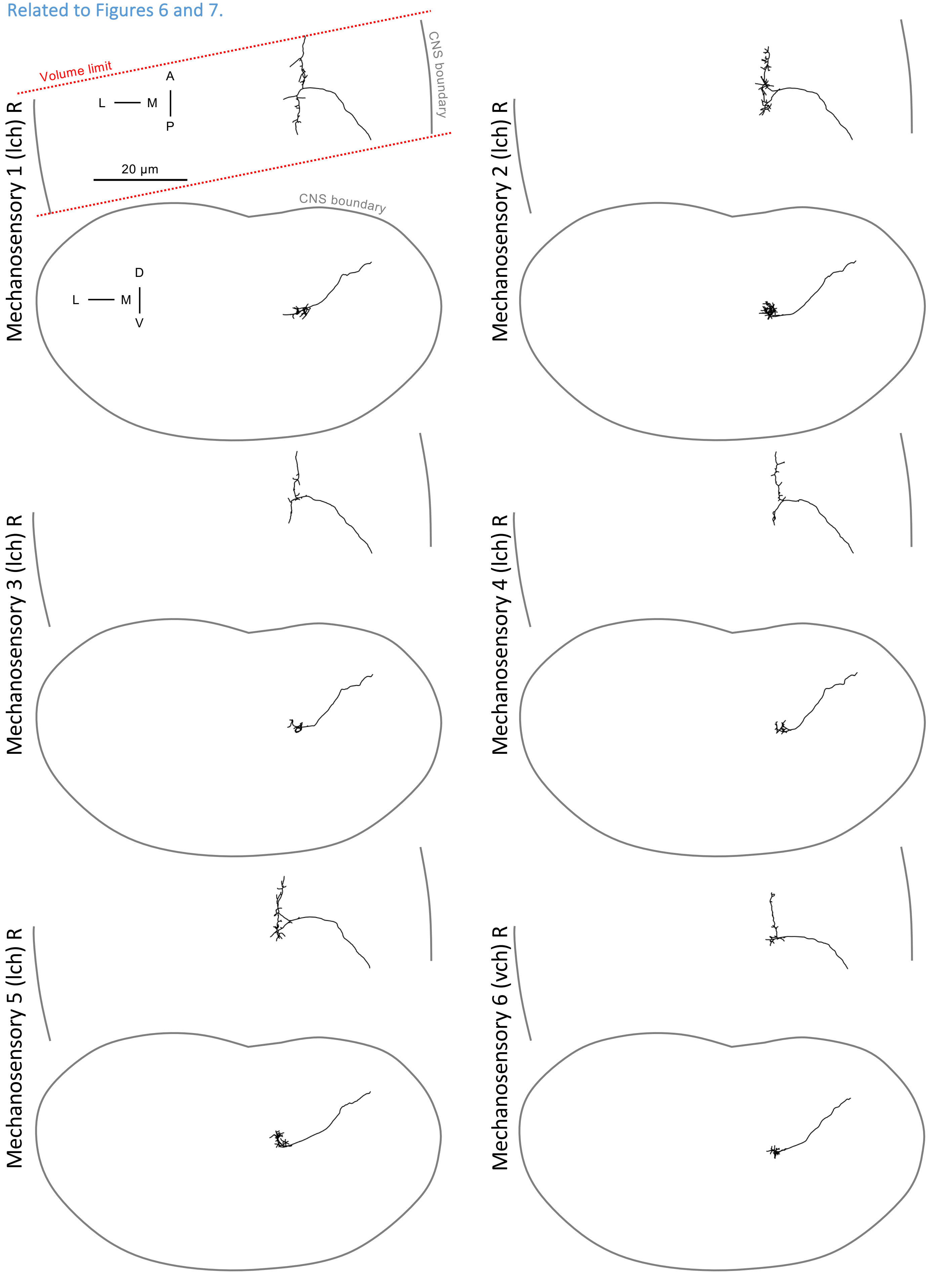

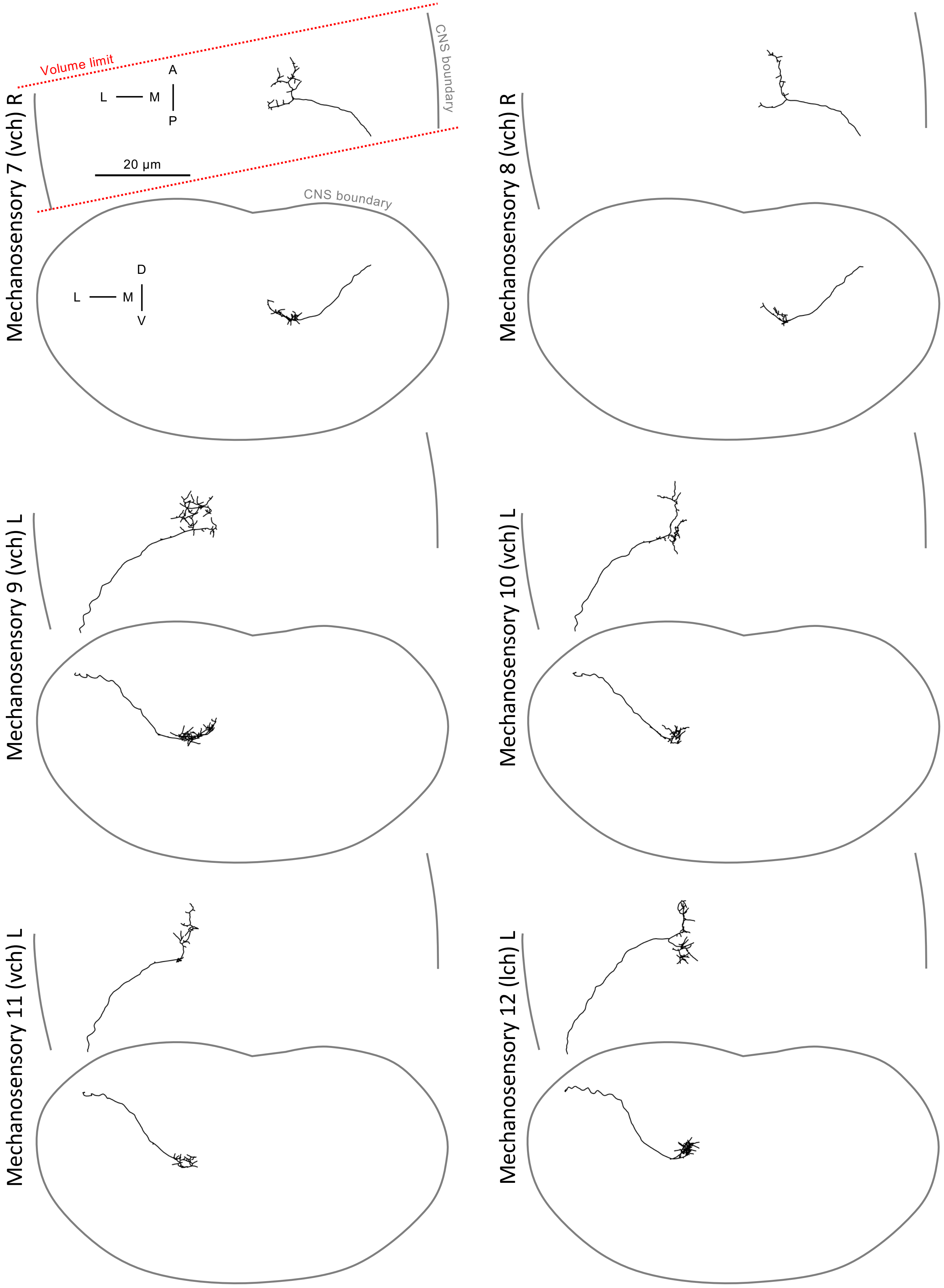

Mechanosensory 7 (vch) R
Volume limit
A
L
M
P
20 µm
CNS boundary
CNS boundary
D
L
M
V
Mechanosensory 8 (vch) R
Mechanosensory 9 (vch) L
Mechanosensory 10 (vch) L
Mechanosensory 11 (vch) L
Mechanosensory 12 (lch) L

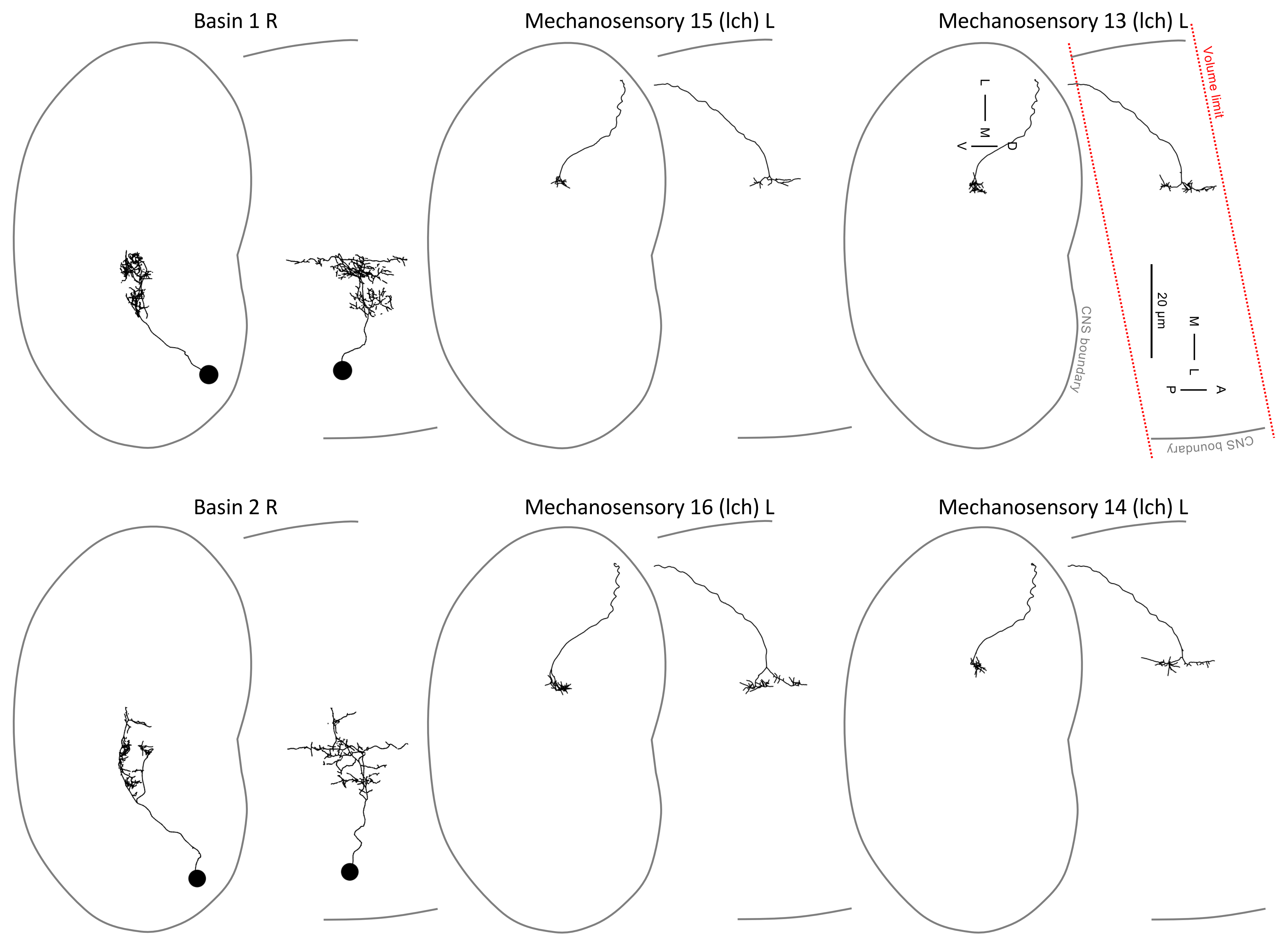
Basin 1 R
Mechanosensory 15 (lch) L
Mechanosensory 13 (lch) L
Volume limit
CNS boundary
CNS boundary
20 µm
M
L
A
P
D
V
Basin 2 R
Mechanosensory 16 (lch) L
Mechanosensory 14 (lch) L

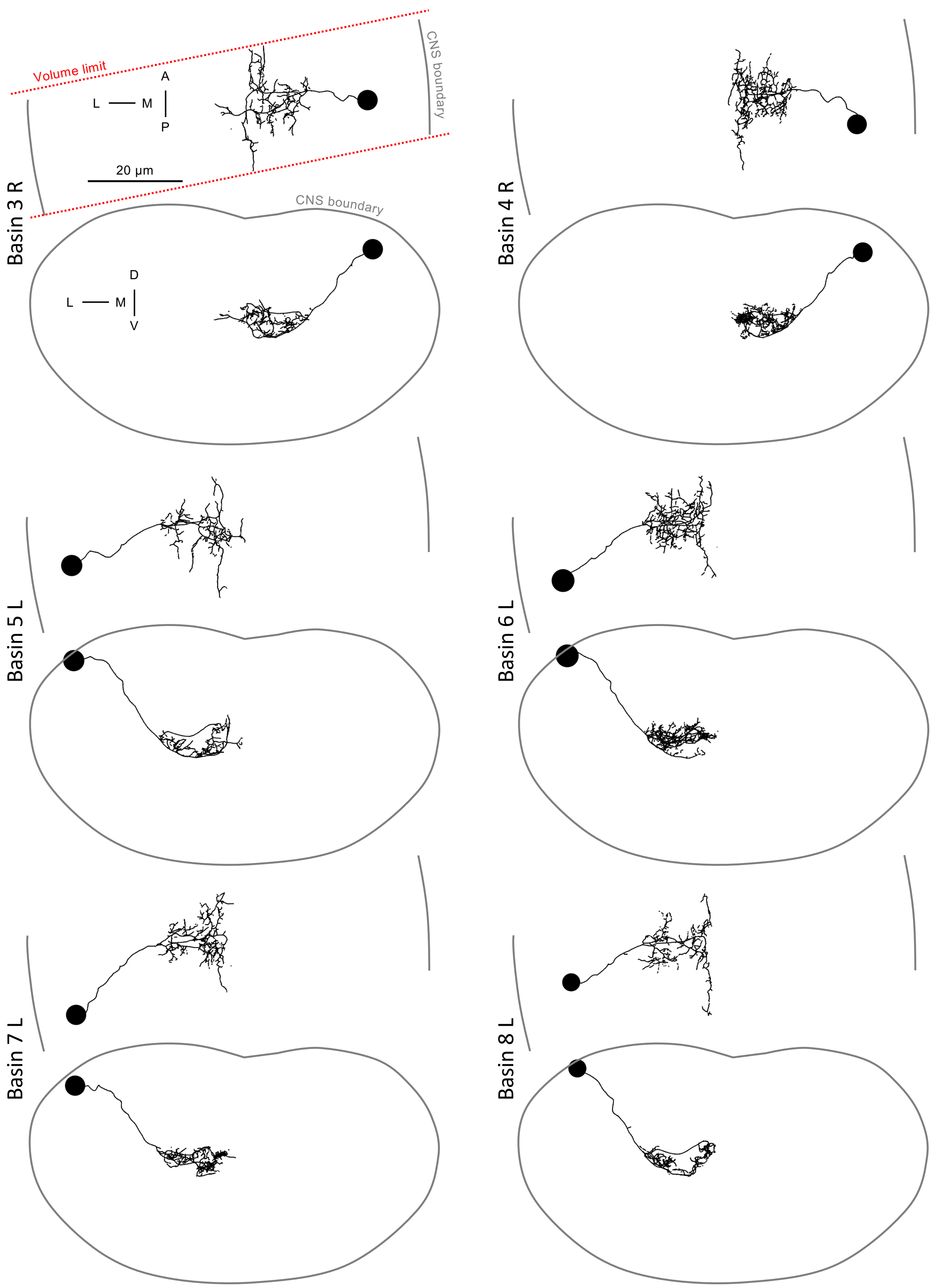
Volume limit
A
L
M
P
20 µm
CNS boundary
CNS boundary
D
L
M
V
Basin 3 R
Basin 4 R
Basin 5 L
Basin 6 L
Basin 7 L
Basin 8 L

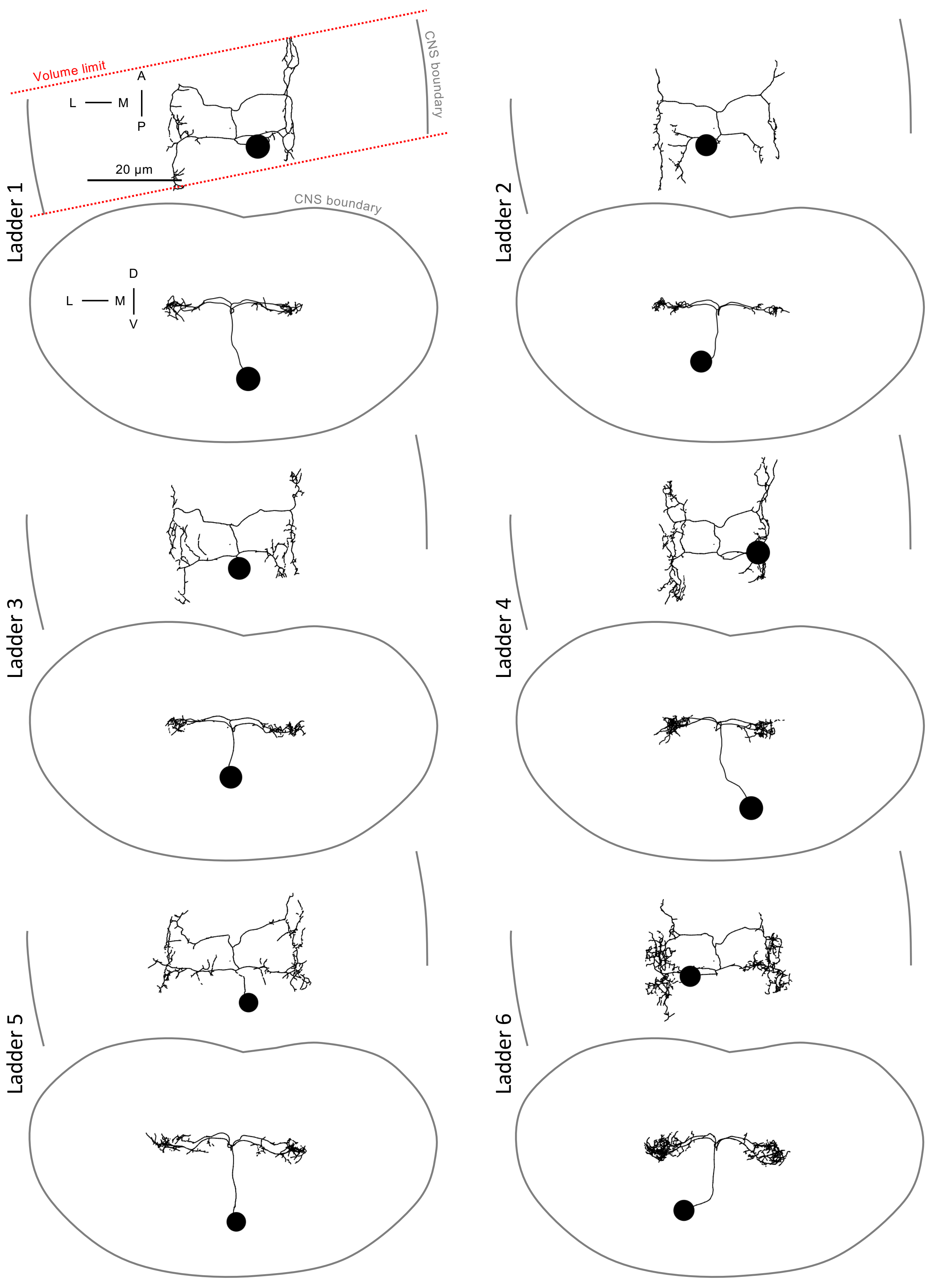

Volume limit
CNS boundary
A
L
M
P
20 µm
D
V
Ladder 1
Ladder 2
Ladder 3
Ladder 4
Ladder 5
Ladder 6

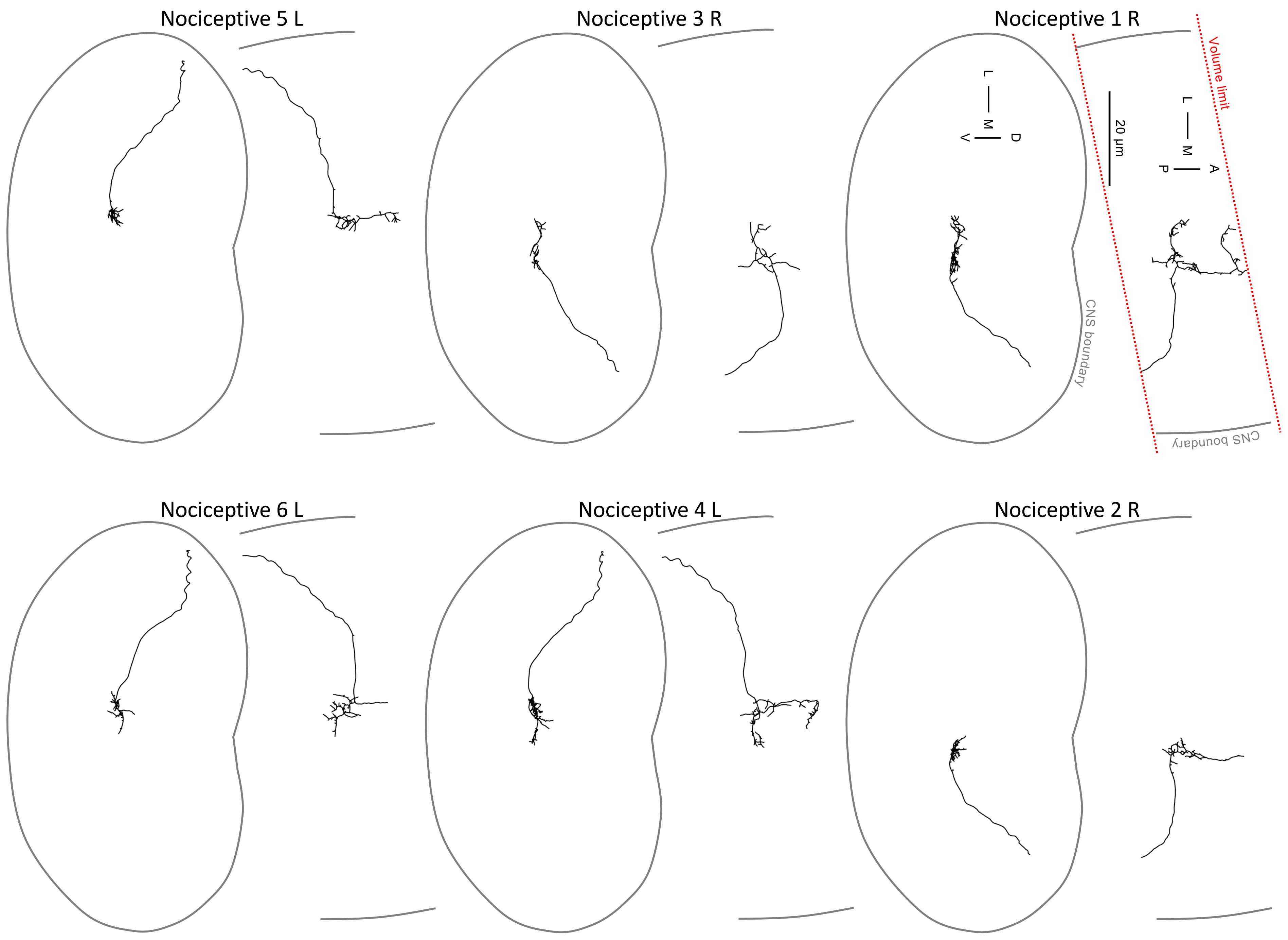

Nociceptive 5 L
Nociceptive 3 R
Nociceptive 1 R
Volume limit
CNS boundary
CNS boundary
20 µm
L
M
D
V
A
P
Nociceptive 6 L
Nociceptive 4 L
Nociceptive 2 R

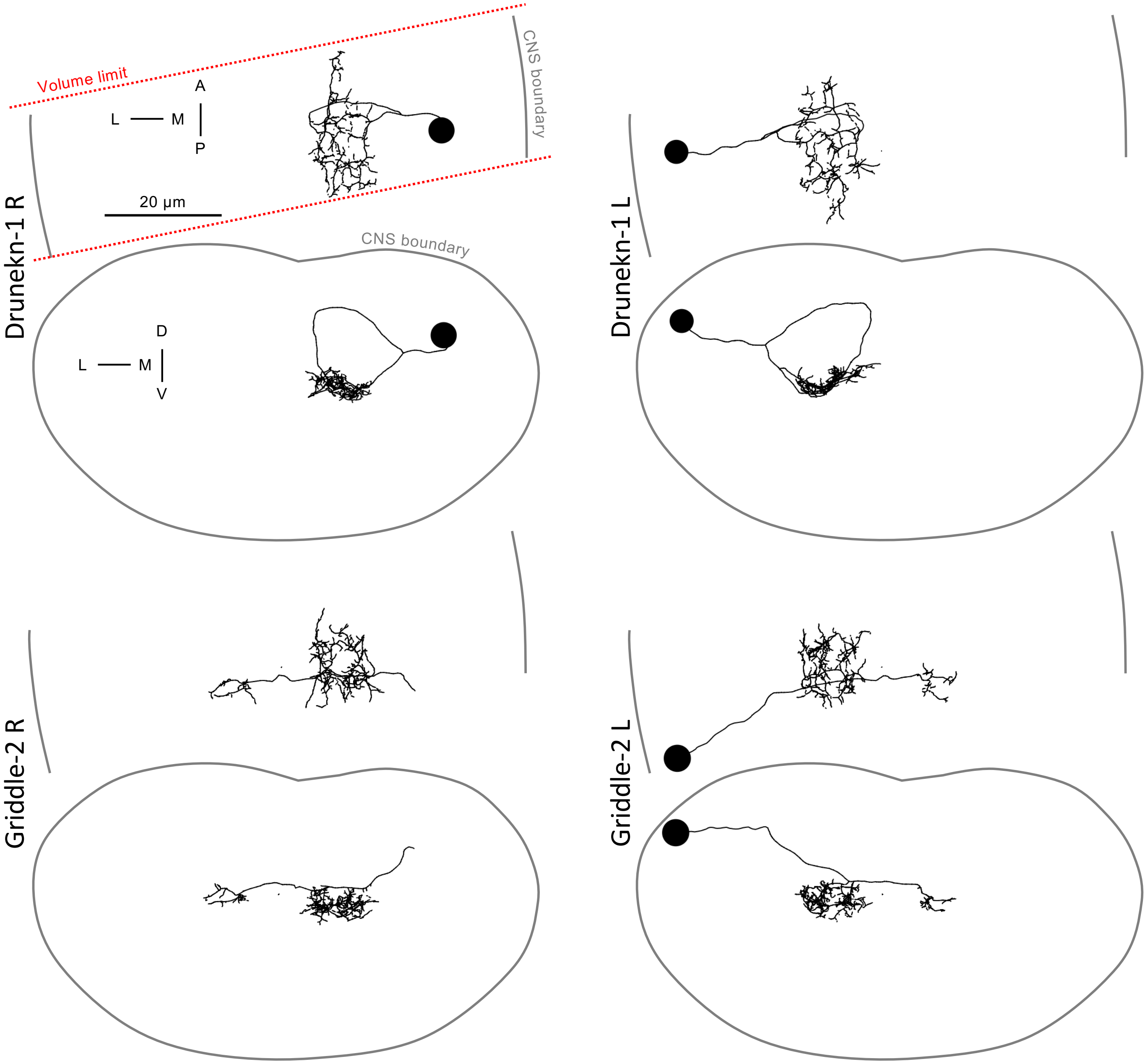
Volume limit
A
L
M
P
20 µm
CNS boundary
CNS boundary
D
L
M
V
Drunekn-1 R
Drunekn-1 L
Griddle-2 R
Griddle-2 L
